# Reinforcing Protein Biochemistry: A Two-Week Experiment Studying Iron(III) Binding by the Transferrin Protein through Stoichiometric Determination, Stability Analysis, and Visualization of the Binding Site

**DOI:** 10.1021/acs.jchemed.3c01016

**Published:** 2024-03-26

**Authors:** Josué A. Benjamín-Rivera, Mariela Pérez Otero, Arthur D. Tinoco

**Affiliations:** † Department of Chemistry, University of Puerto Rico, Río Piedras Campus, Río Piedras, Puerto Rico 00931, United States; ‡ Department of Biology, University of Puerto Rico, Río Piedras Campus, Río Piedras, Puerto Rico 00931, United States

**Keywords:** Upper-Division Undergraduate, Biochemistry, Laboratory Instruction, Computer-Based Learning, Hands-On Learning, Coordination Compounds, Electrophoresis, Proteins, Quality Analysis, UV−Vis Spectroscopy, Metal Binding Site, Transferrin, Conformation Change, Urea-PAGE, Stoichiometry, PyMOL

## Abstract

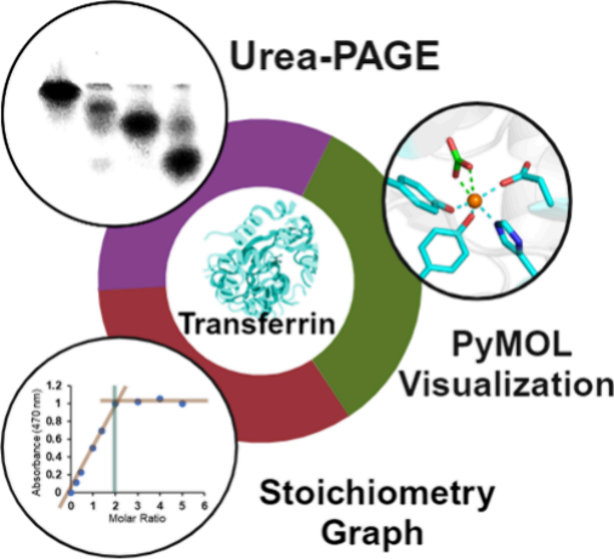

The two-week protein biochemistry experience described herein focuses on reinforcing key biochemical concepts and achieving significant learning domain accomplishments for students (Content Knowledge, Logical Mathematical Reasoning, Visualization, Information Literacy, and Knowledge Integration) and valuable teaching opportunities for instructors. The experience encompasses an exploration of the transport protein serum transferrin as an important regulator of Fe(III) biochemistry and incorporates techniques to assess protein–metal stoichiometry and protein stability and to perform molecular visualization. Students gain practical experience in utilizing spectrophotometric analysis for constructing stoichiometric curves, in performing urea-PAGE, and in applying the PyMOL program to evaluate metal coordination at a protein binding site and the associated protein structural change. The learning and teaching accomplishments provide valuable skills that can be extended into research and translated to other teaching formats.

## Introduction

**Figure 1:**
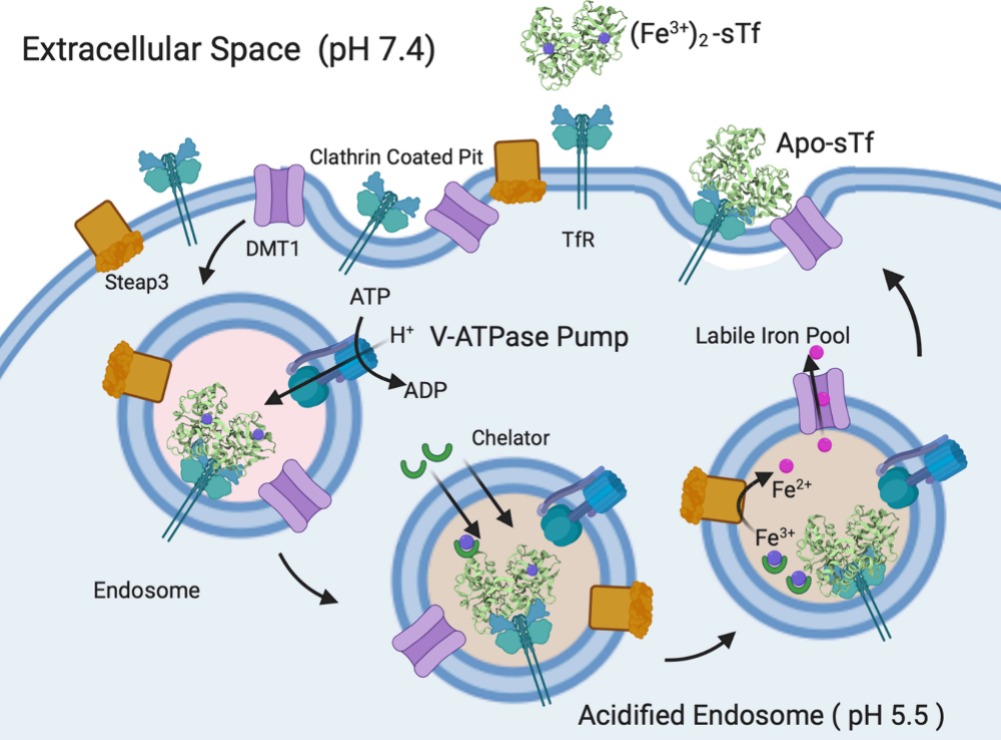
Cell uptake of Fe_2_-sTf and Fe release into the cytosol via the TfR1 endocytosis pathway. This is a pH-dependent process. Fe_2_-sTf binds to TfR1, which triggers endosome uptake into mammalian cells. A combination of pH drop and intracellular chelation removes Fe(III) from sTf. The metal ion is reduced to Fe(II) by the Steap3 reductase, which allows it to be released into the cytosol via the divalent metal transporter 1 (DMT1) and enter the labile iron pool. Apo-sTf remains bound to TfR1 and returns to the membrane to be recycled for additional rounds of Fe(III) cellular uptake. This figure was created with BioRender.

Serum transferrin (sTf), an 80 kDa glycoprotein,^1^ is one of the most studied proteins. It is present in blood at levels of 30–60 μM and plays major roles in maintaining iron homeostasis and distributing iron throughout the body^2,3^ and thus participates in many regulatory functions.^3,4^ By binding Fe(III) upon its release into the bloodstream, hydrolysis and precipitation of the metal ion are prevented, and as a result, the blood solubility of Fe(III) is at micromolar levels, much higher than the *K*_sp_ would predict. This increased solubility promotes its bioavailability. sTf also inhibits Fe(III) reduction to Fe(II) in blood, preventing uncontrolled generation of reactive oxygen species.^5^ It also participates in bacteriostasis by preventing Fe(III) capture by pathogenic bacteria as part of the body’s innate immunity.^6,7^ The protein is the major molecular pathway for Fe(III) delivery to cells throughout the body via metal uptake transferrin receptor 1 (TfR1)-mediated endocytosis (Figure 1 and Supporting Information A).^8−11^

**Figure 2:**
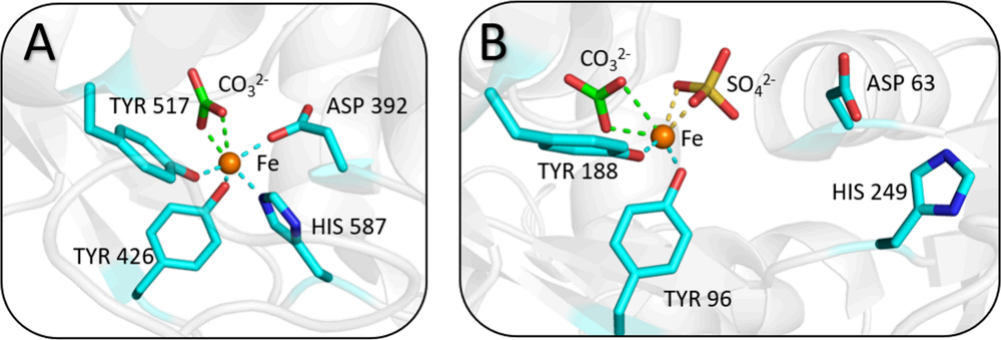
Fe(III) binding site of the (A) C-lobe and (B) N-lobe of sTf generated by the PyMOL program using PDB entry 3QYT. The C-lobe demonstrates the closed conformation, involving canonical coordination of Fe(III) bound by two tyrosines (in tyrosinate form), one aspartate, and one histidine and the bidentate synergistic anion carbonate. The N-lobe demonstrates an open conformation, involving noncanonical coordination of Fe(III) bound by two tyrosines, bidentate carbonate, and bidentate sulfate (a non-carbonate anion). The presence of the sulfate prevents the coordination of the aspartate and histidine.

The Fe(III) coordination by sTf has been extensively characterized by X-ray crystallography. STf possesses two Fe(III) binding sites in its N-terminal and C-terminal lobes (N-lobe and C-lobe). The primary coordination spheres (direct atom coordination) of the two Fe(III) sites are identical. They consist of Fe(III) bound by two tyrosines, an aspartic acid, a histidine, and the synergistic anion carbonate (CO_3_^2–^) in bidentate modality^12^ (Figure 2A). The amino acid residues of the N-lobe are Asp63, Tyr95, Tyr188, and His249, and those of the C-lobe are Asp392, Tyr426, Tyr517, and His585. When Fe(III) is bound exclusively to the N- or C-lobe, the protein is denoted here as Fe_N_-sTf or Fe_C_-sTf, respectively. Fe(III)-saturated sTf is denoted here as Fe_2_-sTf. Important differences in the second coordination spheres (the sphere that influences the stability of the primary coordination sphere but does not involve any direct atom coordination to the metal ion) contribute to the difference in the relative stability of Fe(III) bound by the N- and C-lobes (Supporting Information A).^13−15^

Protein stability is a critical aspect of understanding protein structure and function and, in the context of sTf, transport. Denaturants or chemicals that disrupt the native conformation of proteins can be used as tools to investigate protein stability, such as in urea electrophoresis.^16−18^ Denaturants destabilize proteins by disrupting noncovalent interactions, such as hydrogen bonds, that maintain their folded structure. Protein stability plays a crucial role in the metal delivery function of sTf, and its stability can be readily assessed in terms of its Fe(III) binding, which results in a dramatic conformational change. The metal-binding amino acids are located at opposite sides of each lobe, and as they capture the Fe(III), they draw the lobes to close. In addition to high Fe(III) affinity (log *K*_C-lobe_ = 22.2 and log *K*_N-lobe_ = 21.3),^19,20^ the closed conformations are supported by the N-lobe dilysine interaction and by the C-lobe triad amino acid interaction.^21−25^ The protein conformation closure increases the protein’s stability. Fe(III) binding to one site results in the stabilization of the other site without bound Fe(III), indicative of lobe cooperativity^26^ similar to the subunit cooperativity observed when O_2_ binds to the heme groups in hemoglobin. The fully lobe-closed Fe(III) binding is termed the canonical form of Fe(III) coordination by sTf (Figure 2A). The presence of non-carbonate synergistic anions can cause noncanonical forms of Fe(III) coordination in an open conformation (Figure 2B).^11,27^ Urea polyacrylamide gel electrophoresis (urea-PAGE) can distinguish metal-free (apo) protein forms, Fe(III) bound to each protein site, and Fe(III)-saturated (holo) sTf. The apo protein travels least through the gel because it is the least stable form of the protein, and the holo protein travels farthest.^28^ The stabilities range in the following order: apo-sTf < Fe_C_-sTf < Fe_N_-sTf < Fe_2_-sTf.^14,15^ Structural visualization by methods such as X-ray crystallography and small-angle X-ray scattering enable researchers to study the protein’s conformation changes due to Fe(III) binding,^23,24^ which are essential for holo-sTf recognition by TfR1.^29^ Fully lobe-closed metal-bound sTf maximizes cell uptake of the metal.^30,31^

Given the wealth of information available for sTf and the pivotal role it plays in Fe biochemistry, the protein is an excellent model for reinforcing a myriad of biochemical concepts. With sTf serving as an indicator of Fe blood levels, Brumaghim et al. designed a laboratory experience focused on chelator-induced release of Fe from sTf for insight into chelation therapy.^32^ Dominguez-Vera et al. created a laboratory experience on the differences in the pH-dependent Fe release from sTf and from lactoferrin associated with differences in protein function.^33^ Herein a laboratory experience is presented on sTf binding of Fe(III) and subsequent stabilization of the protein and the visualization of the Fe(III) coordination.

## Learning Objectives

This laboratory experience focuses on protein structure and stability, as influenced by metal binding. We tackle the learning domains of Content Knowledge, Information Literacy, and Knowledge Integration by providing students with references in the lecture material and the postlab report assignment. We teach them logical mathematical reasoning by determining metal–protein binding stoichiometry using a spectroscopic approach.^34−37^ We also provide visualization of protein structure^38^ and comparative stability.^12,13,39−41^ The specific objectives of this laboratory experience are as follows: (1) quantify the stoichiometry of Fe(III) coordination to sTf using spectrophotometry; (2) assess the metal-binding-associated stabilization of sTf by urea-PAGE using a chemical denaturation approach; (3) visualize the 3D structure of Fe(III) coordination to STf using PyMOL. These objectives were realized by having the students perform a series of tasks:

1. Prepare different sTf samples at a set concentration mixed with different Fe(III) equivalents. The formation of the tyrosine to Fe(III) ligand-to-metal charge transfer (LMCT) absorbance is monitored at 470 nm to construct a stoichiometric curve to determine the binding stoichiometry.
2. Prepare Fe_C_-sTf, Fe_N_-sTf, and Fe_2_-sTf and compare their stabilities to that of apo-sTf by urea-PAGE.
3. Use the PyMOL program to study Fe(III) coordination at the C-site (in lab) and at the N-site (postlab report) of the PDB 3QYT structure for insight into the molecular details of the Fe(III) coordination and effect on the protein’s structure.

## Experiment Overview

The lab experience spans two weeks (two periods of 4 hours each). The student lab manual is provided in Supporting Document B, the instructor guide version in Supporting Document C, and a manual with the instructions for using PyMOL in Supporting Document D. The key activities of each week are detailed as follows.

### Week 1

**Figure 3:**
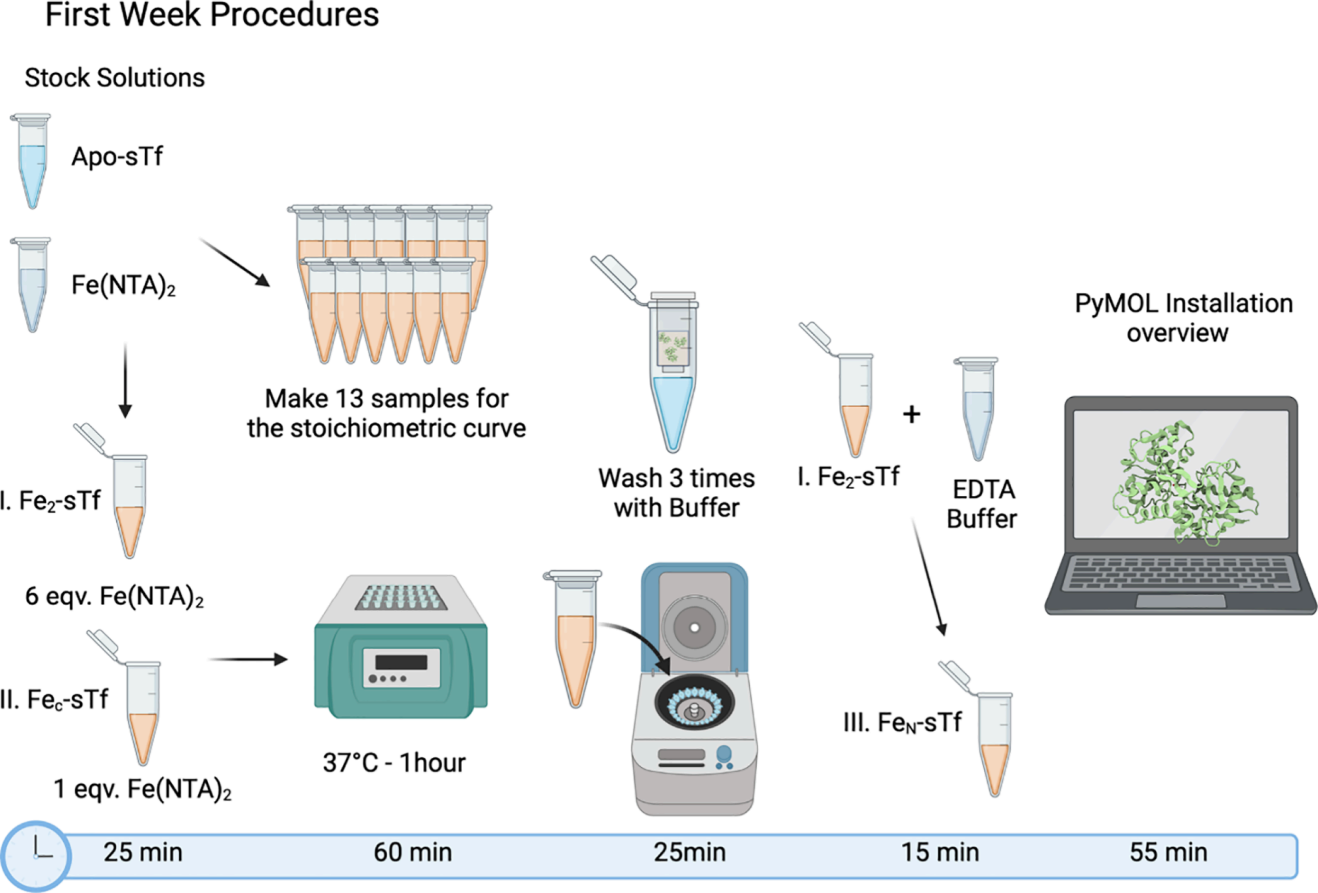
During the first week, students prepared different Fe(III)-bound sTf samples: (I) Fe_2_-sTf, (II) Fe_C_-sTf, and (III) Fe_N_-sTf. First, the students prepared two samples, Fe_2_-sTf and Fe_C_-sTf by reacting apo-sTf with Fe(nitrilotriacetic acid)_2_ (Fe(NTA)_2_) at different mole equivalents (refer to Supporting Document A). The preparation of these samples took approximately 25 min. Next, the students incubated the two prepared samples at 37 °C for 60 min. During this time, they prepared 13 additional Fe(III)-bound sTf samples to construct a stoichiometric curve. Once the stoichiometric curve samples were prepared, they were stored for use during the second week. After the 60 min incubation period, the students removed the samples from heat and eliminated the excess Fe(III) using a rapid spin dialysis membrane (centricon tube) purification process, which took approximately 25 min. For the preparation of Fe_N_-sTf (sample III), the students used the washed Fe_2_-sTf sample. Half of this sample was combined with the buffer containing the hexadentate chelator ethylenediaminetetraacetic acid (EDTA), and the process took approximately 15 min. In the final 55 min, the students installed and were introduced to the PyMOL program and its various components and features. This figure was created with BioRender.

During the first week (Figure 3), the students received a 1 hour overview lecture (Supporting Document E) that explained the regulatory roles that sTf plays with regard to Fe(III) homeostasis and biodistribution and discussed the structural details of Fe(III) binding. The students then entered the laboratory room to prepare different samples of Fe(III)-bound sTf with different amounts of metal ion to construct a stoichiometric curve for determining the stoichiometry of bound Fe(III) and for the urea-PAGE. Due to the time required for these solutions to reach equilibrium (>1 h), the solutions were left to react in a 4 °C refrigerator until the week 2 session. At the end of the session they were provided instructions to download the PyMOL program.

#### Week 1 Learning Goals

The students learned to prepare Fe_C_-sTf, Fe_N_-sTf, and Fe_2_-sTf. While the students prepared the samples, they visually observed salmon color formation due to Fe(III) coordination. They were briefly introduced to PyMOL, a powerful tool for visualizing and analyzing protein structures.

### Week 2

**Figure 4:**
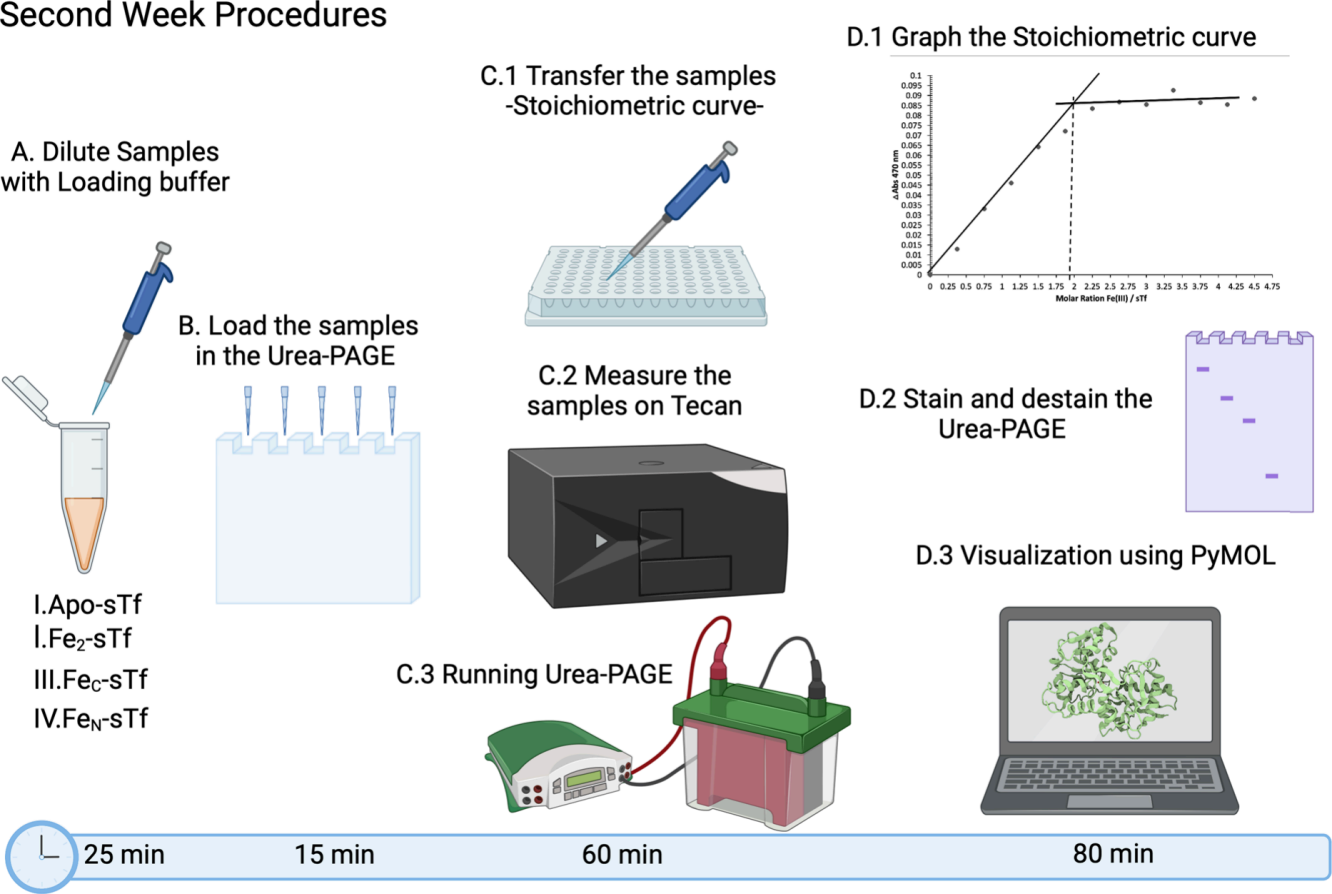
(A) During the second week, students analyzed four samples (apo-sTf, Fe_C_-sTf, Fe_N_-sTf, and Fe_2_-sTf) by urea-PAGE. These samples were diluted with buffer and mixed with the loading buffer. (B) The prepared samples were then added to the gel for urea-PAGE. (C) The urea gel was run for 60 min. The urea gel setup was followed by transferring the 13 samples of Fe(III)-bound sTf containing different Fe(III) equivalents onto a 96-well plate for absorbance measurement at 470 nm using a plate reader. (D) The stoichiometric graph was then generated to determine the number of Fe(III) ions bound to sTf. After the urea gel was destained using a destaining solution, the students proceeded to complete the PyMOL assignment. Once the PyMOL presentation was finished, a picture of the urea gel was taken. This figure was created with BioRender.

Two hours before the start of the second week session, all samples from Week 1 were left to equilibrate to room temperature. In the second week (Figure 4), the students used a plate reader (Tecan) to measure the absorbance at 470 nm of the apo-sTf and Fe(III)-bound samples. This absorbance is the source of the salmon color and is due to a tyrosine to Fe(III) LMCT band with an extinction coefficient of 5000 M^–1^ cm^–1^ based on Fe_2_-sTf concentration and 2500 M^–1^ cm^–1^ based on Fe(III). The students also set up urea-PAGE with the apo-sTf, Fe_C_-sTf, Fe_N_-sTf, and Fe_2_-sTf samples. The urea-PAGE technique is a simple technique that provides an easy readout of information regarding protein stability. This gel consists of 3% acrylamide in the stacking gel and 7% acrylamide in the resolving gel, with 7 M urea in both. While the urea-PAGE ran for 60 min, the students received a lecture to learn how to use PyMOL to visualize Fe(III) coordination at the sTf C-lobe (Supporting Documents D and F).

#### Week 2 Learning Goal

The students learned to construct a stoichiometric curve by plotting the absorbance at 470 nm versus the metal:sTf mole ratio and then used the data to determine the Fe(III) stoichiometry. The expectation was that two Fe(III) ions will saturate the protein. They also observed the relative stability of the protein samples by urea-PAGE. The further down the gel, the more stable the protein sample is. The stabilities were in the order apo-sTf < Fe_C_-sTf < Fe_N_-sTf < Fe_2_-sTf. Furthermore, the students gained some basic comprehension of coordination chemistry concepts, including hard/soft acid/base theory and electronic transitions.^12^ They reviewed several techniques and used associated instrumentation, including electrophoresis, rapid spin dialysis for protein cleaning, and UV–vis spectroscopy.

## Results and Discussion

**Figure 5:**
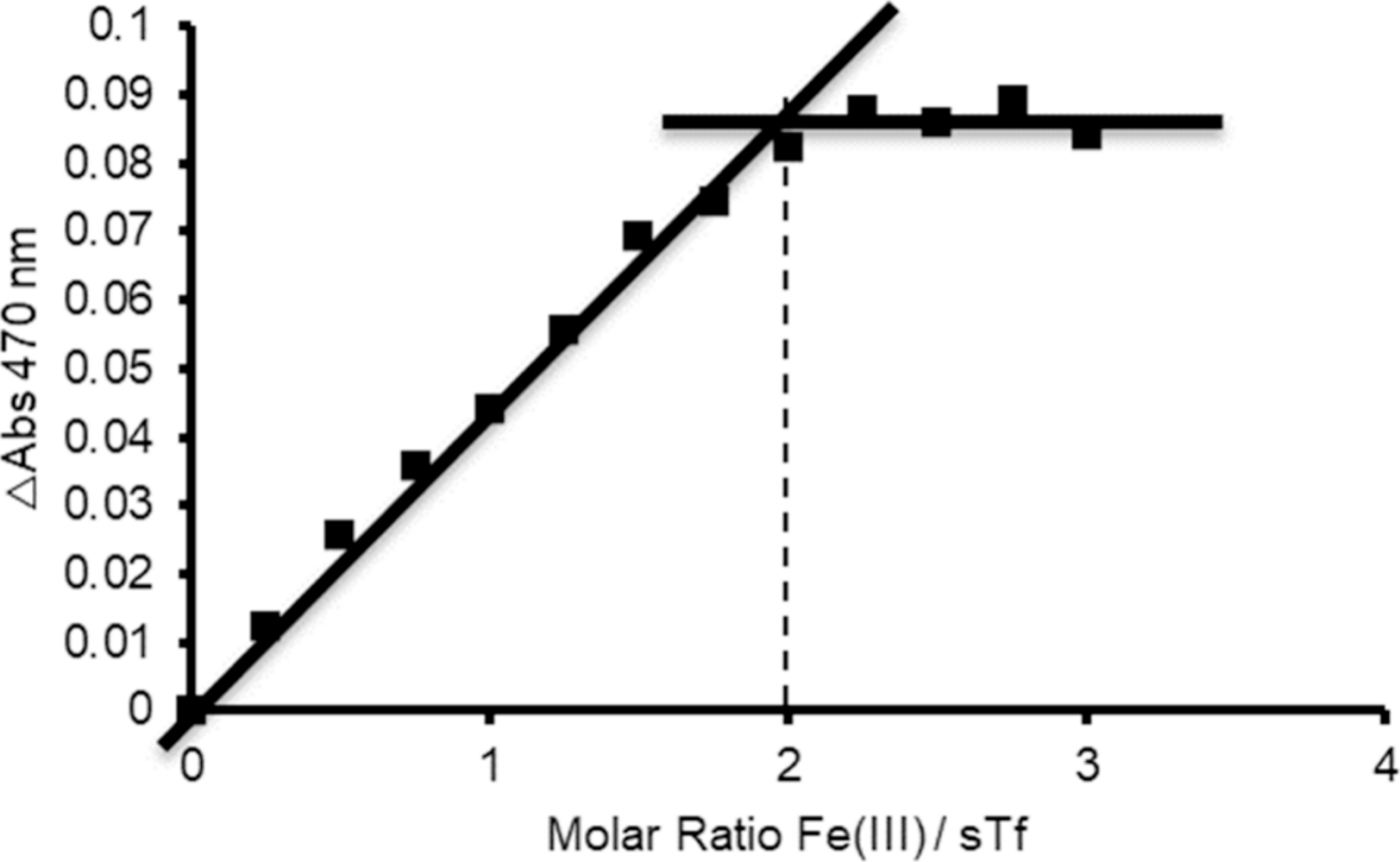
Stoichiometric curve of binding of Fe(III) to sTf. This figure was created using data from one of the student groups.

This undergraduate biochemistry laboratory experience was designed based on applying different strategies that have been developed to assess metal binding affinity and stoichiometry as well as protein stability, using sTf as a case study.^27,31^ In the determination of Fe(III) stoichiometry, this part of the experiment aims to provide a clear understanding of binding using a characteristic spectroscopic signal. The essential information required for this analysis includes the final protein concentration and the source of Fe(III). To determine the ratio between the protein and metal in each solution that students prepare, a table is provided in the manual (Supporting Document B) for the students to complete. To calculate the ratio, the concentration of Fe(III) (in μM) is divided by the concentration of sTf in each solution. In order to visualize the binding of Fe(III) to sTf, the characteristic salmon color formation is observed. This color arises from the LMCT absorbance at 470 nm. The expected extinction coefficient of 5000 M^–1^ cm^–1^ requires the use of a 1 cm path length cuvette. When using a plate reader, the choice of plate and the type of solutions being measured will cause variation of the path length. While a plate reader can be calibrated to obtain a desired extinction coefficient,^42^ for the purpose of this laboratory experience, it is not necessary since the curve is constructed by plotting the absorbance values on the *y* axis and the corresponding ratios on the *x* axis (Figure 5). A line of best fit is drawn through the incremental increases of the presaturation points, and another line is drawn through the final set of absorbance points that show no changes in absorbance due to saturation. The intersection points of these two lines represents the stoichiometric point. A vertical line is drawn from the intersection point down to the axis to determine the precise ratio. By following this approach, students should yield a value near or at 2. It is important to note that the concentrations of metal and protein were selected to be above the affinity constants of Fe(III) to both binding sites (log *K*_C-lobe_ = 22.2 and log *K*_N-lobe_ = 21.3),^1^ such that every addition of metal binds virtually 100% to the protein. Failure to achieve these concentrations results in saturation curves without reaching saturation, with which it is difficult to generate an intersection point.

**Figure 6:**
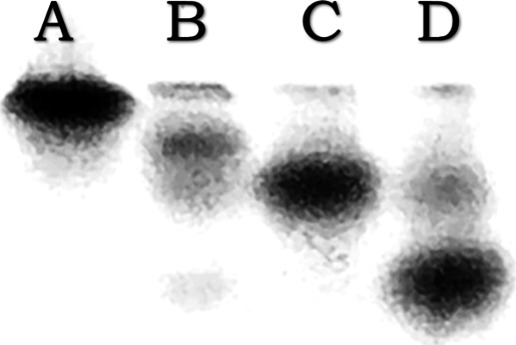
Results of urea-PAGE after 60 min at 280 mV: (A) apo-sTf; (B) FeC-sTf; (C) Fe_N_-sTf; (D) Fe_2_-sTf. This figure was created using data from one of the student groups.

Fe(III) binding by sTf results in different conformational states compared to the open conformation of apo-sTf. The urea-PAGE gel shown here (Figure 6) effectively demonstrates the different stabilities of Fe(III)-bound sTf versus apo-sTf. Well A is apo-sTf solution, which migrates least vertically through the gel because it is the least stable of the protein samples. Well C is Fe_N_-sTf, which due to different intramolecular interactions from lobe closure of the N-site (see the difference in metal binding affinity above) is more stable than Fe_C_-sTf and migrates even further down the gel. Well D is Fe_2_-sTf and, as the most stable protein sample, it migrates furthest down the gel. It is important to note that the choice of the Fe(III) source, Fe(NTA)_2_, and solution conditions was crucial for producing clean results for the formation of the mono-Fe(III) bound sTf samples. One equivalent of Fe(NTA)_2_ exclusively produces Fe_C_-sTf^27,43^ in buffered solution (pH 7.4, 0.1 M NaCl and 20 mM NaHCO_3_). Pure Fe_N_-sTf was generated by an established protocol.^44,45^

To better understand sTf Fe(III) binding and the associated protein structural changes, molecular visualization is essential. PyMOL is freely available and powerful software that allows students to delve deep into understanding the binding sites of proteins. By utilizing PyMOL, students can differentiate between metal-coordinating modalities by examining different crystal structures and identifying the specific amino acids involved in coordination. Furthermore, PyMOL enables students to visualize and explore the interactions at a high resolution, facilitating a better understanding of the important concepts. First, PyMOL helps students measure the distance between the Fe(III) and bound atoms, which relates to the strength of the interaction: the shorter the bond length, the stronger the bond is. Bond lengths between Fe(III) and the coordinating atoms can be compared with standard Fe–O and Fe–N bond lengths. Second, PyMOL aids in comprehending the coordination number of the metal ion and the corresponding coordination geometry. Third, PyMOL allows visualization of secondary interactions such as the binding site of the synergistic anion.^46,47^ Additional secondary interactions like the N-lobe dilysine interaction and the C-lobe triad interaction can be visualized (Supporting Document E). Fourth, PyMOL allows students to observe how differences in Fe(III) coordination via non-carbonate synergistic anions can result in the noncanonical open conformation of bound Fe(III) (Figure 2B).^11,27^ For this laboratory experience, the diferric sTf structure (PDB entry 3QYT) was judiciously selected because it features Fe(III) coordinated in the canonical manner at the C-site and in a noncanonical manner at the N-site.^11^ During the in-lab experience, the students followed an established set of instructions (Supporting Documents D and F) to produce a high-resolution image of Fe(III) bound at the C-site (Figure 2A). The ability to create high-quality protein figures is a useful skill for future publications. As part of the postlab student learning assessment (Supporting Document G), the students construct the structure of Fe(III) bound at the N-site, which contains a bidentate sulfate anion (the carbonate anion is also bound) that prevents coordination of Fe(III) to the aspartate and histidine residues. It is possible to use this structure and other noncanonical structures as examples of the transitory steps by which Fe(III) may bind to or be removed from the protein via chelators.^27^

## Experimental Considerations

- The determination of the concentrations of sTf and Fe(NTA)_2_ is crucial, as it affects the experimental parameter of the ratio.
- Due to the small volume of the solutions that will be prepared, it is recommended to use a low setting on the vortex and centrifuge to ensure homogeneity.
- To minimize sTf denaturation, it is advised to store all of the prepared protein solutions in a refrigerator during the 1 week time between the two lab sessions and to ensure that the protein solutions are left to equilibrate at room temperature by the instructor before the start of the second session.

## Hazards

Instructors and students should wear appropriate personal safety gear at all times. The experiment was carried out in a fume hood. Acrylamide and Fe(NTA)_2_ are hazardous chemicals and carcinogens, and exposure can occur via inhalation (if aerosolized), ingestion, and skin absorption. Caution must be used in working with polyacrylamide, even after gel preparation. Due to electrocution risks, care must be taken when plugging gel boxes into the power supply. Avoiding direct eye contact with the UV–vis laser is needed, as exposure causes acute damage to the cornea and conjunctiva causing pain, light sensitivity, and tearing.

## Fine-Tuning the Lab Experience Based on Student Assessment

**Figure 7:**
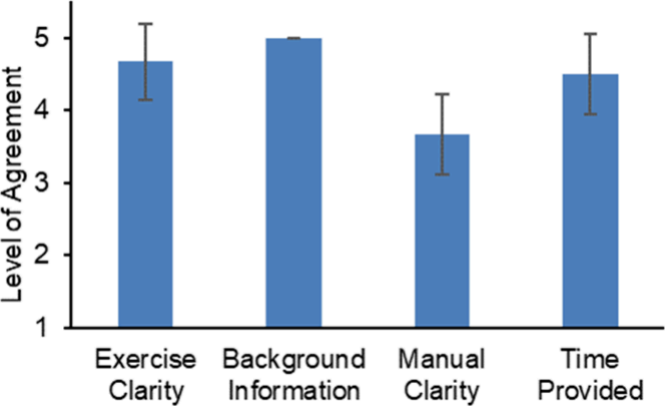
Student feedback (*n* = 12) on the activity design. Students strongly agreed that they received clear instructions and sufficient background information. A neutral to agree response was reported regarding the clarity of the information included in the manual. The students agreed that the time provided was sufficient.

**Figure 8:**
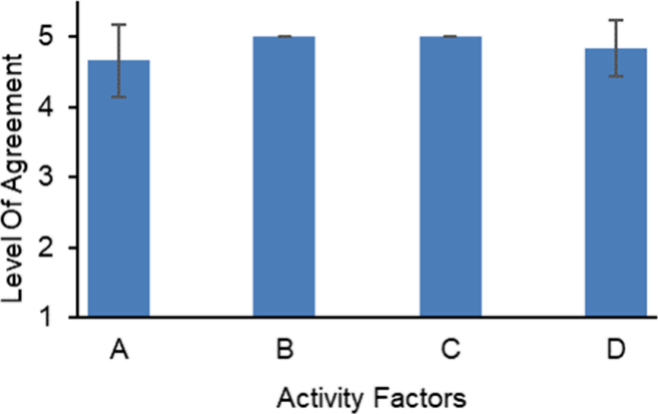
Activity content effectiveness for student development in biochemistry. According to student feedback (*n* = 16), the activity contributed to the development of (A) data interpretation skills and (B) laboratory skills. Additionally, it played a crucial role in nurturing (C) an understanding of the relevance of protein metal coordination in biochemistry studies and (D) improving overall comprehension of chemistry.

**Figure 9:**
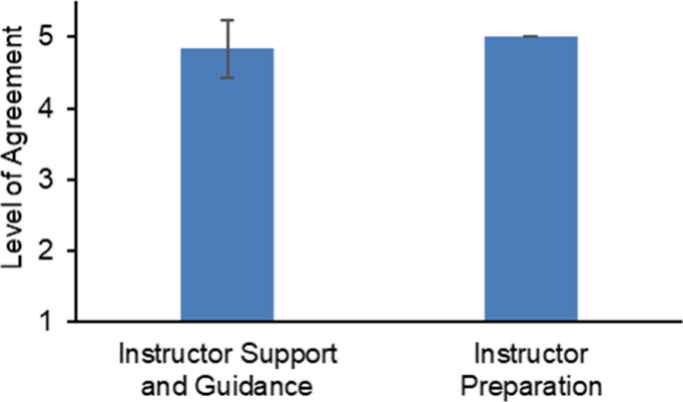
Instructor’s performance on leading the activity. Students (*n* = 16) strongly agreed that the instructors were well-prepared and were satisfied with the support and guidance offered.

**Figure 10:**
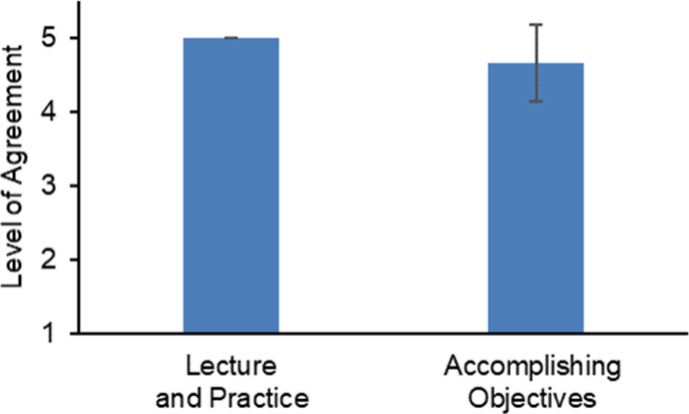
Impact of the activity on students’ academic progress. Students (*n* = 16) were satisfied with the learning experience and the activity’s successful achievement of its objectives.

To fine-tune the lab experience before implementation in a course setting, student assessment was necessary. An IRB-approved protocol was established (IRB: 00000944, protocol number 2223-036). Valuable insight into the effectiveness and educational value of the activity was first obtained by recruiting 12 volunteers (consisting of undergraduate and graduate students) to perform the activity, six volunteers at a time, and then participate in two separate focus group sessions. These sessions allowed volunteers to share their experiences and recommendations regarding how the activity could be improved to enhance its pedagogical efficacy. It is worth noting that the total number of volunteers was modest due to the time commitment involved (8 h for the lab experience and 1 h for the focus group sessions); these trial runs still yielded sufficient feedback to effectively optimize the lab experience and to assess meeting student learning goals. The main objective of these initial trial runs was to optimize the feasibility of integrating the activity into the biochemistry laboratory curriculum offered by our institution’s Department of Chemistry. The eligibility criterion for volunteers consisted of enrollment in an undergraduate biochemistry course or the prior completion of the course. After the laboratory session was completed, the students were provided with the IRB-approved survey consisting of Likert scale questions (Supporting Document H), in which students rated aspects of the activity on a scale of 1 to 5, with 1 indicating “strongly disagree” and 5 meaning “strongly agree” (Supporting Document I and Figures 7–10). It encompassed open-ended qualitative questions to assess individuals’ perceptions regarding the activity. There was also a short open discussion with three questions (Supporting Document H), in which students could freely respond and feed off of each other’s responses. Figure 7 presents the assessment that was useful for fine-tuning the laboratory experience. Two major constructive criticisms were received. Primarily, it was suggested to provide a revised version of the manual with enhanced clarity and the inclusion of images and figures to improve the student’s understanding of the methodology. To address this criticism, a workflow was included (Figures 3 and 4) to guide the students throughout the experiment, including the estimated time for each step. Second, it was recommended to allocate additional time toward the application of the PyMOL program. To address this concern, the PyMOL program is introduced in the first week session.

Following the conclusion of the volunteer trial and the incorporation of the recommendations provided during the focus groups into the activity manual and supporting documentation, the refined version of the experience was implemented in the QUIM4865 course, which is taught by Dr. Raúl Rodríguez Bérrios with the assistance of Jeremy A. Rodríguez Vargas. The actual laboratory experience was facilitated by J.A.B.-R., M.P.O., and A.D.T. To date, this laboratory experience has been practiced by 16 students (excluding the students involved in the trial stage). The responses from these students to the IRB-approved Likert scale questions were useful in assessing the quality of the laboratory experience in student comprehension and drawing connectivity to key biochemistry concepts. Overall, participants expressed strong agreement regarding the activity’s efficacy in facilitating the acquisition of new knowledge in chemistry, the development of laboratory skills, and the understanding of the topic’s significance within the field (Figure 8). Additionally, they strongly agreed that the activity enhanced their data analysis capabilities (Figure 8). With respect to the activity’s design, students strongly agreed that they received clear instructions on how the laboratory exercise would be assessed and that they received sufficient background information. Regarding the instructors’ execution, students strongly agreed that the instructors were well-prepared and offered competent support and guidance (Figure 9). The students also expressed a high level of satisfaction with the overall learning experience and the activity’s successful achievement of its objectives (Figure 10). They found that the activity lecture and execution were of scholarly quality.

Concerning the open-ended questions, the students provided positive feedback regarding the quality and detail of the lectures delivered. One student noted, “I think the experiment is original and creative. The professor’s detailed presentation was amazing.” Furthermore, they also appreciated the exposure to biochemistry topics and biology-oriented laboratory techniques, as their chemistry curriculum primarily focuses on chemistry subjects and procedures. A selection of responses that attest to this sentiment include:

- “I enjoyed the experiment because it covers a lot of experiences related to biochemistry that I did not have the chance to learn or apply during my undergraduate courses.”
- “I liked doing the experiment because we applied biochemical techniques that I had not been able to do since I don’t work in that area. I consider that the experiment is a very complete one.”
- “Participating in this activity helped me understand biochemistry and inorganic concepts in a practical way.”
- “I found the PyMOL program very interesting because it shows a clear visualization of concepts that may not have been explicitly shown in classes.”

Implementing the insightful suggestions from the volunteers helped to optimize the overall learning experience and address specific needs of different students.

## Evaluation of the Learning Objectives

A five-question short-answer prequiz (Supporting Document G**)** covered the general themes discussed in the 1 hour lecture regarding the protein function, structure, and metal binding coordination and relevant Lewis acid–base theory. All students scored perfectly on the prequiz.

Student learning was evaluated after the laboratory experience (Supporting Document G) by having the students analyze their data and compose a lab report in which they addressed specific questions based on the lecture and hands-on component of the experience and discussed pertinent biochemical concepts. All reports were done individually, and the students performed very well, earning grades ≥90%. The student’s greatest comprehension deficiency was comprehending the use of the rapid spin dialyzers for the purpose of washing the protein samples to rid them of unbound metal. The students thought that it was filtering out the metal-free protein, indicating that they did not understand the size-exclusion process. They also made an incorrect comparison between the value of ε collected with the Tecan (which does not have a standard 1 cm path length) with that from the literature, which indicates that there was a misconception about how the path length applies when using the Tecan. We made sure to note these issues in the reports and also discuss them. It is important to reinforce basic concepts of size-exclusion membrane filtration and the path length constant of the Beer’s law equation. In the laboratory practical final exam administered at semester’s end, a few questions about this lab experience are included, specifically concerning the function of sTf in binding and transporting Fe(III), the determination of stoichiometry via a stoichiometric curve, and the assessment of relative stability by urea-PAGE. All of the students responded correctly to these questions, which suggests that the main biochemical concepts were sufficiently understood.

## Conclusions

The two-week protein biochemistry experience effectively reinforces several important themes of the lecture and laboratory courses and enables significant learning domain accomplishments. The key themes include protein biochemistry, protein purification techniques, protein–metal interactions, binding stoichiometry and affinity, and PyMOL application, which enable reinforcement of the learning domains of Content Knowledge, Logical Mathematical Reasoning, and Visualization. The learning domains of Information Literacy and Knowledge Integration are achieved via the lecture and lab manual material provided and the critical thinking postlaboratory report. Student learning is assessed via the postlaboratory report and selected questions in the laboratory course practical exam. Through this experience, students gain a deeper understanding of protein structure and stability influences. They develop proficiency in biochemistry techniques, including gel electrophoresis, spectrophotometric analysis, and PyMOL utilization. They enhance their proficiency in gel electrophoresis, allowing them to interpret protein bands with respect to stability. The spectrophotometric analysis strengthens their competence in understanding absorbance as an indicator of protein saturation. Using PyMOL expands their ability to visualize protein structures and interactions in a 3D format. The experience readily lends itself to use in other formats. The PyMOL portion, for instance, has been used as part of a training workshop on molecular visualization and figure making in the Research Experience for Undergraduates (REU) Program PR-CLIMB (NSF Grant 2050493). Collectively, the experience fosters important practical biochemical skills and teaching opportunities that are useful to students and instructors well beyond the course.

## Supporting Information Available

A: Additional introduction (PDF)
B: Student laboratory manual (PDF)
C: Instructor laboratory manual (PDF)
D: PyMOL download and usage guide (PDF)
E: Representative introductory lecture: “Exploring key features of Fe(III) binding by the blood transporter transferrin” (PDF)
F: Lecture on how to use PyMOL: “Using PyMOL to explore Fe(III) binding by the blood transporter transferrin” (PDF)
G: Student learning assessment (PDF)
H: Student activity assessment (PDF)
I: Visualization of raw data and survey response distribution (PDF)
