# Supplementary material for: Reinforcing Protein Biochemistry: A Two-Week Experiment Studying Iron(III) Binding by the Transferrin Protein through Stoichiometric Determination, Stability Analysis, and Visualization of the Binding Site
Source: J Chem Educ. 2024 Mar 26;101(4):1656–64. doi: 10.1021/acs.jchemed.3c01016 (PMC11033862; doi:10.1021/acs.jchemed.3c01016)
Supplement: Supplementary file 1 — ed3c01016_si_001.pdf [file ed3c01016_si_001.pdf]

Supporting Information

Reinforcing Protein Biochemistry: A Two-Week Experiment Studying Iron(III) Binding by the Transferrin Protein through Stoichiometric Determination, Stability Analysis, and Visualization of the Binding Site

Josué A. Benjamín-Rivera[1,ǂ] Mariela Pérez Otero[2,ǂ] Arthur D. Tinoco[1]*

[1]Department of Chemistry, University of Puerto Rico, Río Piedras Campus, Río Piedras, Puerto Rico 00931, United States.

[2]Department of Biology, University of Puerto Rico, Río Piedras Campus, Río Piedras, Puerto Rico 00931, United States.

ǂ Equal contribution

* Email: atinoco9278@gmail.com

Supporting Information A
Additional Introduction

Table of Content	
	**Page**
Additional Introduction	S2-S3
References	S3-S4

The Fe(III)-sTf TfR1 mediated endocytosis.

Each sTf molecule binds two Fe(III) ions. The protein is the major molecular pathway for Fe(III) delivery to cells throughout the body via the metal uptake transferrin receptor 1 (TfR1)-mediated endocytosis (**Figure 1**).[1 2 3 4] This pathway is highly pH-dependent.[5] At the pH of blood (pH 7.4), Fe-bound sTf (Fe_2-sTf) has a very high affinity for the membrane-bound receptor whereas the metal-free protein (apo-sTf) does not. Two molecules of Fe_2-sTf can bind to TfR1. This binding interaction triggers the formation of an endosome, which becomes clathrin protein coated to protect the endosome from proteolytic destruction. A V-ATPase pump decreases the endosome pH from 7.4 to 5.5. A combination of the pH drop and intracellular chelation (possibly citrate), strips Fe(III) from sTf. The metal ion is then reduced to Fe(II) by the Steap3 reductase, enabling the Fe(II) to be released from the endosome via the divalent metal transporter 1 (DMT1).[5] The apo-sTf remains bound to TfR1 given its significant affinity for the receptor at pH 5.5. It returns to the membrane and reenters the bloodstream to be recycled for additional rounds of Fe(III) uptake. Once Fe(II) is released from the endosome, it enters into a transient labile iron pool within cytosol and then is ultimately trafficked for storage within the ferritin protein or for functional use following molecular binding.[5] The majority of the Fe is found in the hemoglobin protein, which is responsible for binding and transporting O_2, and allowing humans to breathe.

An important difference in the second coordination sphere of the sTf Fe(III) binding site.

Crystallography reveals important differences that exist in the second coordination sphere of the metal binding sites; the sphere that influences the stability of the primary coordination sphere but does not involve any direct atom coordination to the metal ion. The two metal binding sites possess similar but distinct nearby noncovalent interactions that help stabilize the Fe(III) coordination. The N-site has a pH-sensitive dilysine interaction involving a H-bond between the two lysines (Lys206

and Lys296)[6] whereas the C-site has a pH-sensitive triad interaction[7] amongst Lys534, Arg632, and Asp634, which involves H-bonding and electrostatic attraction between the positively and negatively charged amino acids. These different secondary sphere interactions contribute to the difference in the relative stability of Fe(III) bound by the N and C-lobes.[8]

References

(1) Bonvin, G.; Bobst, C. E.; Kaltashov, I. A. Interaction of transferrin with non-cognate metals studied by native electrospray ionization mass spectrometry. *Int. J. Mass Spectrom.* **2017**, *420* (Supplement C), 74-82. DOI: https://doi.org/10.1016/j.ijms.2017.01.014.

(2) Breuer, W.; Epsztejn, S.; Cabantchik, Z. I. Iron Acquired from Transferrin by K562 Cells Is Delivered into a Cytoplasmic Pool of Chelatable Iron(II). *J. Biol. Chem.* **1995**, *270* (41), 24209-24215. DOI: 10.1074/jbc.270.41.24209.

(3) Ponka, P.; Beaumont, C.; Richardson, D. R. Function and regulation of transferrin and ferritin. *Semin. Hematol.* **1998**, *35* (1), 35-54.

(4) Yang, N.; Zhang, H. M.; Wang, M. J.; Hao, Q.; Sun, H. Z. Iron and bismuth bound human serum transferrin reveals a partially-opened conformation in the N-lobe. *Sci. Rep.* **2012**, *2*, Article. DOI: 10.1038/srep00999.

(5) Steere, A. N.; Byrne, S. L.; Chasteen, N. D.; Mason, A. B. Kinetics of iron release from transferrin bound to the transferrin receptor at endosomal pH. *Biochim. Biophys. Acta* **2012**, *1820* (3), 326-333. DOI: 10.1016/j.bbagen.2011.06.003.

(6) Dewan, J. C.; Mikami, B.; Hirose, M.; Sacchettini, J. C. Structural evidence for a pH-sensitive dilysine trigger in the hen ovotransferrin N-lobe: implications for transferrin iron release. *Biochemistry* **1993**, *32* (45), 11963-11968. DOI: 10.1021/bi00096a004. Peterson, N. A.; Arcus, V. L.; Anderson, B. F.; Tweedie, J. W.; Jameson, G. B.; Baker, E. N. "Dilysine trigger" in transferrins probed by mutagenesis of lactoferrin: crystal structures of the R210G, R210E, and R210L mutants of human lactoferrin. *Biochemistry* **2002**, *41* (48), 14167-14175. DOI: 10.1021/bi020443a. Gumerov, D. R.; Kaltashov, I. A. Dynamics of iron release from transferrin N-lobe studied by electrospray ionization mass spectrometry. *Anal. Chem.* **2001**, *73* (11), 2565-2570. DOI: 10.1021/ac0015164. He, Q. Y.; Mason, A. B.; Tam, B. M.; MacGillivray, R. T. A.; Woodworth, R. C. Dual role of Lys206-Lys296 interaction in human transferrin N-lobe: Iron-release trigger and anion-binding site. *Biochemistry* **1999**, *38* (30), 9704-9711. DOI: 10.1021/bi990134t.

(7) Halbrooks, P. J.; Giannetti, A. M.; Klein, J. S.; Björkman, P. J.; Larouche, J. R.; Smith, V. C.; MacGillivray, R. T. A.; Everse, S. J.; Mason, A. B. Composition of pH-Sensitive Triad in C-Lobe of Human Serum Transferrin. Comparison to Sequences of Ovotransferrin and Lactoferrin Provides Insight into Functional Differences in Iron Release. *Biochemistry* **2005**, *44* (47), 15451-15460. DOI: 10.1021/bi0518693.

(8) Lin, L. N.; Mason, A. B.; Woodworth, R. C.; Brandts, J. F. Calorimetric studies of serum transferrin and ovotransferrin-Estimates of domain interactions, and study of the kinetic complexities of ferric ion-binding. *Biochemistry* **1994**, *33* (7), 1881-1888, Article. DOI:

10.1021/bi00173a035. Evans, R. W.; Williams, J. The electrophoresis of transferrins in urea/polyacrylamide gels. *Biochem. J.* **1980**, *189* (3), 541-546. Byrne, S. L.; Mason, A. B. Human serum transferrin: a tale of two lobes. Urea gel and steady state fluorescence analysis of recombinant transferrins as a function of pH, time, and the soluble portion of the transferrin receptor. *J. Biol. Inorg. Chem.* **2009**, *14* (5), 771-781, Article. DOI: 10.1007/s00775-009-0491-y.
