# Supplementary material for: Reinforcing Protein Biochemistry: A Two-Week Experiment Studying Iron(III) Binding by the Transferrin Protein through Stoichiometric Determination, Stability Analysis, and Visualization of the Binding Site
Source: J Chem Educ. 2024 Mar 26;101(4):1656–64. doi: 10.1021/acs.jchemed.3c01016 (PMC11033862; doi:10.1021/acs.jchemed.3c01016)
Supplement: Supplementary file 3 — ed3c01016_si_003.pdf [file ed3c01016_si_003.pdf]

Supporting Information

Reinforcing Protein Biochemistry: A Two-Week Experiment Studying Iron(III) Binding by the Transferrin Protein through Stoichiometric Determination, Stability Analysis, and Visualization of the Binding Site

Josué A. Benjamín-Rivera[1,ǂ] Mariela Pérez Otero[2,ǂ] Arthur D. Tinoco[1]*

[1]Department of Chemistry, University of Puerto Rico, Río Piedras Campus, Río Piedras, Puerto Rico 00931, United States.

[2]Department of Biology, University of Puerto Rico, Río Piedras Campus, Río Piedras, Puerto Rico 00931, United States.

[ǂ] Equal contribution

* Email: atinoco9278@gmail.com

Supporting Information C

Instructor Laboratory Manual

Table of Content	
	**Page**
I. Laboratory Goals	S2-S3
II. Materials and Equipment	S3-S4
III. Instructions	S4-S9

I. LABORATORY GOALS

WEEK 1

First Goal: Learn about how sTf binds Fe(III) and how this binding interaction is important for maintaining iron homeostasis.

Second Goal: Perform sample preparations for different metal binding experiments.

Third Goal: Install the PyMOL application.

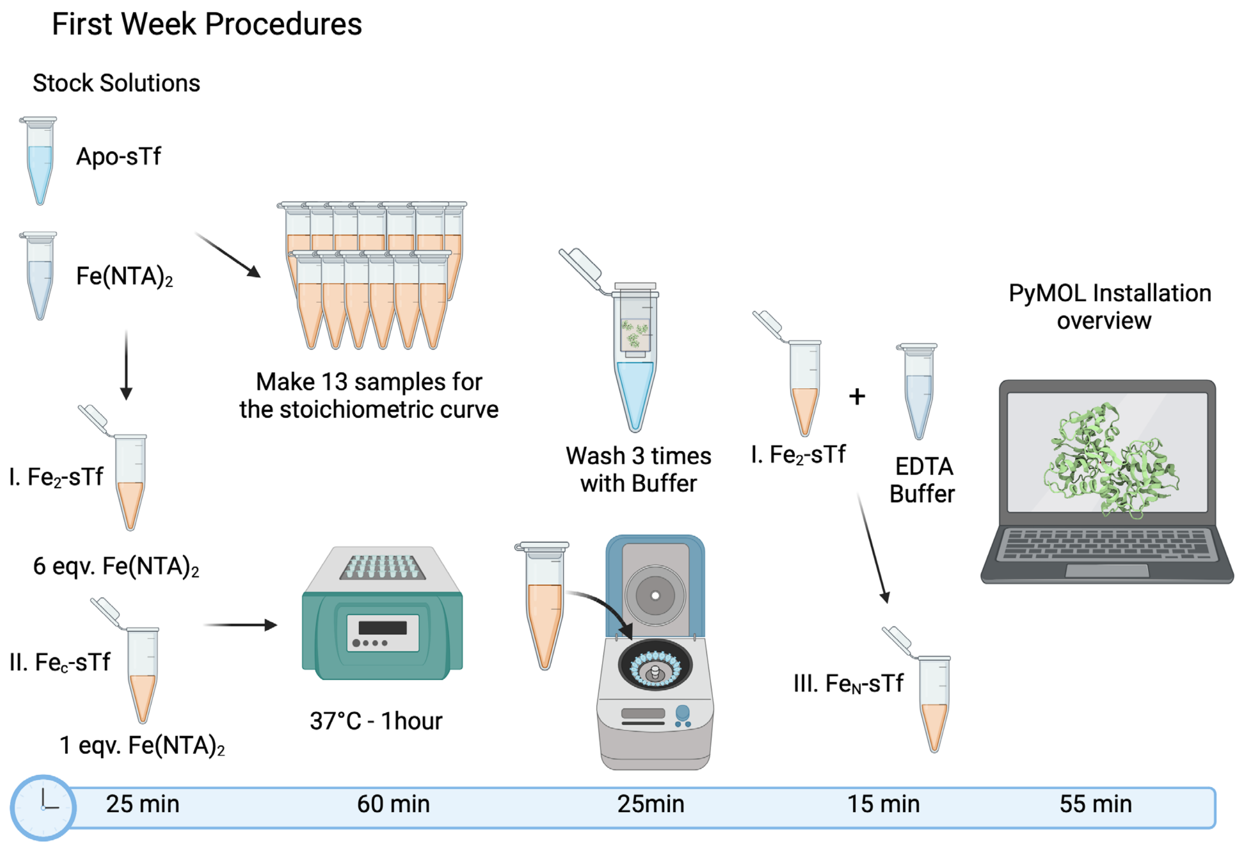

WEEK 2

Fourth Goal: Construct a stoichiometric curve to determine the number of Fe(III) ions that bind to the protein and the molar coefficient of the metal-protein complex.

Fifth Goal: Run a Urea Gel to evaluate sTf stability in the presence and absence of Fe(III) ions.

Sixth Goal: Familiarize yourself with the PyMOL application and learn the different methods and add-ons you can use to generate a crystallized protein structure.

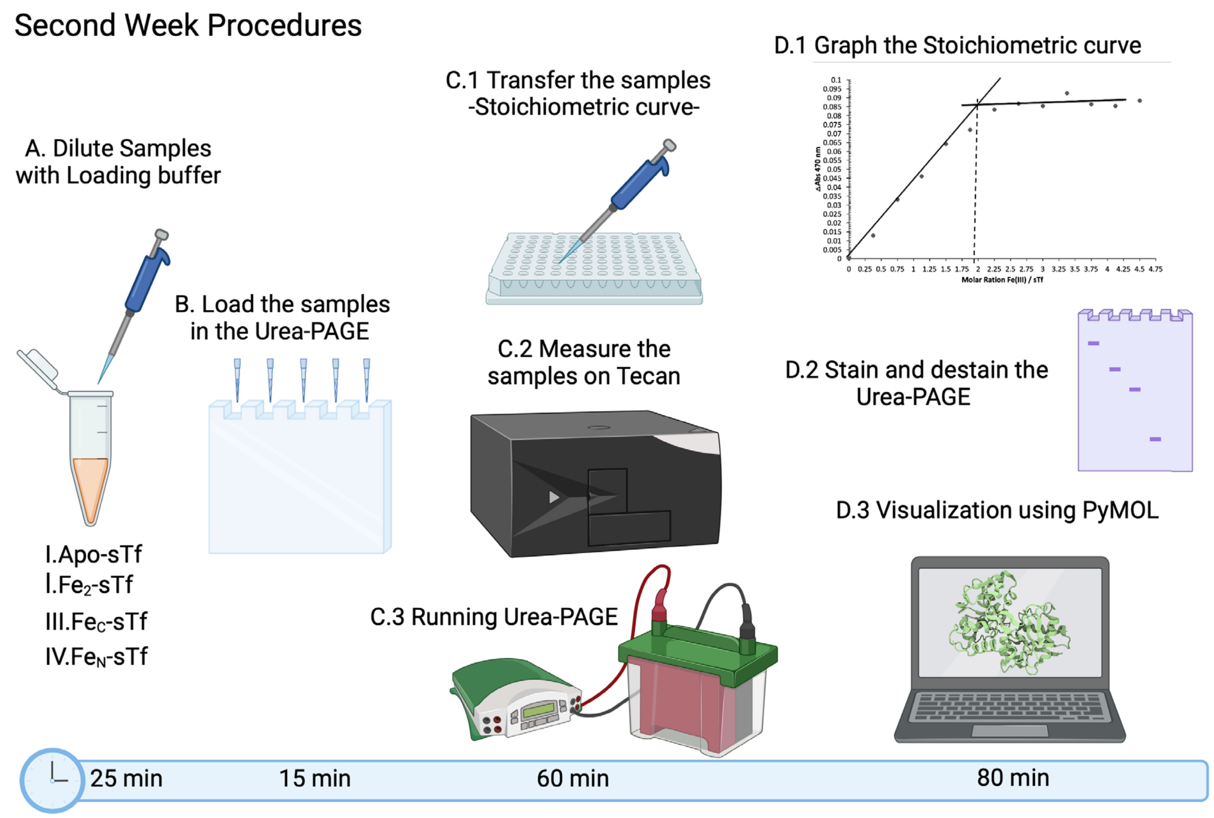

II. MATERIALS AND EQUIPMENT

aposTf stock solution 16 mg/mL (volume 900 µL each group)
*Molecular Weight of sTf is 80 kDa

0.25 M Tris buffer pH 7.4 (150 mM NaCl, 20 mM $NaHCO_3$)

1 mM $Fe(NTA)_2$

200 mM EDTA (in a pH 7.4, 0.1 M HEPES buffer that also contains 2.67 M $NaClO_4$)

Urea gel loading buffer

Urea gel staining solution

Urea gel destaining solution

1.5 mL and 0.5 mL eppendorf tubes

An eppendorf tube rack

Centrifuge for eppendorf tubes (14,000 rpm capability)

Centricon filter (3 kDa molecular weight cut-off)

Plate Reader (Tecan)

96-well plate

UV-VIS spectrophotometer

1 mL cuvette

III. INSTRUCTIONS

WEEK 1

For instructors: (To do before the laboratory experience)

A. Preparation of $Fe(NTA)_2$

10 mL of 1 mM Iron bisnitrilotriacetate ($Fe(NTA)_2$) was prepared by adding 10 µL of the 1 M $FeCl_3$ (aq) and 320 µL of 62.5 mM nitrilotriacetic acid (NTA). It is pH adjusted to pH 4.0.

B. Preparation of EDTA

First make 100 mL of 0.2 M HEPES Buffer with 5.4 M $NaClO_4$ at pH 7.4. Then mix 50 mL of the solution with 50 mL of 200 mM EDTA in water to obtain the 100 mM EDTA buffer with 0.1 HEPES and 2.7 $NaClO_4$ at pH 7.4.

C. Coomassie blue solution

Prepare the Urea gel staining solution containing 0.1% Coomassie R-250 in 40% ethanol, 10% acetic acid and 50.1% water.

D. TBE 1X

Prepare a 1X TBE solution by diluting 100 mL 10X TBE with 900 mL water.

E. TBE 5X

Prepare a 5X TBE solution by diluting 500 mL 10X TBE with 500 mL water.

F. 10% APS (Ammonium persulphate solution)

Dissolve 1g APS in 10 mL water (transfer 220 uL in eppies tubes and store at -20°C).

G. Destain solution

Prepare a Urea gel destaining solution containing 10% (v/v) ethanol and 7.5% (v/v) acetic acid in water.

For students:

Preparation of Fe(III)-bound sTf solutions for the Urea-PAGE analysis.

A. Preparation of different Fe(III) bound sTf samples.

1. Fe_2-sTf

   A 200 µL volume sample of **Fe_2-sTf** will be prepared by mixing 100 µL of **200 µM apo sTf,** 80 µL of 1mM **$Fe(NTA)_2$,** and 20 µL of **buffer.** Gently pipette up and down to mix the solution. Let incubate for 1 h at 37°C. Wash the sample with the 0.25 M Tris Buffer using the centricon filter. Lastly, concentrate the sample to a final volume of 200 µL.

2. Fe_c-sTf

   A 200 µL volume sample of **Fe_c-sTf** will be prepared by mixing 100 µL of **200 µM apo sTf**, 20 µL of **1mM $Fe(NTA)_2$,** and 80 µL of **buffer**. Gently pipette up and down to mix the solution. Let incubate for 1 h at 37°C. Wash the sample with the 0.25 M Tris Buffer using the centricon filter. Lastly, concentrate the sample to a final volume of 200 µL.

3. Fe_N-sTf

   A 100 µL volume sample of **Fe_N-sTf** will be prepared by mixing 50 µL of **200 µM Fe_2-sTf** (prepared in week 1), and 50 µL of **200 mM EDTA**. Gently pipette up and down to mix the solution. Let the reaction run for 15 min.

While you let the samples incubate, proceed to part C, and prepare the following solutions.

B. Dialysis of the samples

Background information:

The centricon contains a membrane that aids in filtering the sample through the membrane when subjected to centrifugal force, which propels solvents and small solutes through the membrane and into the filtrate vial. Larger macromolecules, like proteins, are retained

above the membrane in the sample reservoir. As the volume of the sample decreases, the concentration of these larger solutes increases.

Procedure:

After preparing the samples presented above you will need to wash samples Fe_2-sTf and Fe_c-sTf by spin dialysis, using a Centricon tube. You will transfer all the sample to the centricon tube. Then add 200 uL of 0.25 M Tris buffer to the tube and proceed to centrifuge it at 14,000 rpm for 3 minutes to perform the wash. You will do this process 3 times. To retrieve them, the sample is moved to the retentate vial by placing it above the reservoir, flipping the device, and briefly spinning it. Lastly, concentrate the sample to a final volume of 200 µL.

Homework to prepare for Week 2:

Determine the final concentration of sTf in each solution using the equation:

$$M_1V_1 = M_2V_2$$

Samples	Volume (µL) of pipetted protein (V_1)	Final volume (µL) of solution (V_2)	Initial concentration (µM) of sTf (M_1)	Final concentration (µM) of sTf µM (M_2)
Fe_2-sTf	100	200	200	100
Fe_c-sTf	100	200	200	100
Fe_N-sTf	50	200	200	50

<u>Preparation of Fe(III)-bound sTf solutions to determine Fe binding stoichiometry</u>

In this experiment, different equivalents of Fe(III) will be added to a set concentration of sTf as indicated in Table 1. The solutions will be prepared in 0.5 mL eppies and left to equilibrate until week 2. Note any color changes due to the addition of Fe(III).

Total Volume = 100 µL

[sTf] stock = 200 µM

Note: Add the protein first, then the $Fe(NTA)_2$, and lastly 0.25 M Tris Buffer.

Table 1: Preparation of Fe(III)-bound sTf solutions

Sample #	Volume of Apo sTf Stock (µL)	Volume of $Fe(NTA)_2$ Stock (µL)	Volume of Tris Buffer (µL)	Change in color?	Molar ratio of Fe(III)
1	50	0	50	No color	0

2	50	2.5	47.5	Yes, lite salmon	0.25
3	50	5	45	Yes, lite salmon	0.5
4	50	7.5	42.5	Yes, lite salmon	0.75
5	50	10	40	Yes, salmon	1.00
6	50	12.5	37.5	Yes, salmon	1.25
7	50	15	35	Yes, salmon	1.5
8	50	17.5	32.5	Yes, salmon	1.75
9	50	20	30	Yes, salmon	2.00
10	50	22.5	27.5	Yes, salmon	2.25
11	50	25	25	Yes, salmon	2.5
12	50	27.5	22.5	Yes, salmon	2.75
13	50	30	20	Yes, salmon	3.00

In week 2 you will add these samples into a 96-well plate to measure their absorbance.

Note: To determine the molar ratio of Fe(III), you need to follow these steps:

1. Calculate the final concentration of sTf and Fe(III) in each solution by using the initial concentrations and the dilution factor.
2. Divide the concentration of Fe(III) in each solution (in µM) by the concentration of sTf in the same solution (in µM).
3. Repeat step 2 for all the solutions and record the molar ratio of Fe(III) for each solution.

WEEK 2

Constructing the stoichiometric curve for the Fe(III) titration

Fe(III) binding to sTf produces a characteristic pink color. This is due to a ligand to metal charge transfer (LMCT) absorbance at 470 nm because of the tyrosine binding of Fe(III). This absorbance of the Fe(III) saturated protein (Fe_2-sTf) has an extinction coefficient of 2,500 $M^{-1}cm^{-1}$ based on Fe(III) concentration. The extinction coefficient is 5,000 $M^{-1}cm^{-1}$ based on protein concentration.

Measuring the Absorbance of the samples

Transfer the Fe(III)-bound sTf solutions to a 96-well plate and measure their absorbance at **470 nm** and **800 nm** (as a zero absorbance control) using the Plate Reader. Transfer **90 µL** of the samples in each well. It is important that the transfer be complete as possible because the absorbance measurements are volume dependent. Avoid bubbles. Correct the absorbance at

470 nm by subtracting the absorbance at **800 nm** for each well. Prepare a stoichiometric curve by plotting the corrected absorbance at **470 nm vs the Fe/Tf mole ratio.**

Use the picture of the 96-well plate to identify where your samples are located.

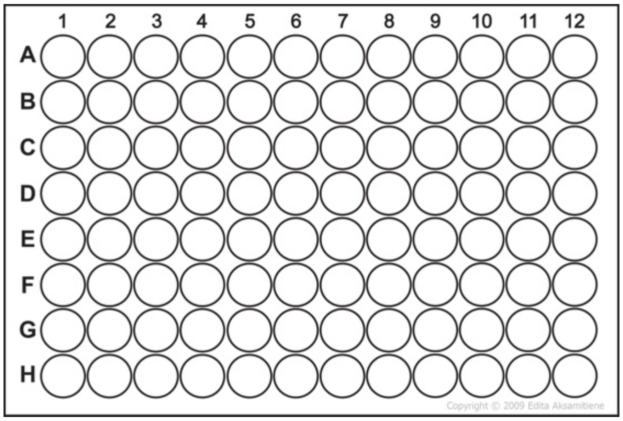

<u>Comparing the stability of apo sTf and the different Fe(III) bound sTf samples via Urea - PAGE analysis</u>

For instructor: (To do before laboratory experience)

A. <u>Preparation of Urea gel</u>

1. Separating gel preparation:

   First, add 2.25 mL of 40% acrylamide, 6.3 g of urea (7 M), and 3.0 mL of TBE buffer (5X). This solution is to be dissolved in a temperature of 60 °C. Once finished with both gels, add 150 µL of 10% APS and 6 µL of TEMED. Complete to a volume of 15 mL with water.

2. Stacking gel preparation:

   Repeat the exact same process as indicated above but with 0.5 mL 40% acrylamide, 2.1 g of urea (7 M), 1.0 mL TBE buffer (5X), 50 µL 10% APS and 4 µL TEMED. Then complete to a final volume of 5 mL with water

3. Gel preparation:

   Add separating gel (3 mL) and level with 200 µL of ethanol. Leave to solidify for approximately 5 minutes. Remove the ethanol, add the stacking gel (1 ml) next, and put the gel releasers in. Once the gel is ready, transfer it to the hand-cast gel, fill in the inside of the chamber with 1X TBE buffer, and outside the chamber with the buffer up to the halfway point. Checking for leakage from inside the chamber after filling the hand-cast gel. Once the channels are ready, add 15 µL of your samples with a running dye. Place electrodes with a current of 280 volts for 60 minutes. Afterward, take out

the gel and place it a Tupperware and wash it with water. Remove the water to add the Coomassie blue solution. Heat the gel in the microwave for 30 seconds to accelerate staining. Remove the Coomassie blue solution and rinse with water. Next add the destaining solution and leave overnight while mixing with kimwipes on one side (the kimwipes will help to trap the Coomassie blue). Then, it is microwaved once again for 30 seconds to be analyzed using a camera to take an image of the gel.

For students:

1. Dilute the **apo sTf, Fe_2-sTf, Fe_C-sTf, and Fe_N-sTf** (samples) from their respective concentrations to **25 µM sTf** in 0.25 M Tris buffer for a final volume of 50 µL.

   - **Example:** Pipette 6.25 µL of the stock of 200 µM sTf into an eppy and add 43.75 µL of 0.25 M Tris Buffer.

2. Dilute the **apo sTf, Fe_2-sTf, Fe_C-sTf, and Fe_N-sTf** samples of **25 µM** with the Urea Gel loading buffer to get a concentration of **7 µM** in a volume of **20 µL**

   - **Example:** Pipette 5.6 µL of the stock of 25 µM sTf in an eppy and add 14.4 µL of Loading Buffer. *Final volume - 20 µL*

3. Load 15 µL of each sample into the different wells of the gel.

4. Run the gel for 1 hour following instructor's guidelines.

5. Stain and destain the gel.

6. Take an image of the destained gel.

<u>Using PyMOL to visualize Fe(III) coordination by serum transferrin (sTf)</u>

In this part of the laboratory experience, students will study the structure of Fe(III) bound sTf using the protein visualizing program (PyMOL). PyMOL is an open source molecular visualization system that produces high-quality 3D images of proteins, nucleic acids, small molecules, electron densities, surfaces, and trajectories. It can also be used to make short videos to demonstrate molecular dynamics. You will learn how to create a high quality structure of Fe(III) bound sTf with PyMOL using a published structure. We will download this structure from The Research Collaboratory for Structural Bioinformatics Protein Data Bank (RCSB PDB) is the U.S. data center for the global PDB archive of 3D structural data for large biomolecules (proteins, DNA, and RNA). You will have to download structure **3QYT** from the RCSB PDB site. In this structure, Fe(III) is bound in a closed conformation in the C-lobe binding site and in an open conformation in the N-lobe binding site. To complete this part of the experience you must download the PyMOL program and bring your laptop to the laboratory session.
