# Supplementary material for: Reinforcing Protein Biochemistry: A Two-Week Experiment Studying Iron(III) Binding by the Transferrin Protein through Stoichiometric Determination, Stability Analysis, and Visualization of the Binding Site
Source: J Chem Educ. 2024 Mar 26;101(4):1656–64. doi: 10.1021/acs.jchemed.3c01016 (PMC11033862; doi:10.1021/acs.jchemed.3c01016)
Supplement: Supplementary file 4 — ed3c01016_si_004.pdf [file ed3c01016_si_004.pdf]

Supporting Information

Reinforcing Protein Biochemistry: A Two-Week Experiment Studying Iron(III) Binding by the Transferrin Protein through Stoichiometric Determination, Stability Analysis, and Visualization of the Binding Site

Josué A. Benjamín-Rivera[1,ǂ] Mariela Pérez Otero[2,ǂ] Arthur D. Tinoco[1]*

[1]Department of Chemistry, University of Puerto Rico, Río Piedras Campus, Río Piedras, Puerto Rico 00931, United States.

[2]Department of Biology, University of Puerto Rico, Río Piedras Campus, Río Piedras, Puerto Rico 00931, United States.

[ǂ] Equal contribution

* Email: atinoco9278@gmail.com

Supporting Information D

PyMOL Download and Usage Guide

Table of Content

	Page
I. Instructions to download the pymol program	S2
II. Instructions on how to use the pymol program	S3-S5
III. High-resolution image of the Fe(III) C-lobe binding site of STf	S6-S10

Note: For best use of the PyMOL program, you must use a three-button external mouse.

I. <u>Instructions to download the PyMOL program</u>

A. <u>Requesting the Education License:</u>

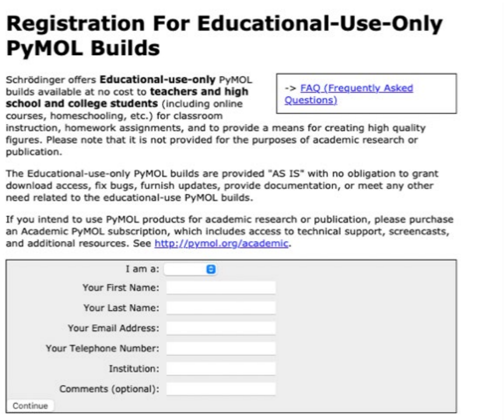

1. Visit https://pymol.org/edu/
2. Fill out the form to obtain a username and password.
3. You will receive an email with further instructions for the next steps.

B. <u>To download PyMOL:</u>

4. Use the following URL: https://pymol.org/ep
5. After logging in, you will need to download the license file.
6. Visit the download page (the link below license file) to download the PyMOL version compatible with your computer.
7. Install the downloaded file.
8. After installation is complete, you need to activate the program using the license obtained from your previous download.

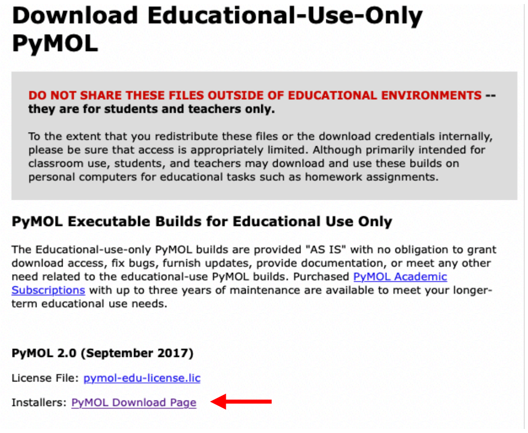

Note: The workshop uses version 2.3; using a different version may result in different commands.

II. Instructions on how to use PyMOL program

A. Accessing Protein Data:

1. Visit https://www.rcsb.org/ to access the Research Collaboratory for Structural Bioinformatics Protein Data Bank (RCSB PDB).
2. Access and download the structure **3QYT** from the RCSB PDB.

B. Opening and Navigating PDB Files:

1. Use PyMOL's upper control panel, command line, and object menu panel to navigate.
2. Click File and select “Get PBD”.
3. Write 3QYT in the PBD ID line.
4. Chain name is to select only one chain if the PDB has multiple chains.

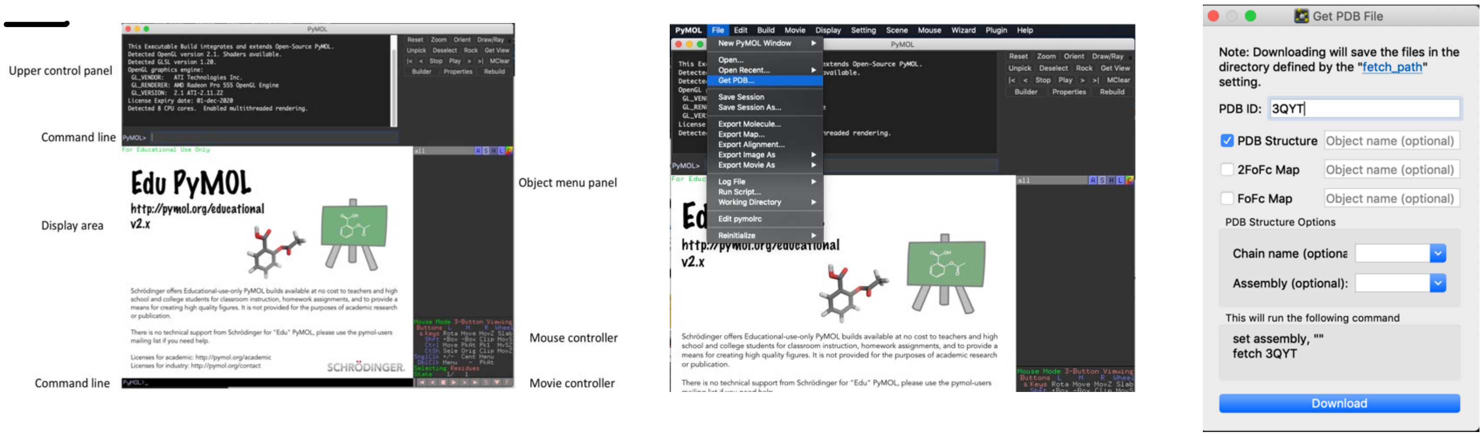

C. Visualizing all the amino acids:

1. Click Display→ Sequence Mode → Residue Names
2. Click Display→ Sequence

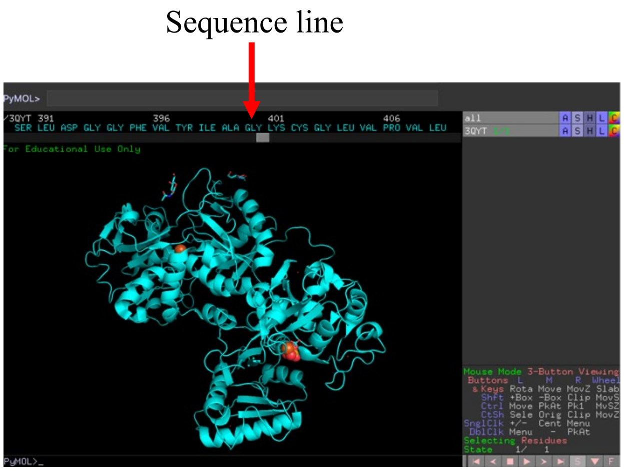

Note: The sequence line will help identify any component in the PDB, like amino acids, small molecules, and metal ions.

D. Illustrating amino acids and synergistic anions bound to Fe(III) within the C-lobe of STf:

1. In the object menu panel Click the **A (Action command)**
2. Click **Preset: Ligands**

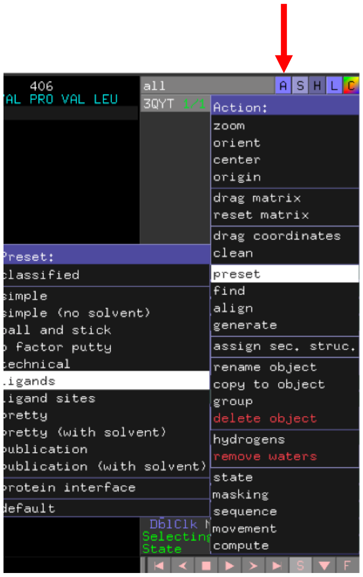

Note: This action reveals the two metal binding sites and the interactions with the metal ion. The rest of the protein will appear as a ribbon.

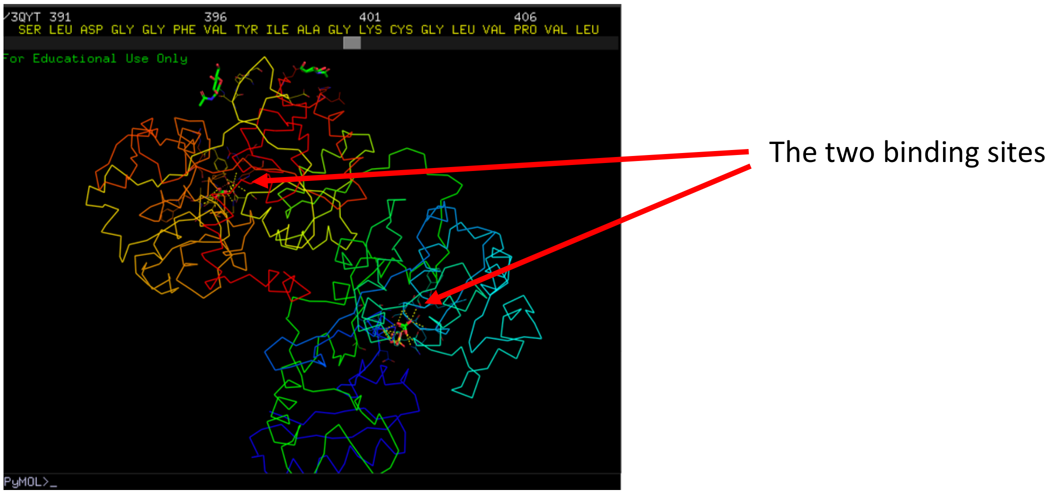

E. Illustrating only the interactions within the metal binding site:

1. In the object menu panel Click the **H (Hide command)**
2. Click **ribbon**
3. Zoom into one of the binding sites to determine the coordination details of Fe(III).

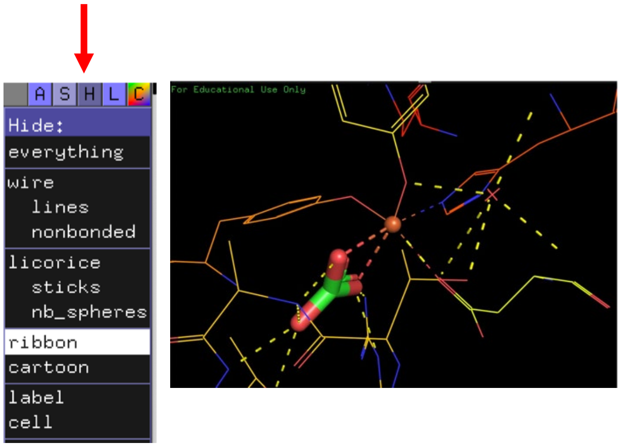

F. Identifying the amino acids bound to Fe(III):

1. Click on each amino acid that interacts directly with Fe(III)
2. Use the sequence line for reference.

Example: The molecule that is clicked is the one in orange highlight.

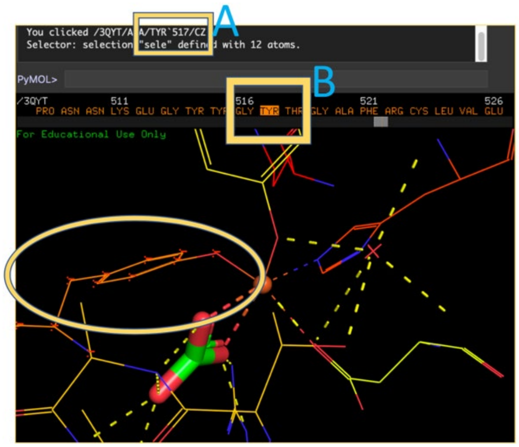

3. To see the identification:

A. Note the text that is displayed in the upper control panel

B. Look at the amino acid marked in the sequence line.

What is the identification of the amino acid?

TYR 517

G. Preparing a Table:

1. Create a table listing bound amino acids and synergistic anions for both C-lobe and N-lobe sites.
2. Review the lecture slides for an example.

III. High-resolution image of the Fe(III) C-lobe binding site of STf

A. To begin:

1. Close and reopen the PyMOL program.
2. Download the protein following the previously provided instructions.

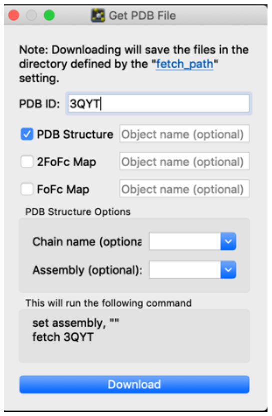

B. Selecting amino acids:

1. Select **His585** from the sequence line.
2. Change the name (sele) in the object menu panel
3. Name using the 3-letter code and protein position of **His585**
4. Unselect His585
5. Follow the same protocol and select and name the rest of the metal binding site, including carbonate and Fe.

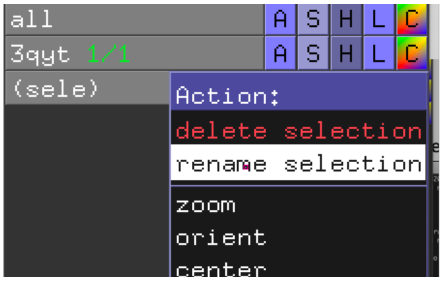

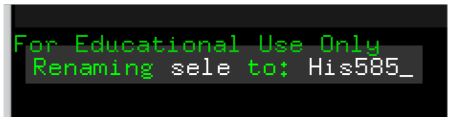

C. Improving Visualization:

1. Type in the command line:
   a) set cartoon_side_chain_helper, 1

      -This eliminates the backbone of the amino acids

   b) remove hydro

      -This removes the hydrogens

   c) set sphere_scale, 0.25, (all)

      -This reduces the size of the sphere

2. Change background by clicking Display → Background → white
3. Remove the valences by clicking Display → uncheck 'Show Valences'
4. Set protein transparency to **gray 90** to make protein transparent.

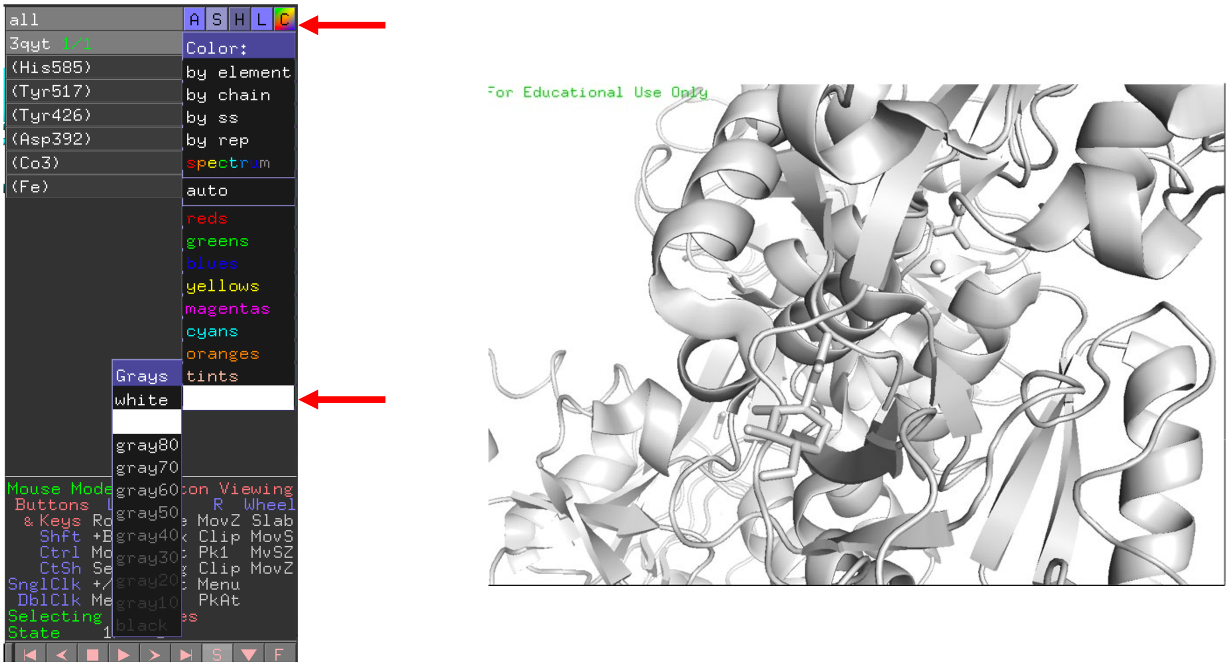

5. Modify Amino Acid appearance by selecting His585 and changing its shape and color.

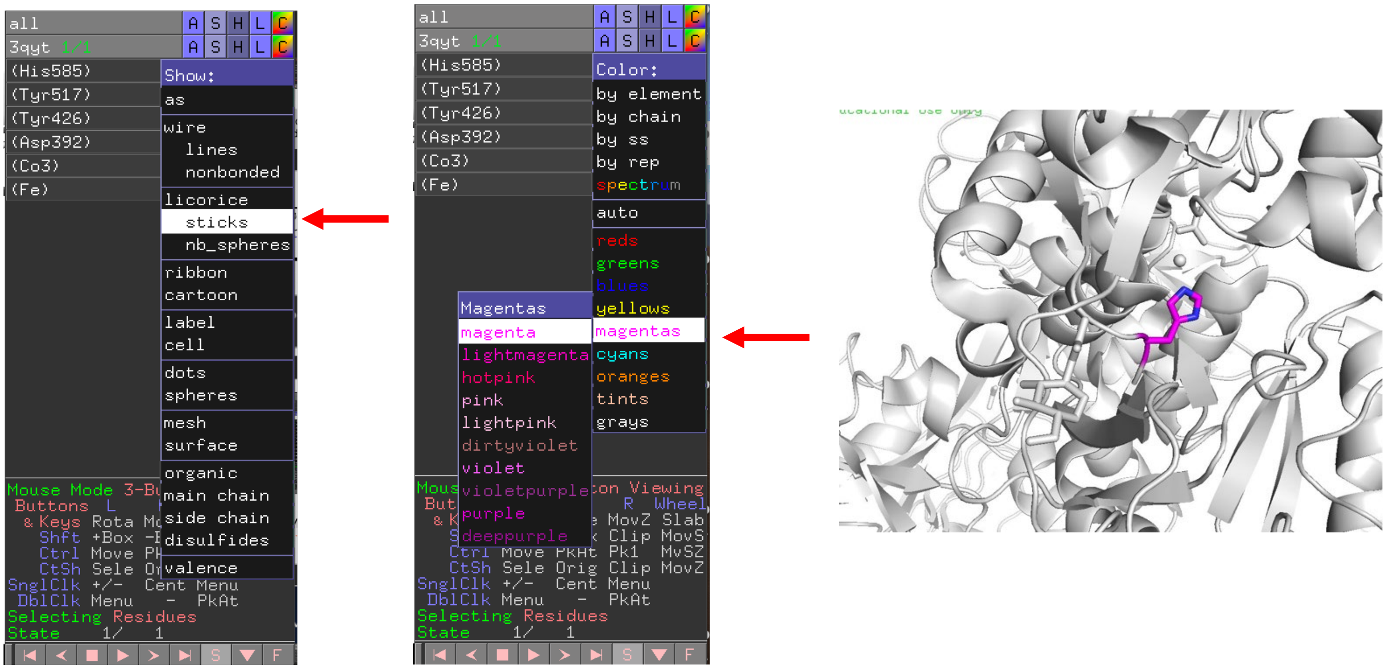

6. Repeat the process for other parts of the metal binding site.

D. Labeling the metal binding site:

1. Begin by switching to 3-button editing mode.
2. Click on the Fe atom and label it.
3. Repeat the labeling process for the rest of the binding site.

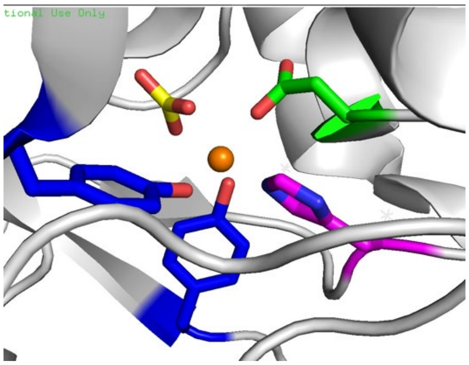

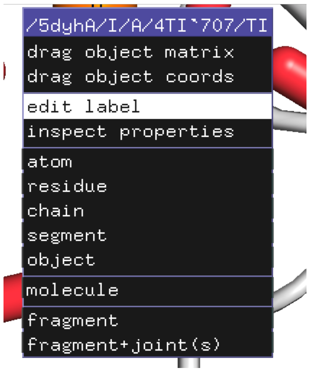

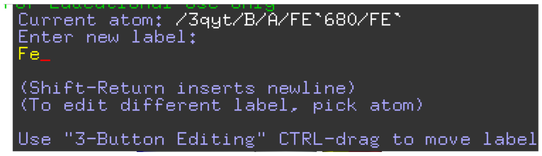

E. Labeling the Bonds to Fe:

1. Click on Wizard and then select Measurement.
2. Select the two atoms that you want to measure the bond length. For instance, click on Fe and an O to obtain a bond length between them.
3. Delete the label of the bond length by clicking on Measurement and then Labels and hit the delete button.
4. You can color code the different bond lengths as shown in image 6 below.

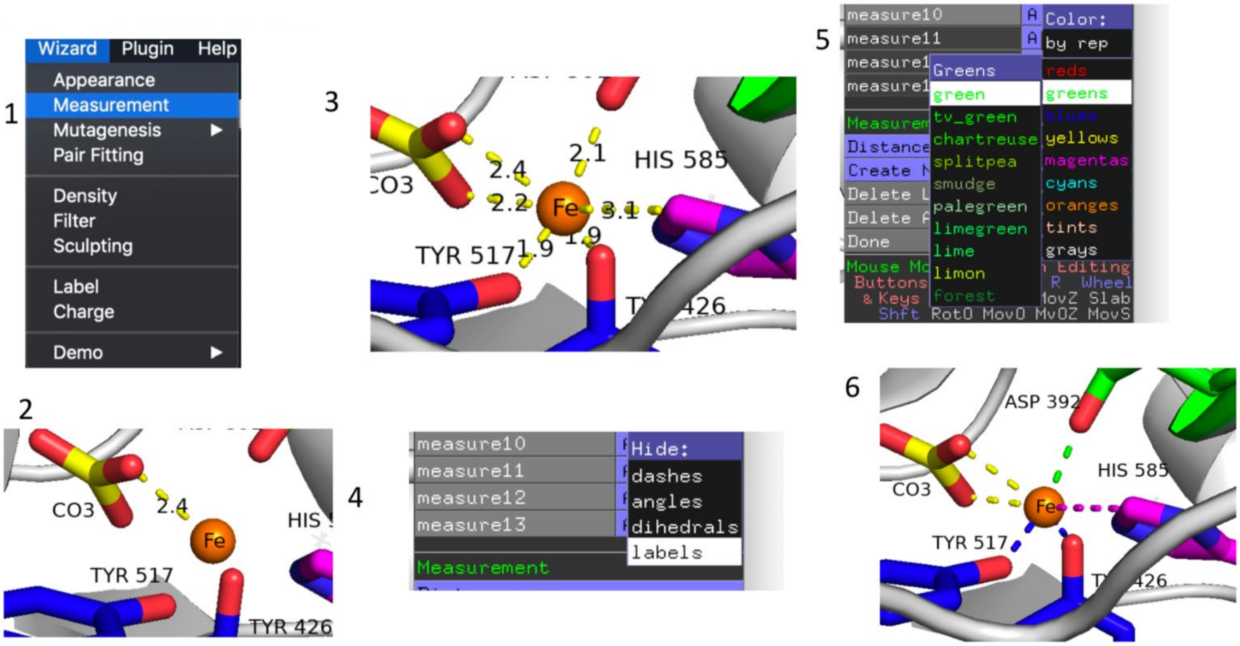

F. Figure Optimization:

1. Click on Setting and then Edit All.
2. Find Dash_radius and set it to 0.08.
3. Find Stick_radius and set it to 0.2
4. Click on Setting →Label→ Size and then set to 18 for better readability.

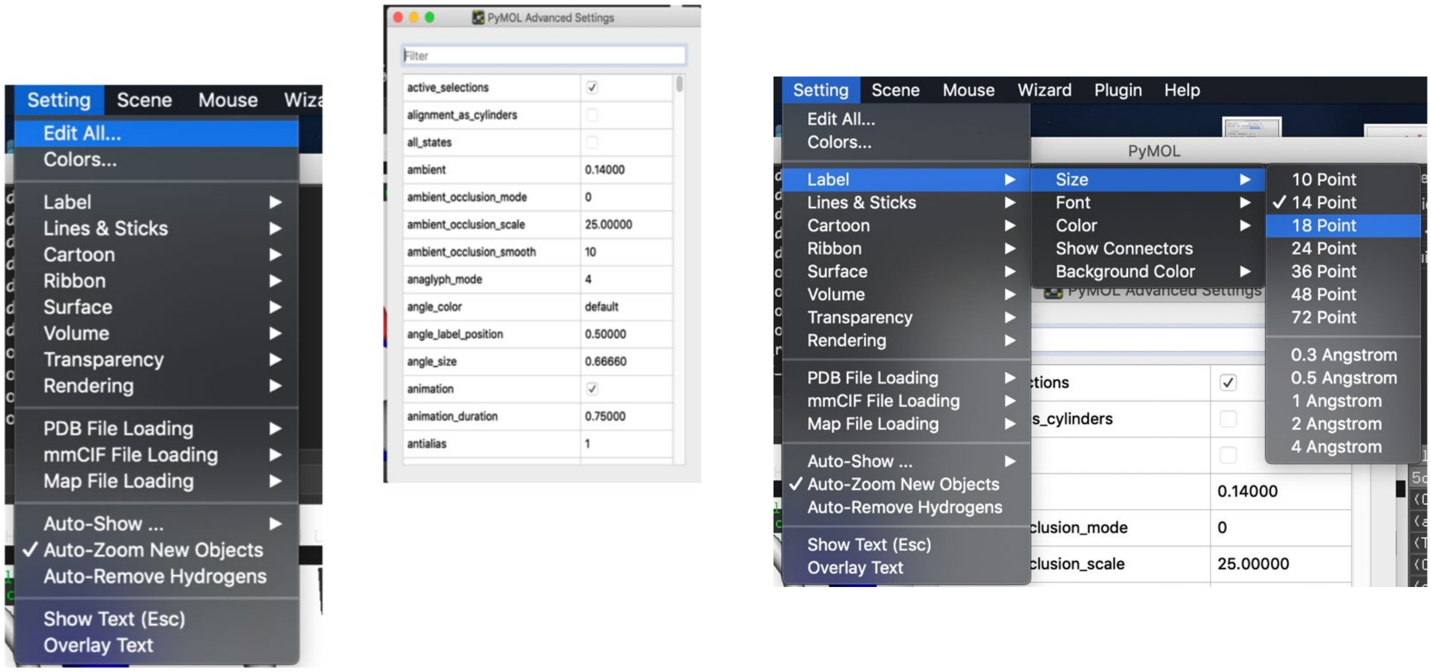

5.

G. Exporting the Image:

1. Go to the right hand side of the Upper control panel.

2. Click on Draw/Ray and then enter the parameters in the figure below.

3. Select Draw (fast). Then Save Image to File.

4. You can export the final optimized image for further analysis or to include in your report.

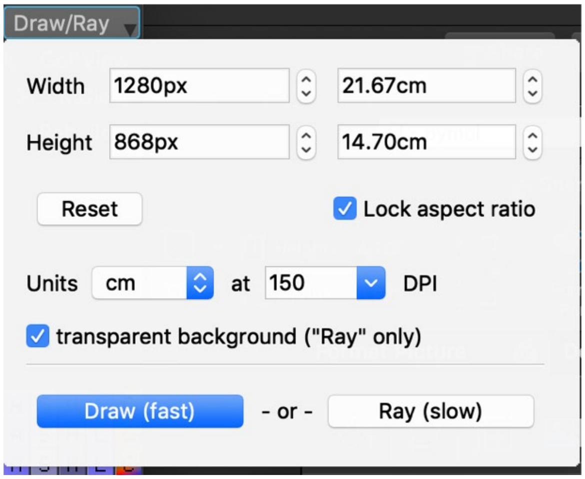

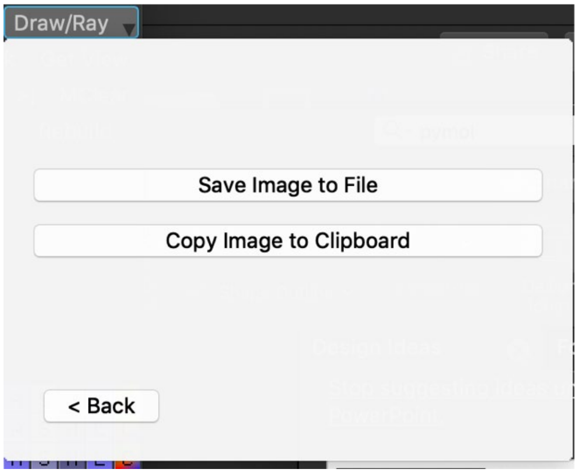

H. Additional Modifications:

1. To make additional modifications to the visualization of the protein you can refer to this video: **https://www.youtube.com/watch?v=wiKyOF-pGw4**.
