# Supplementary material for: Reinforcing Protein Biochemistry: A Two-Week Experiment Studying Iron(III) Binding by the Transferrin Protein through Stoichiometric Determination, Stability Analysis, and Visualization of the Binding Site
Source: J Chem Educ. 2024 Mar 26;101(4):1656–64. doi: 10.1021/acs.jchemed.3c01016 (PMC11033862; doi:10.1021/acs.jchemed.3c01016)

Supporting Information

Reinforcing Protein Biochemistry: A Two-Week Experiment Studying Iron(III) Binding by the Transferrin Protein through Stoichiometric Determination, Stability Analysis, and Visualization of the Binding Site

Josué A. Benjamín-Rivera[1,ǂ] Mariela Pérez Otero[2,ǂ] Arthur D. Tinoco[1*]

[1]Department of Chemistry, University of Puerto Rico, Río Piedras Campus, Río Piedras, Puerto Rico 00931, United States.

[2]Department of Biology, University of Puerto Rico, Río Piedras Campus, Río Piedras, Puerto Rico 00931, United States.

[ǂ]Equal contribution

* Email: atinoco9278@gmail.com

Supporting Information E

Lecture Slides

Table of Content

	Page
Lecture	S3-S28
Laboratory Experience Overview	S29-S34

Exploring key features of Fe(III) binding by the blood transporter transferrin

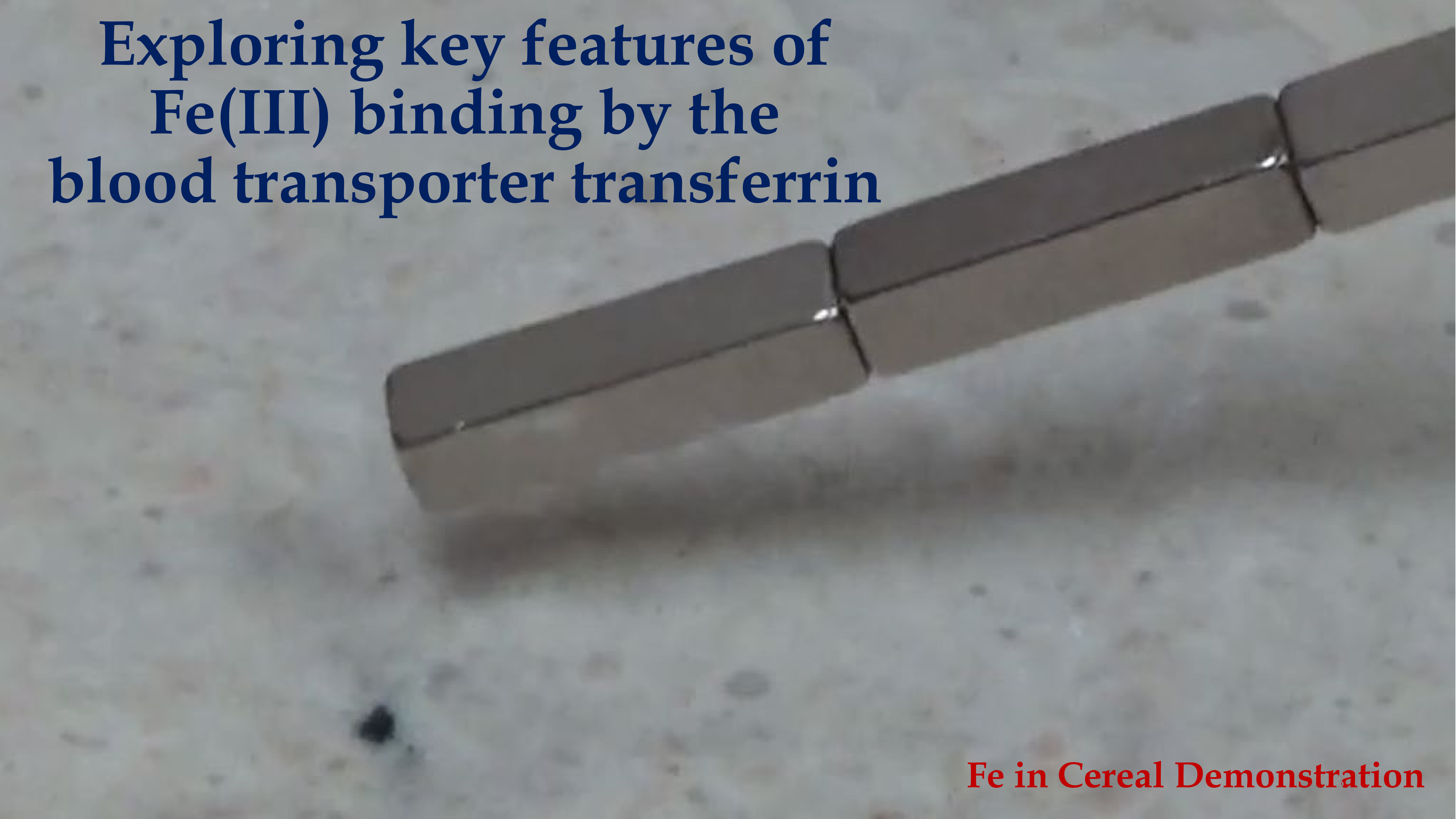

Fe in Cereal Demonstration

1. Multiple iron (Fe) functions in living organisms

Fe is an essential metal, which is largely found associated with proteins. Through its interaction with proteins, Fe displays many functions important for human survival. Below is a selection of important Fe-proteins.

Protein	Function
**Hemoglobin**	Oxygen transport
**Myoglobin**	Oxygen storage
**Cytochromes**	Electron transport/Respiration/ATP synthesis/Apoptosis
**Ribonucleotide reductase**	Deoxyribonucleotide/DNA synthesis
**Aconitase**	Citric acid cycle
**Transferrin**	Iron transport
**Lactoferrin**	Iron binding, Antimicrobial
**Ferritin**	Iron storage
**Dehydrogenases**	Electron transfer
**Hydroxylases**	Detoxification
**Catalase**	Decomposition of hydrogen peroxide
**Hemopexin**	Heme delivery

2. Food as a source of iron

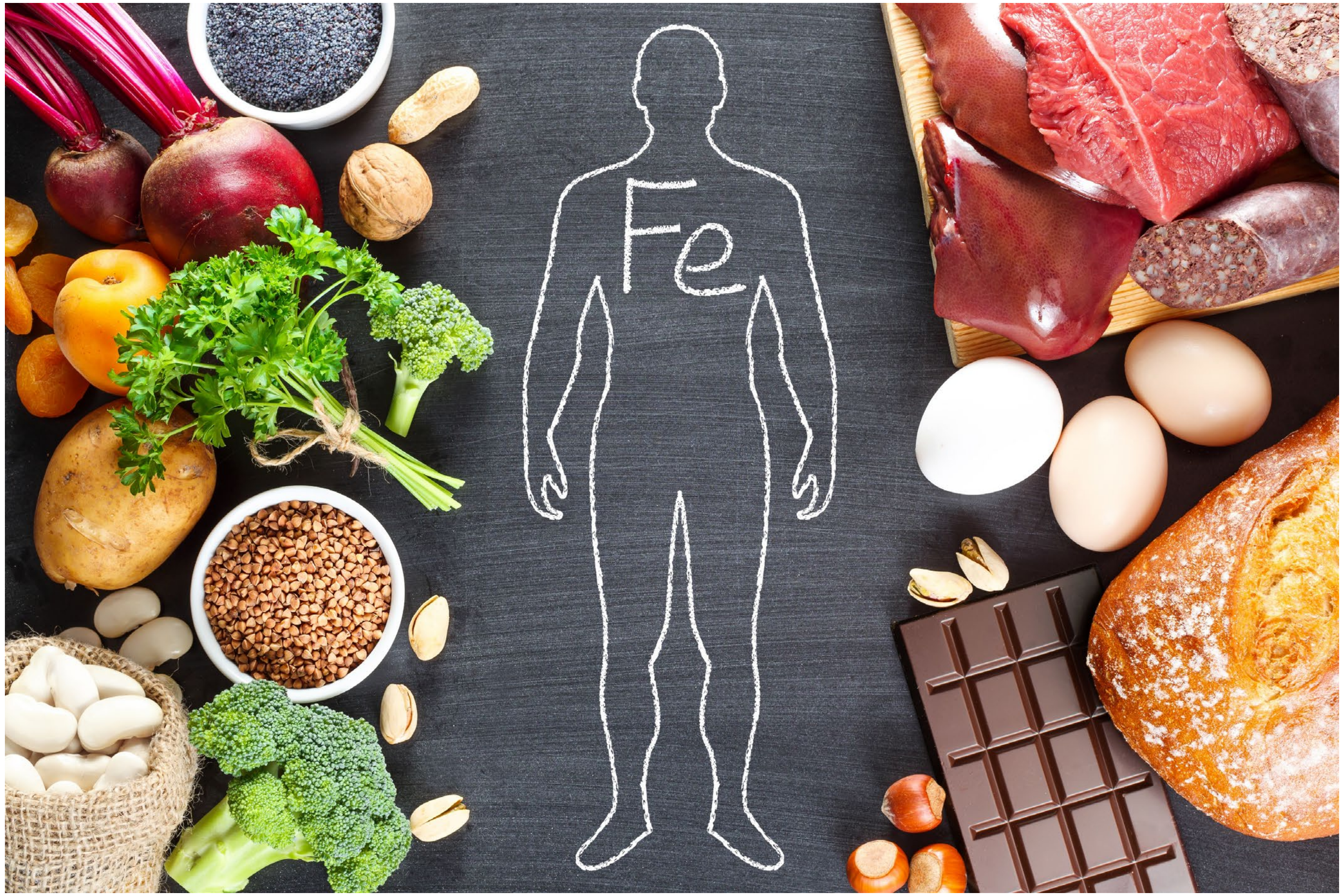

iStock.com/plotr_malczyk

- Fe can be ingested in elemental form (as in fortified cereal) or in heme (Fe^{2+}) or nonheme (Fe^{3+}) biomolecular form

- During the digestion process, the stomach pH (~3-4) becomes more acidic (~1-2)

2A. How do we obtain soluble and bioavailable iron?

Production of Fe^{2+} in the stomach:

I. Elemental iron is dissolved via acidification coupled with oxidation to Fe^{2+}

$$2\,H^+(aq) + Fe(s) \longrightarrow H_2(g) + Fe^{2+}(aq)$$

II. Dietary Fe^{3+} is reduced to Fe^{2+}

Ferrireductase (cell surface)
Or reductants in the diet (ascorbic acid)

$$Fe^{3+}(aq) \longrightarrow Fe^{2+}(aq)$$

III. Fe^{2+} release from protein degradation

2A. How do we obtain soluble and bioavailable iron?

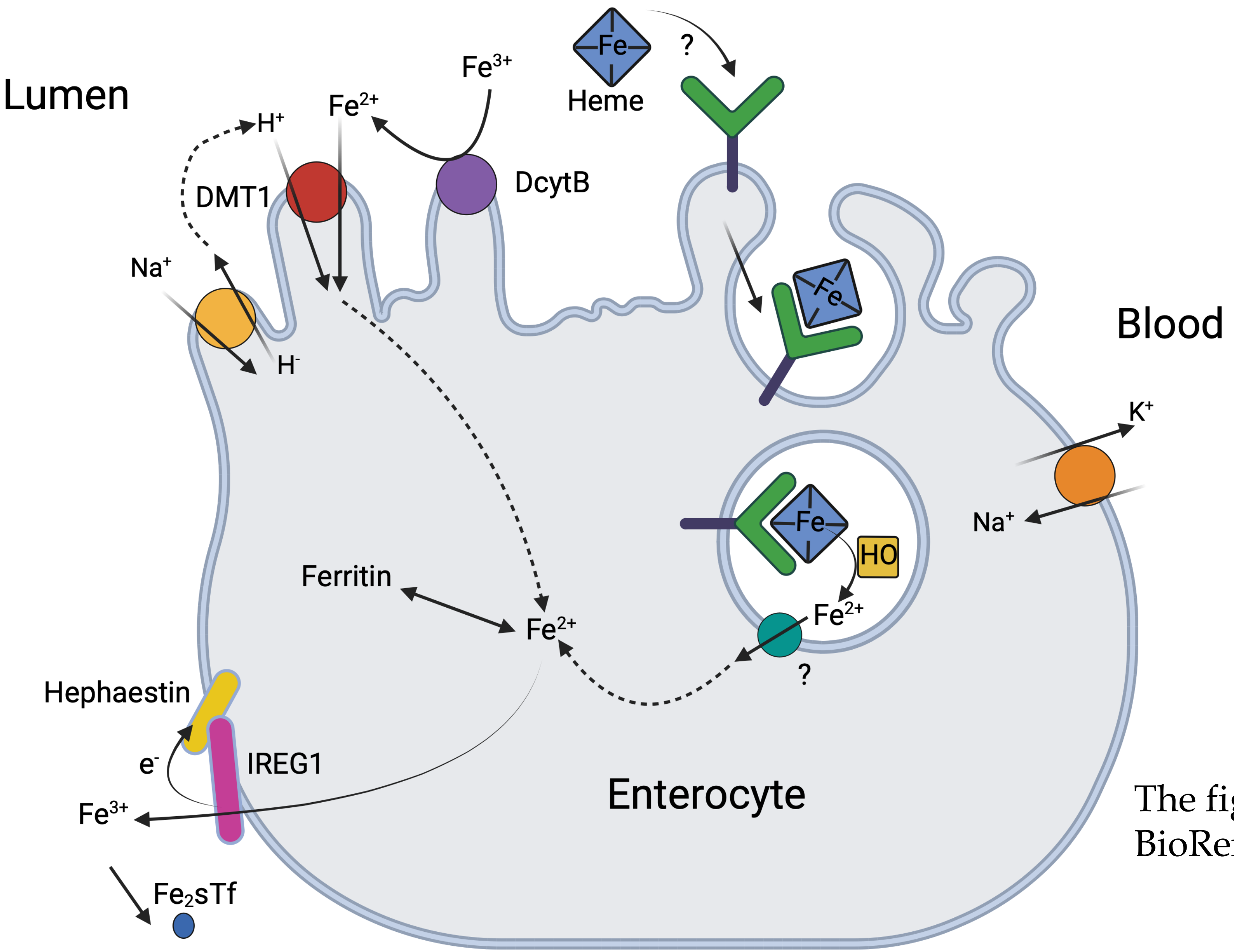

The figure was created with BioRender.com.

2B. Low Fe(III) aqueous solubility

Absorption from the intestine and into the blood (pH 7.4) results in oxidation:

$Fe^{2+}(aq) \rightleftarrows Fe^{3+}(aq) + e^-$

At this pH, precipitation would occur:

$Fe^{3+}(aq) + 3\ OH^-(aq) \rightleftarrows Fe(OH)_3\ (s)$

How much soluble Fe(III) would there be?

$Fe(OH)_3\ (s) \rightleftarrows Fe^{3+}(aq) + 3\ OH^-(aq)$

K_{sp} (Solubility product) = $[Fe^{3+}][OH^-]^3 \sim 10^{-38}$ M

At pH 7.0, $[Fe^{3+}] = 10^{-38} / (10^{-7})^3 = 10^{-17}$ M **Extremely low solubility**

2C. Serum transferrin maintains Fe(III) solubility

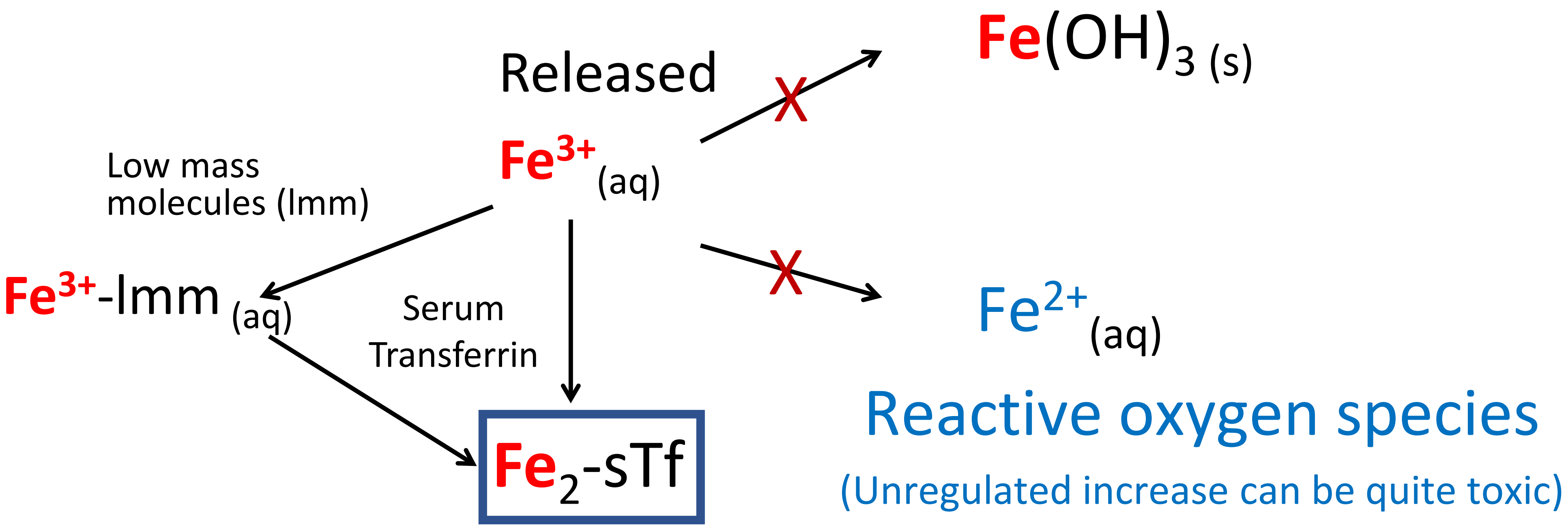

Under normal conditions, Fe(III) is virtually 100% serum transferrin bound (sTf)

3. Human sTf

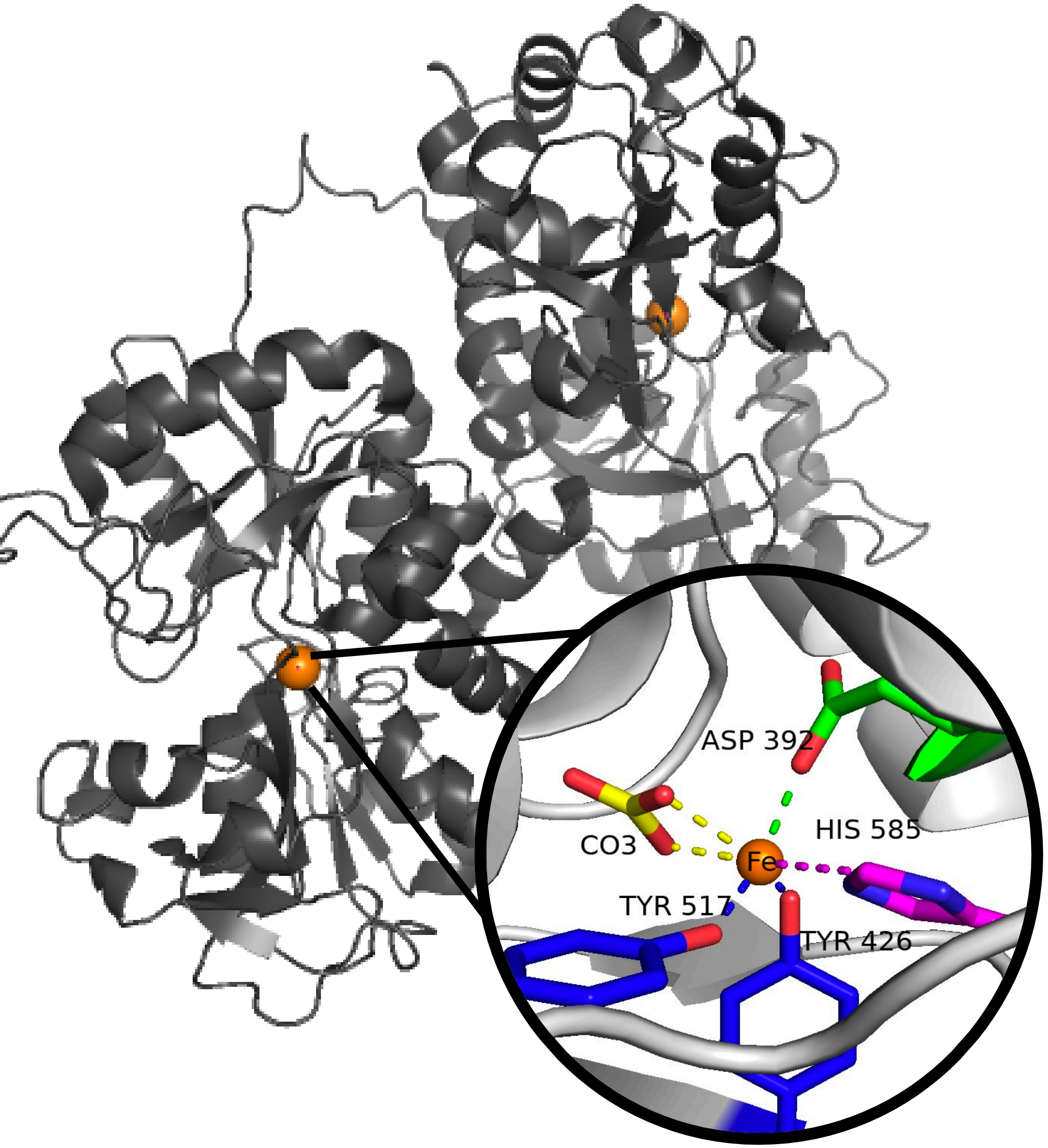

I. A member of the transferrin family of proteins

- Known to be either monolobal (~ 40 kD) or bilobal (~ 80 kD)

II. Multiple Functions

- Iron transport/homeostasis
- Bacteriostasis
  - Pathogenic bacteria thrive on iron and HsTf (and lactoferrin) serves as a strong chelator that prevents them from having access to it.

II. Binds Fe(III) with high affinity

- Fe-Tf C lobe: log K = 22.2
- Fe-Tf N lobe: log K = 21.3

3A. Distinctive spectroscopic signal for Fe(III) binding

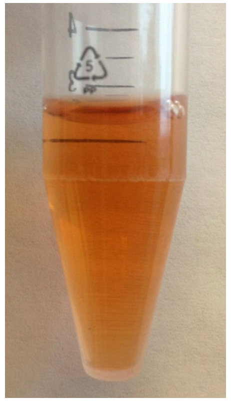

Ligand to metal charge transfer (LMCT) absorbance band (470 nm) due to tyrosine binding of Fe(III); produces characteristic pink color.

- ➢ The increase in absorbance due to Fe(III) binding to the two sites is comparable.

ε = 5,000 $M^{-1}cm^{-1}$ based on [protein]

ε = 2,500 $M^{-1}cm^{-1}$ based on [Fe(III)]

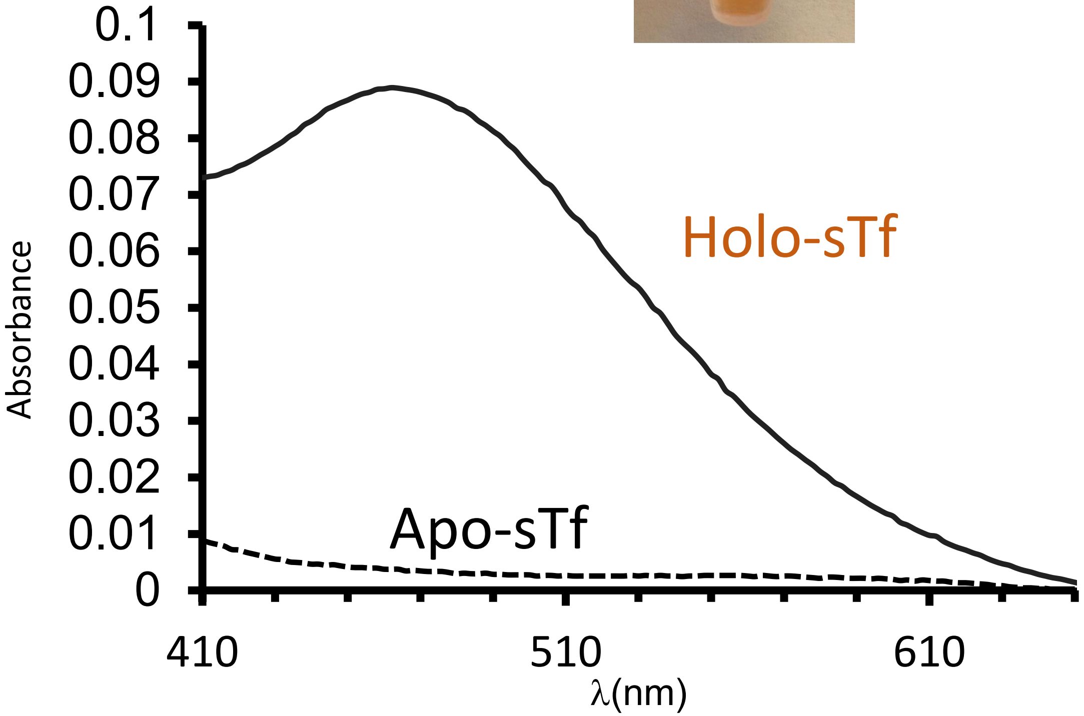

3B. sTf has a high affinity for Hard metals

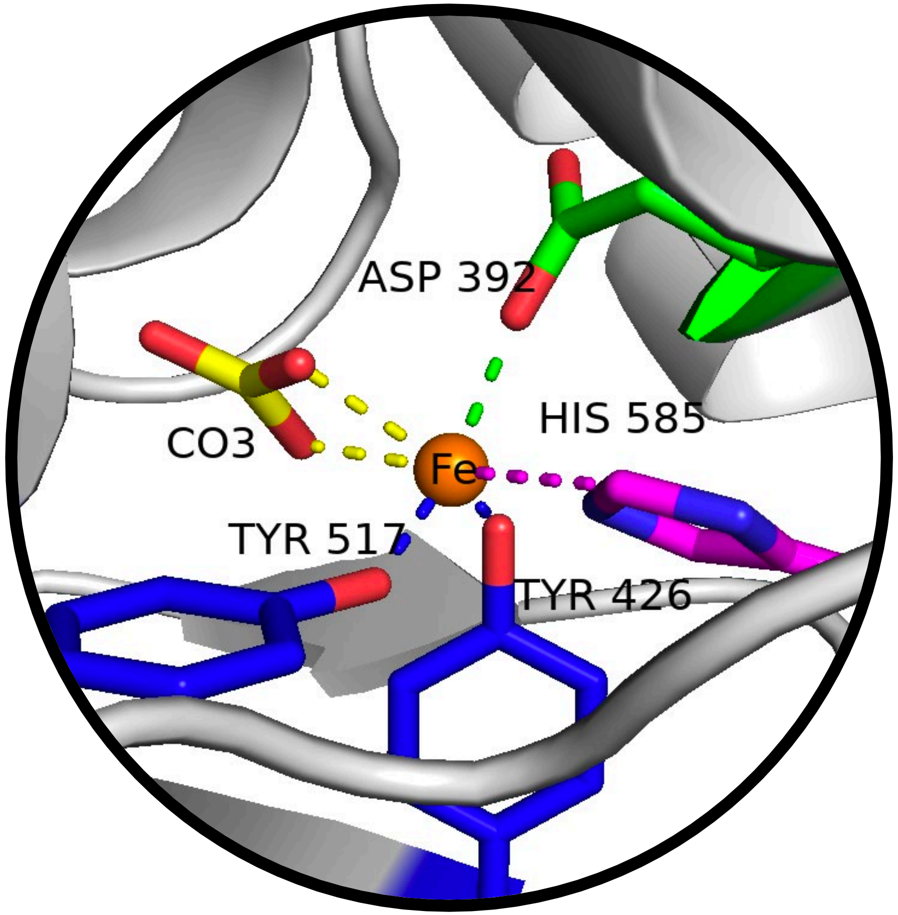

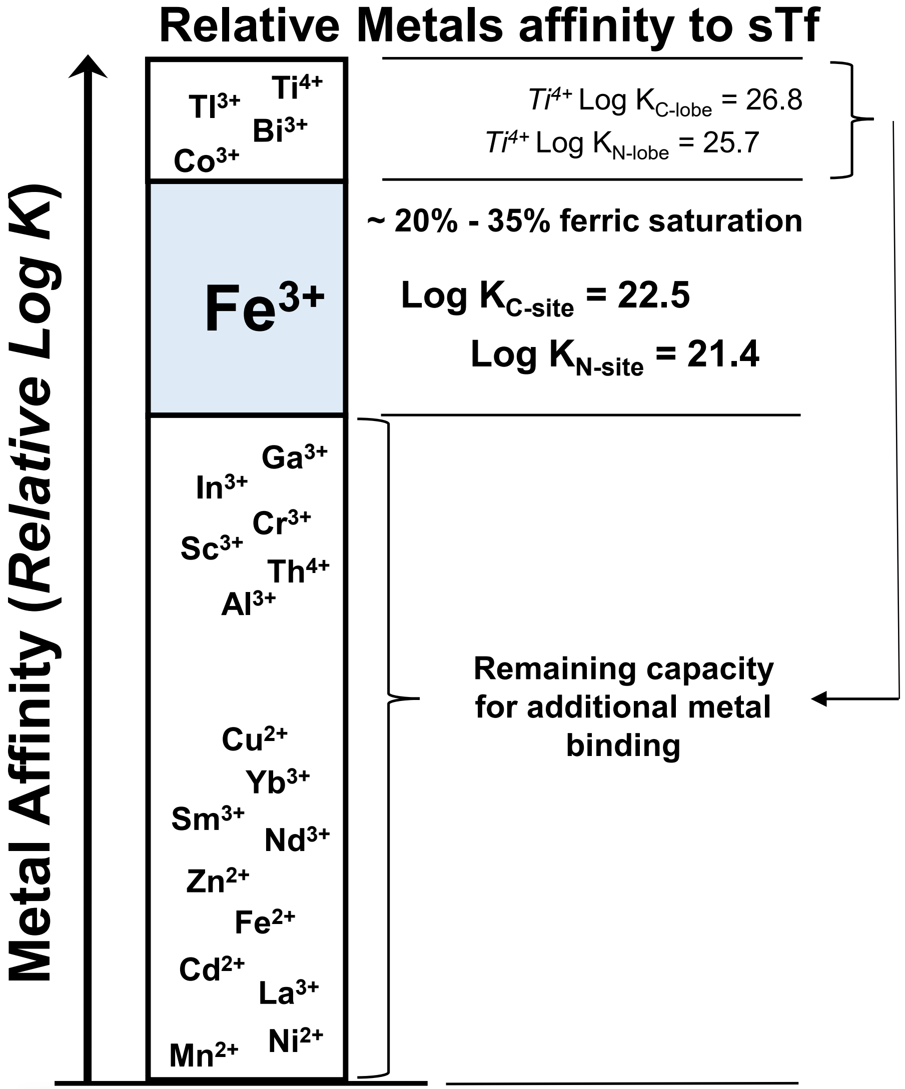

Metal affinity (log K_1) for OH^- is correlated with affinity for HsTf (C-lobe).

Image reprinted with modifications from Inorganics, 2020, 8(9), J.A. Benjamin-Rivera; A.E. Cardona-Rivera; A.L. Vazquez-Maldonado; C.Y. Dones-Lassalle; H.L. Pabon-Colon; H.M. Rodriguez-Rivera; I. Rodriguez; J.C. Gonzalez-Espiet; J.F. Catala-Torres; M. Carrasquillo Rivera; M.G. De Jesus-Soto; N.C. Cordero-Virella,; P.M. Cruz-Maldonado; P. Gonzalez-Pagan; R. Hernandez-Rios; K. Gaur; S.A. Loza-Rosas; A.D. Tinoco Exploring serum transferrin regulation of nonferric metal therapeutic function and toxicity. doi: 10.3390/inorganics8090048, No special permission is required by MDPI.

3C. Fe(III) binding alters sTf conformation

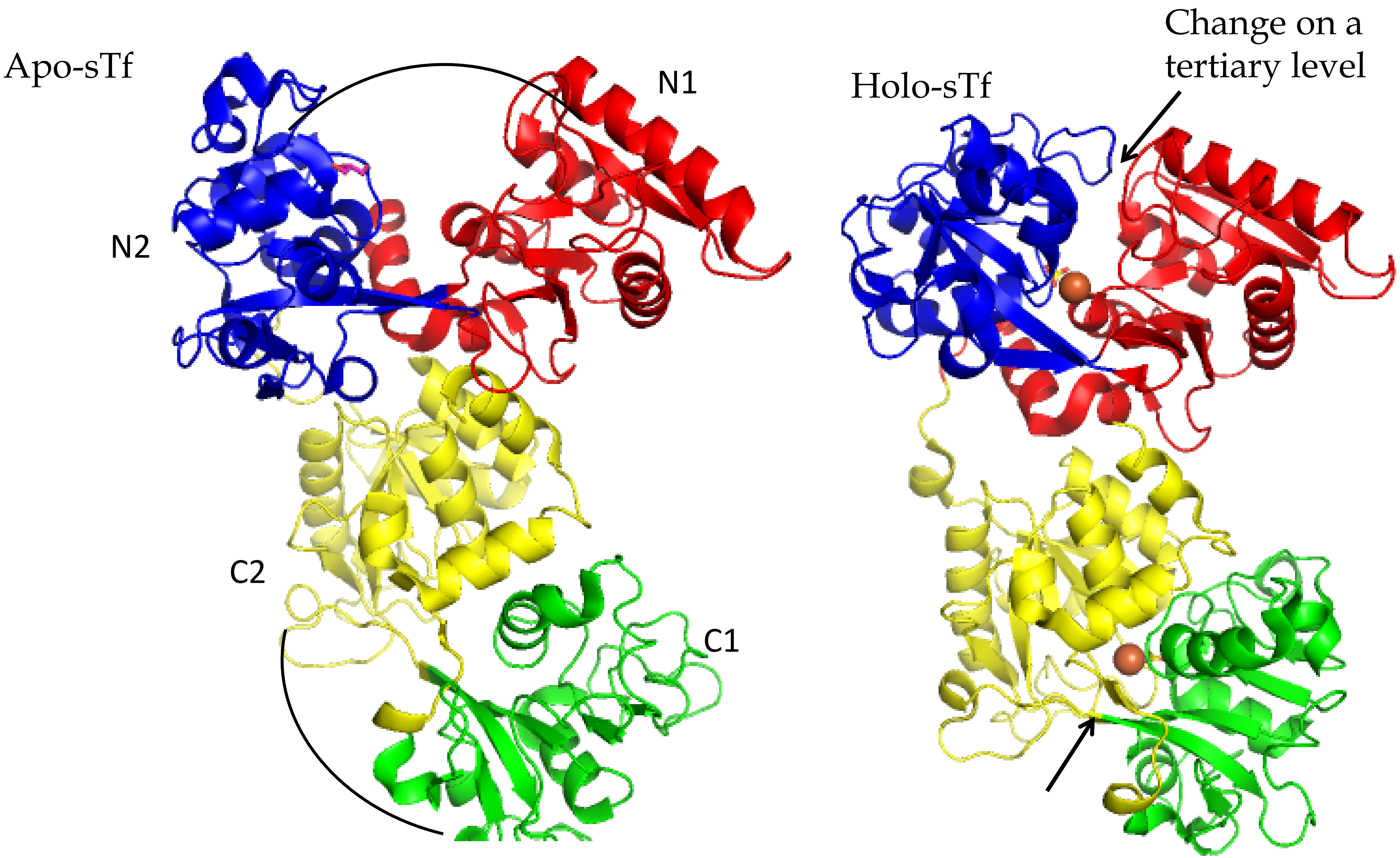

Apo PDB 2HAU , Holo PDB 3V83 **Fe(IIII) binding stabilizes both sTf lobes.**

3C I. N-site Fe(III) binding site

Metal binding residues:

N1: 1-92 and 247-330

N2: 93-246

- Asp63 (N1)
- His249 (N1)
- Tyr95 (N2)
- Tyr188 (N2)

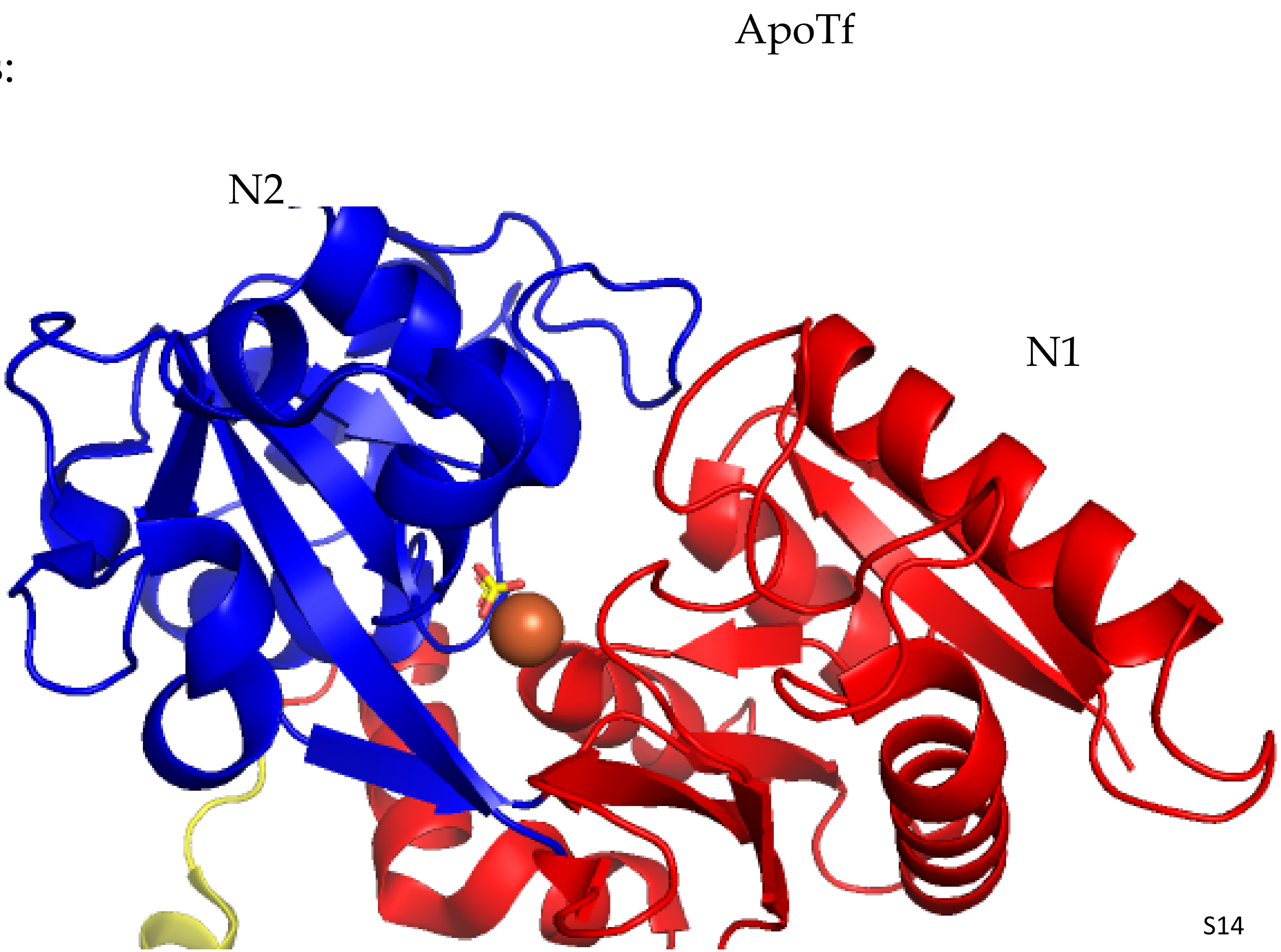

3C II. C-site Fe(III) binding site

Metal binding residues:

C1: 340-425 and 573-679

C2: 426-572

- Asp392 (C1)
- Tyr426 (C2)
- Tyr517(C2)
- His585 (C1)

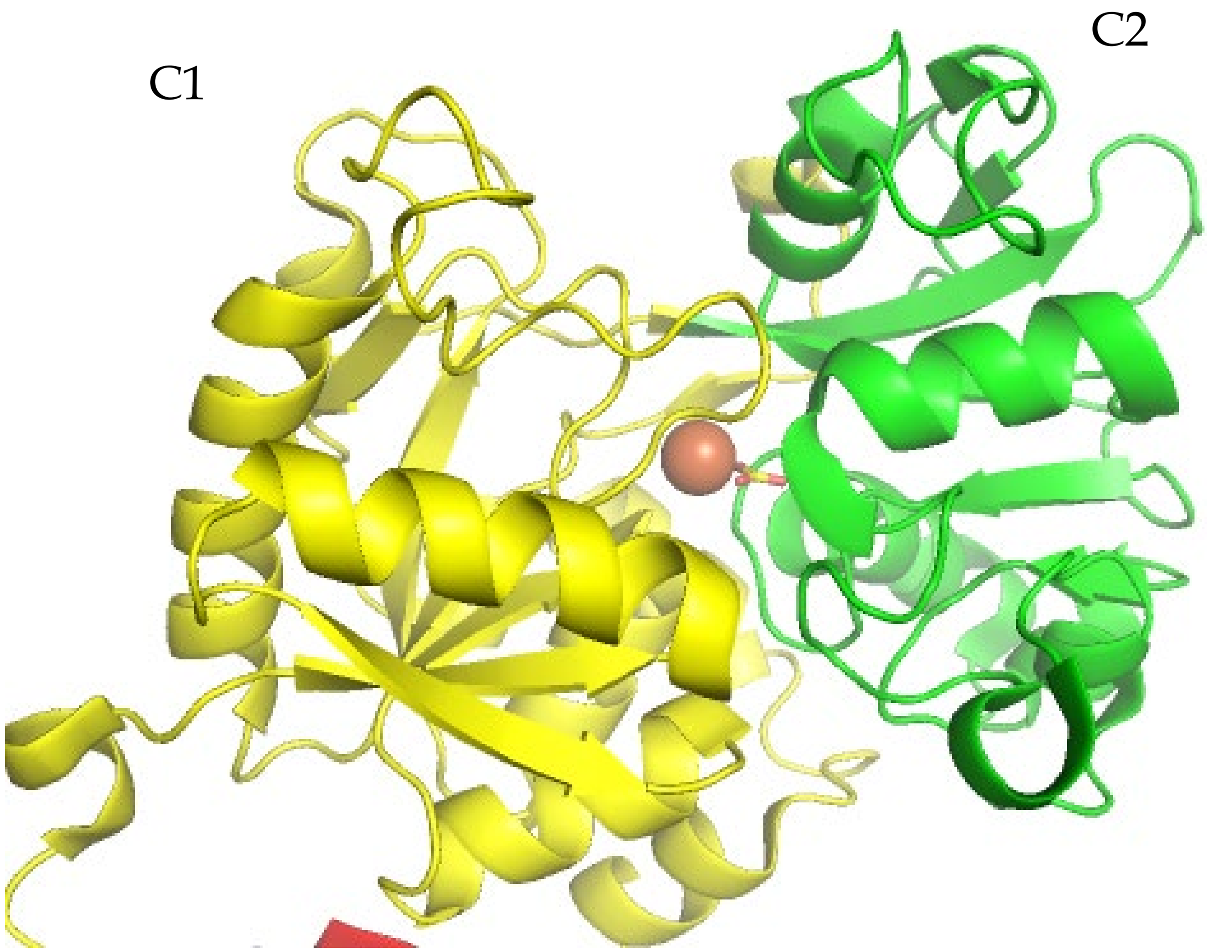

3D. Differences in Fe(III) binding to the N-lobe and C-lobe

Similarities and Differences in secondary sphere of coordination

Carbonate binding sites:

Noncovalent interactions:

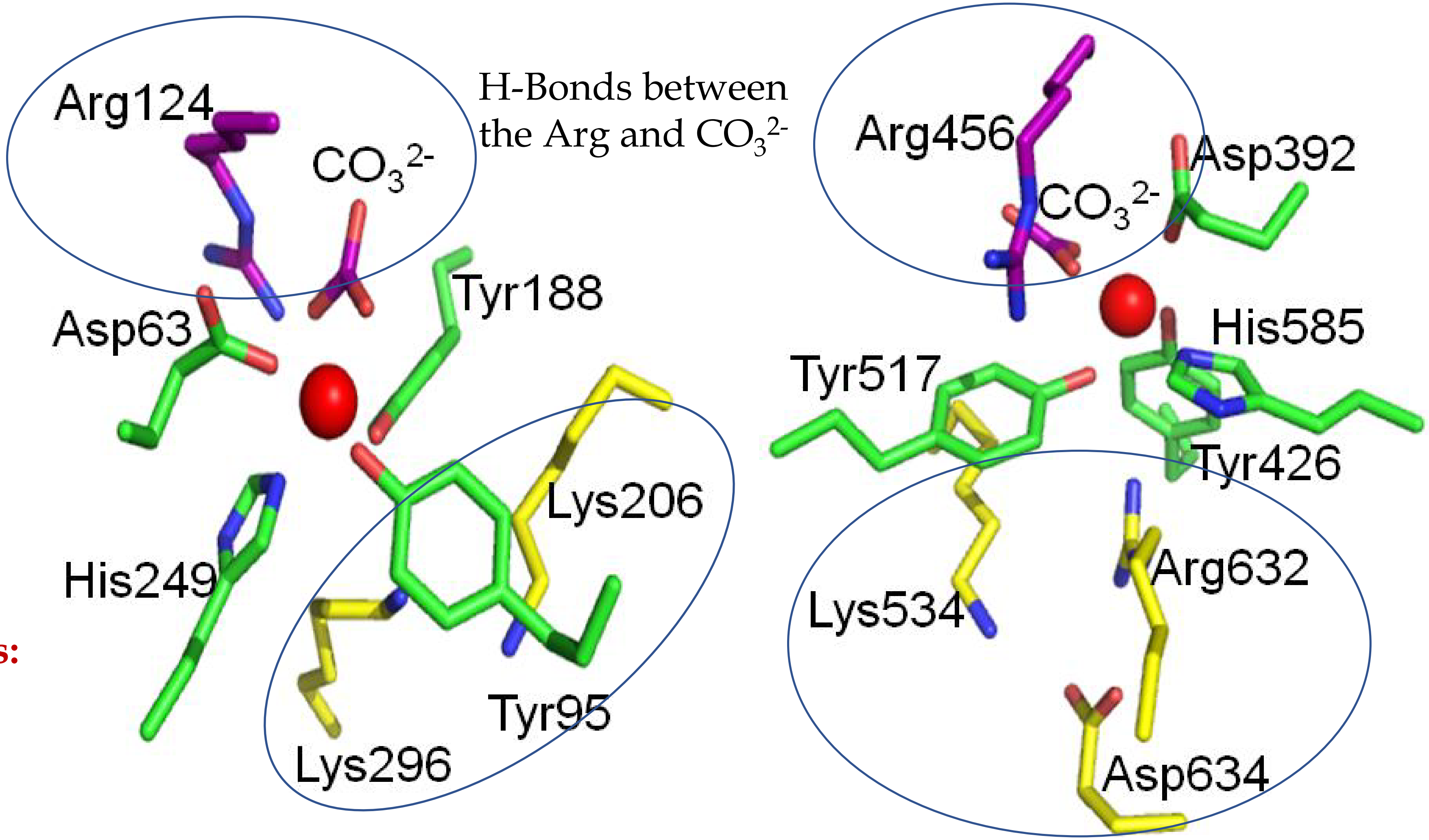

N-lobe **C-lobe**

He, Q. Y.; Mason, A. B.; Tam, B. M.; MacGillivray, R. T. A.; Woodworth, R. C. *Biochemistry* **1999**, *38* (30), 9704-9711.

3D I. The pH sensitive dilysine interaction in the N-lobe

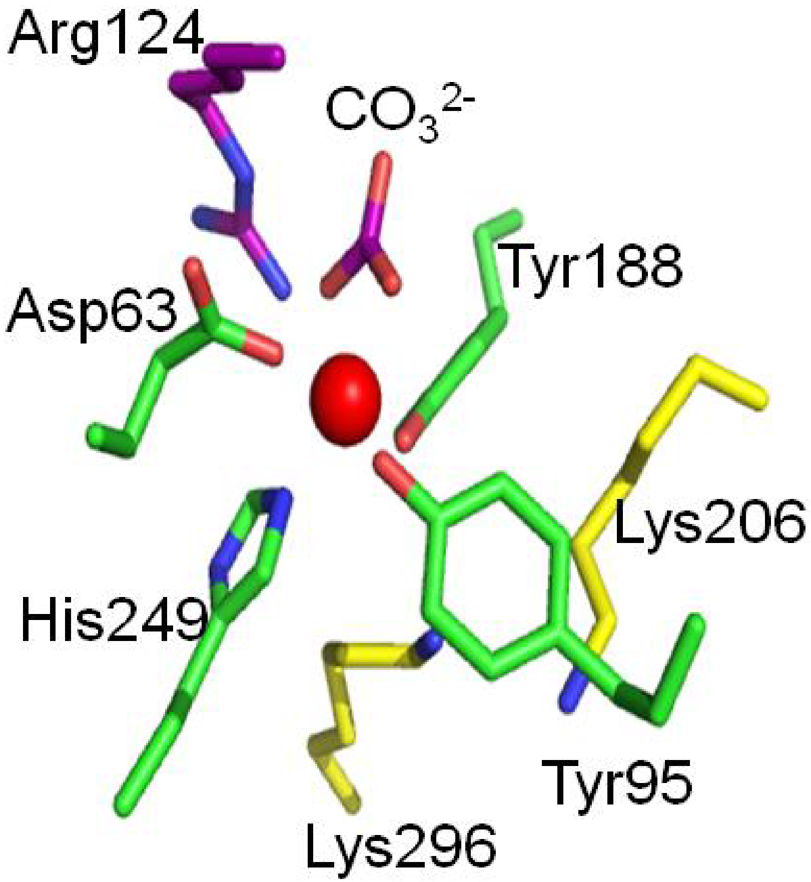

Fe(III) Coordination lowers the pKa of K206 and K296

- Normal pKa is 10.53, positively charged at pH 7.4
  Recall, if pH < pKa, then will be protonated, especially if more than 1 pH unit lower.
  pH > pKa, then deprotonated

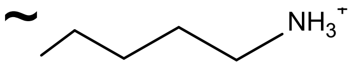

- In the Fe(III) bound closed conformation structure, one of the Lys is deprotonated and the two Lys residues engage in H-Bond via a single H+

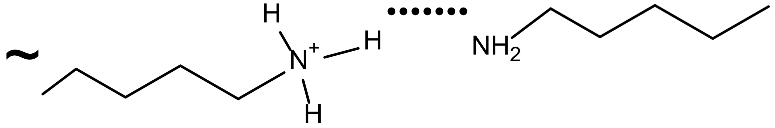

- This hydrogen bond interaction is stable even at pH 5.5.

Gumerov, D.R. and Kaltashov, I.A. *Anal Chem*. **2001**, *73*, 2565-2570.

3D I. The pH sensitive dilysine interaction in the N-lobe

- The H-Bond is stabilized by decreased exposure to solvent and the hydrophobic box created by Y188, Y95, and H249, which favors lower charge.

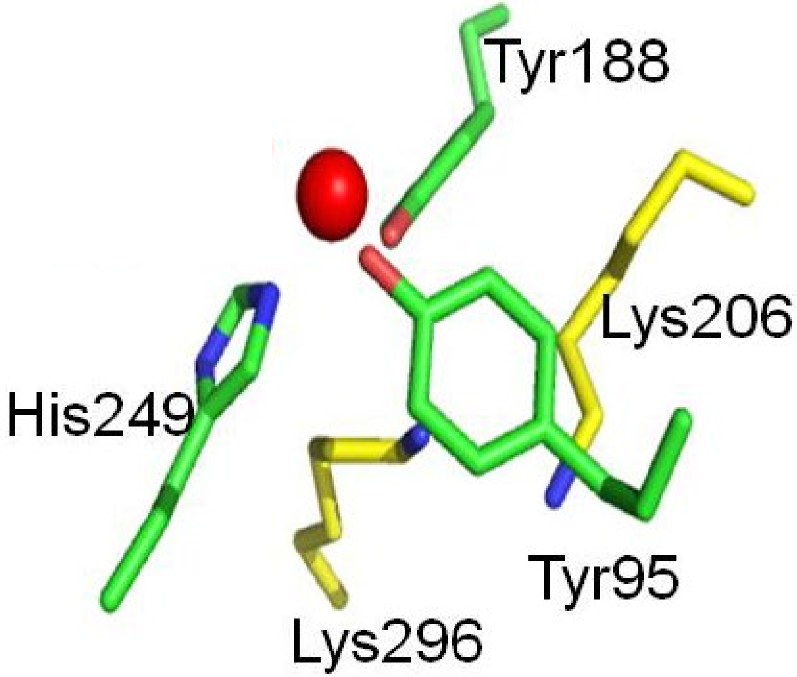

- A sudden influx in protons would disrupt this interaction and lead to the “dilysine trigger” of a conformational change that opens up the protein.

Closed ⟶ **Open**

3D II. The pH sensitive triad in the C-lobe

Fe(III) coordination lowers the pKa of K534 and R632 and results in a H-Bond network with each other and D634. There is also an electrostatic attraction between the positively charged amino acids with the negatively charged amino acid.

- This H-Bond network and electrostatic interactions are stable even at pH 5.5 due to YYH hydrophobic box.

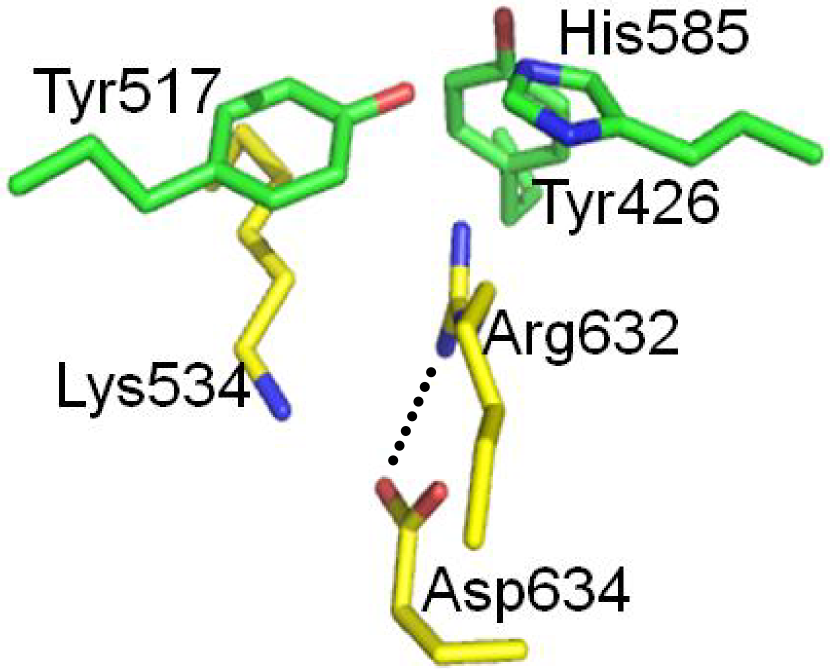

Halbrooks, P.J. *et al*. *Biochemistry*. **2005**, *44*, 15451-15460.

3E. Fe(III) binding stabilizes sTf

There are numerous ways to measure the stability of a protein and it is often done by using either a chemical or thermal method to examine the transition from a folded to unfolded (denatured) state.

- These methods can be used to examine stability differences between different protein conformations .

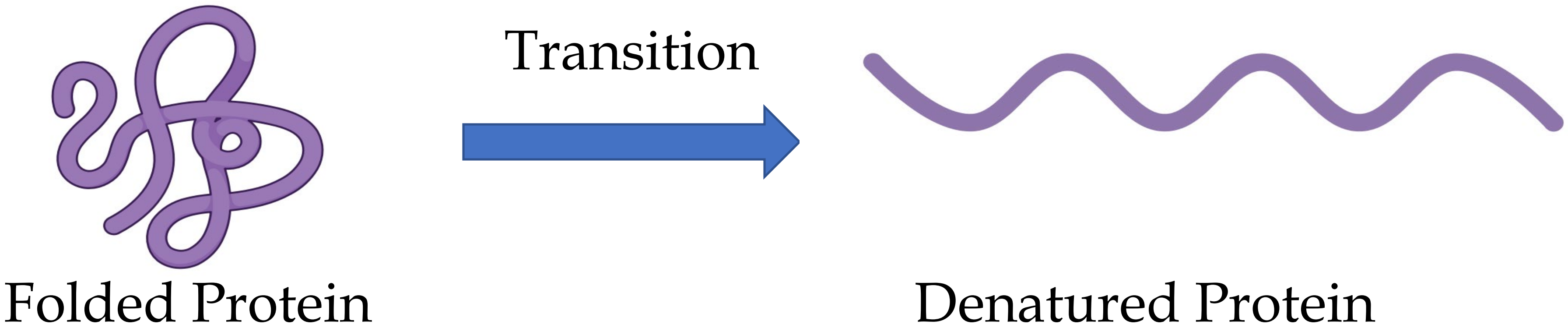

3E. Fe(III) binding stabilizes sTf

Urea is a chemical denaturant that at high concentrations can interact with proteins noncovalently to trigger their unfolding.

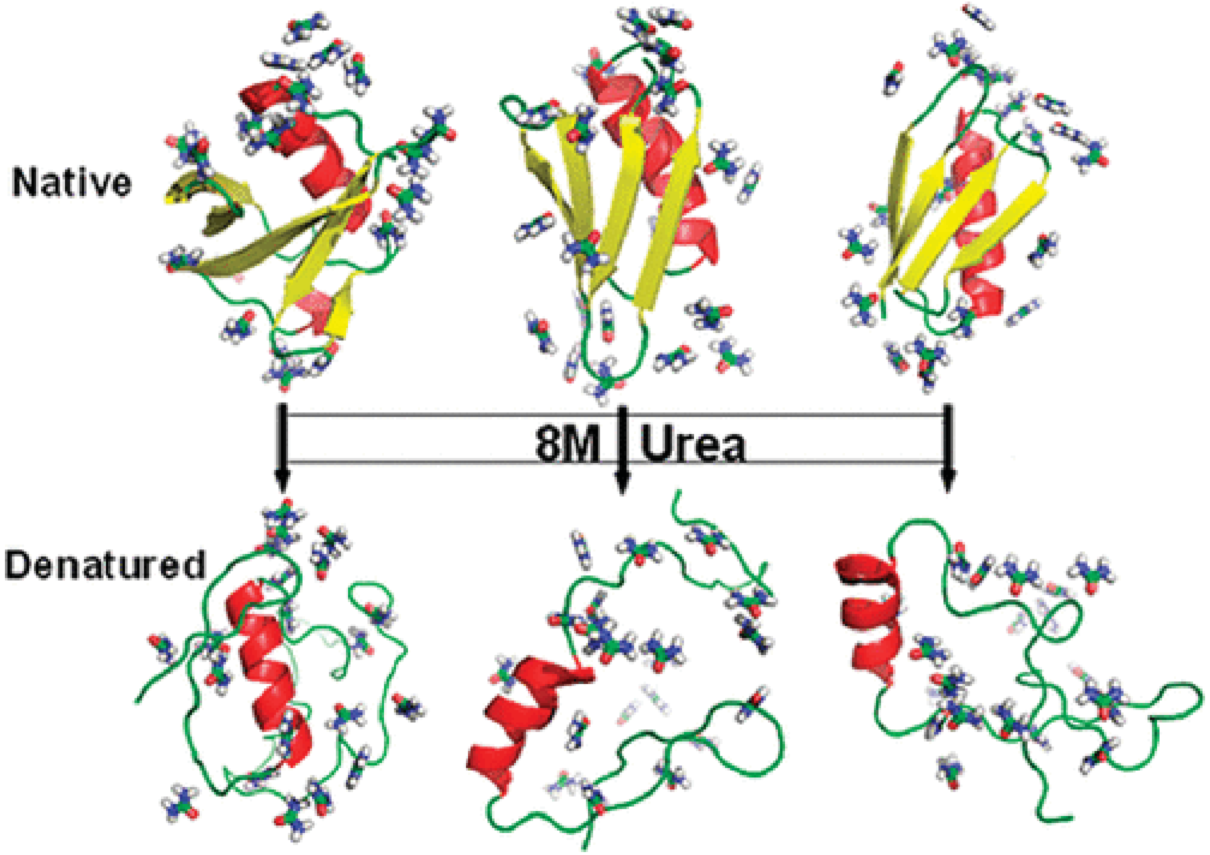

Reprinted from Journal of Physical Chemistry B, 113, A. Das, C. Mukhopadhyay, Urea-mediated protein denaturation: A consensus view, 12816-12824, Copyright (2009), with permission from ACS.

3E. Fe(III) binding stabilizes sTf

Urea gel electrophoresis is a simple method for monitoring urea-induced unfolding of proteins. Proteins become unstable as they move through the gel. The most stable proteins move furthest vertically down the gel.

Reprinted from Gels, 2022, 8(1), A. Levina; B. Wang; P.A. Lay, Urea gel electrophoresis in studies of conformational changes of transferrin on binding and transport of non-ferric metal ions; https://doi.org/10.3390/gels8010019, No special permission is required by MDPI.

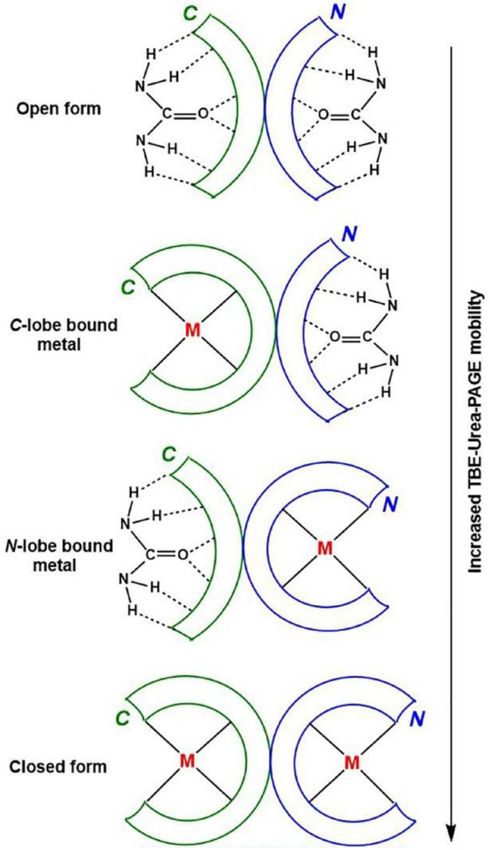

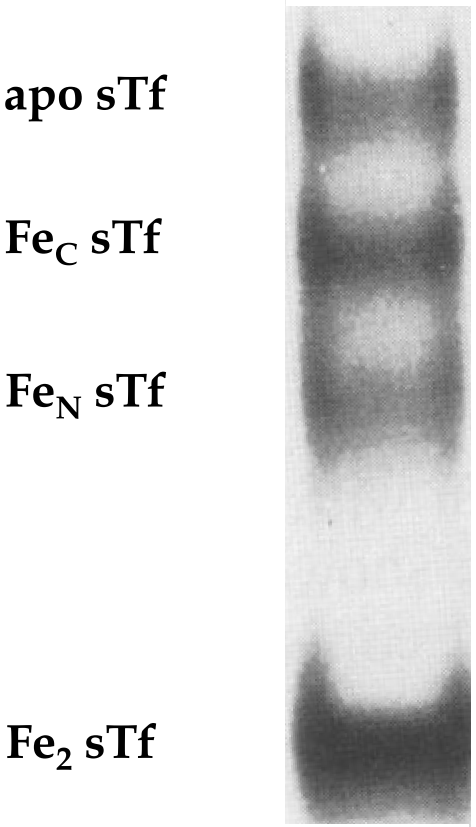

Representative Urea gel to monitor aposTf and different Fe(III)-bound sTf.

3F. Canonical versus noncanonical metal sTf binding

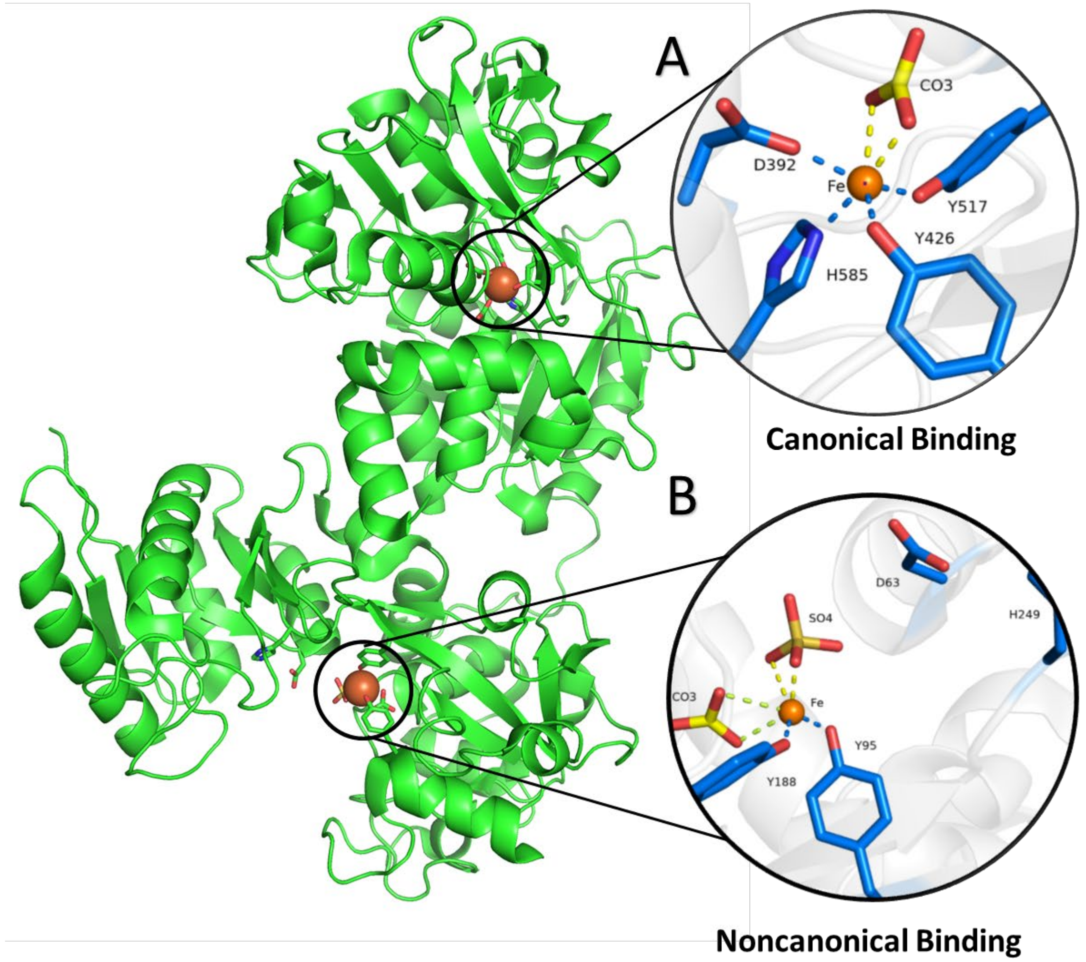

PDB 3QYT

Image reprinted from Inorganics, 2020, 8(9), J.A. Benjamin-Rivera; A.E. Cardona-Rivera; A.L. Vazquez-Maldonado; C.Y. Dones-Lassalle; H.L. Pabon-Colon; H.M. Rodriguez-Rivera; I. Rodriguez; J.C. Gonzalez-Espiet; J.F. Catala-Torres; M. Carrasquillo Rivera; M.G. De Jesus-Soto; N.C. Cordero-Virella,; P.M. Cruz-Maldonado; P. Gonzalez-Pagan; R. Hernandez-Rios; K. Gaur; S.A. Loza-Rosas; A.D. Tinoco Exploring serum transferrin regulation of nonferric metal therapeutic function and toxicity. doi: 10.3390/inorganics8090048, No special permission is required by MDPI.

4. Fe(III) transport by serum transferrin

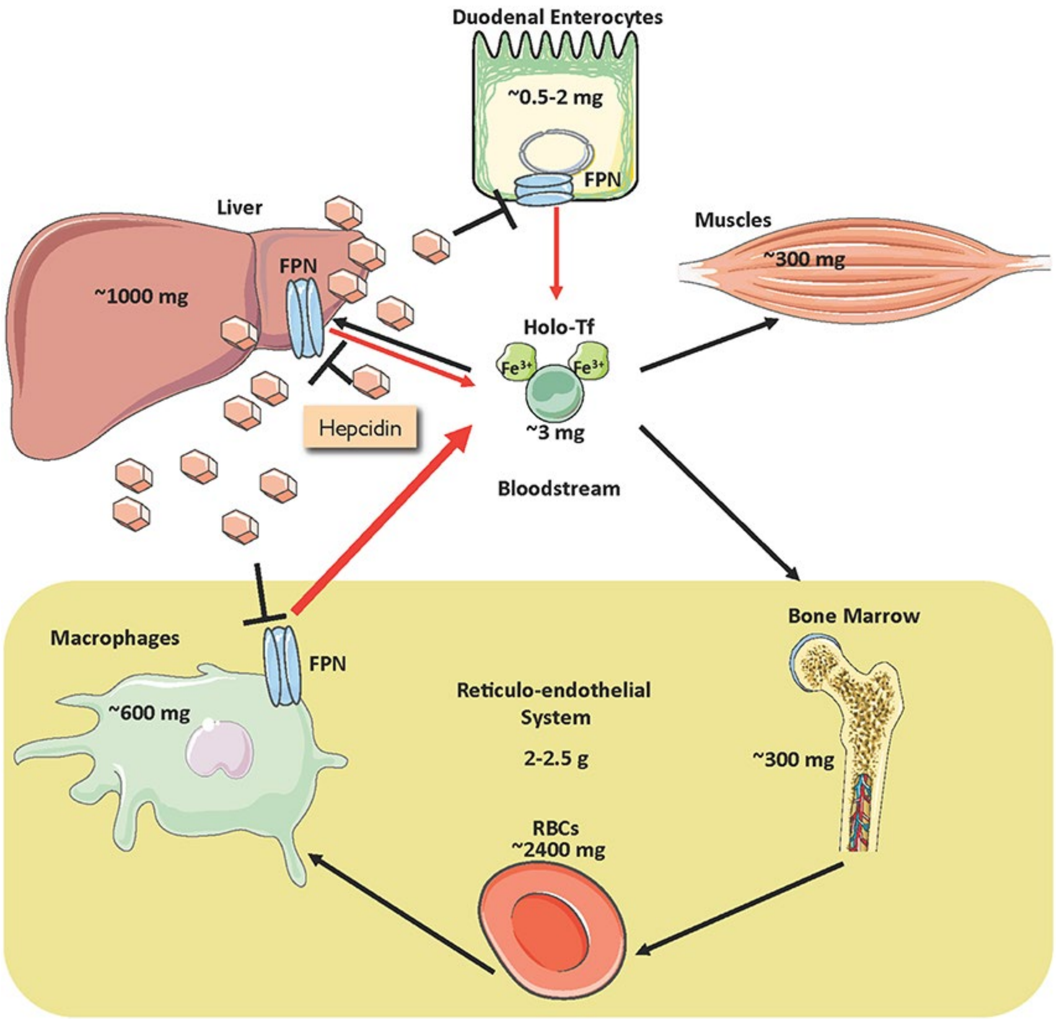

Reprinted from Frontiers in Pharmacology. G. Sebastiani; N. Wilkinson, N.; K. Pantopoulos. Pharmacological Targeting of the Hepcidin/Ferroportin Axis. *Frontiers in Pharmacology* **2016**, *7*, Review. DOI: 10.3389/fphar.2016.00160. Terms of use according to: https://creativecommons.org/licenses/by/4.0/

- Transferrin bound iron is dominant iron form in the blood.
  - Some nontransferrin bound iron (NTBI) but well-regulated
- Under normal conditions, Fe levels in blood plasma is ~20 μM and 0.001-10 μM in cell/tissue

Bertini, I.; Gray, H. B.; Stiefel, E. I.; Valentine, J. S., *Biological Inorganic Chemistry: Structure and Reactivity*. University Science Books: California, 2007.

4A. Fe(III) endocytosis by sTf

sTf uses an endocytosis process by which it internalizes Fe(III) into cells and releases it into the cytoplasm.

Key Players:

Apotransferrin (apoTf)

Holotransferrin (holoTf)

Transferrin Receptor (TfR1 and TfR2)

Clathrin

Fe^{3+} Chelator

Steap3

Divalent Metal Transporter 1 (DMT1)

Transferrin Receptor (TfR) transports Fe_2sTf into Cells

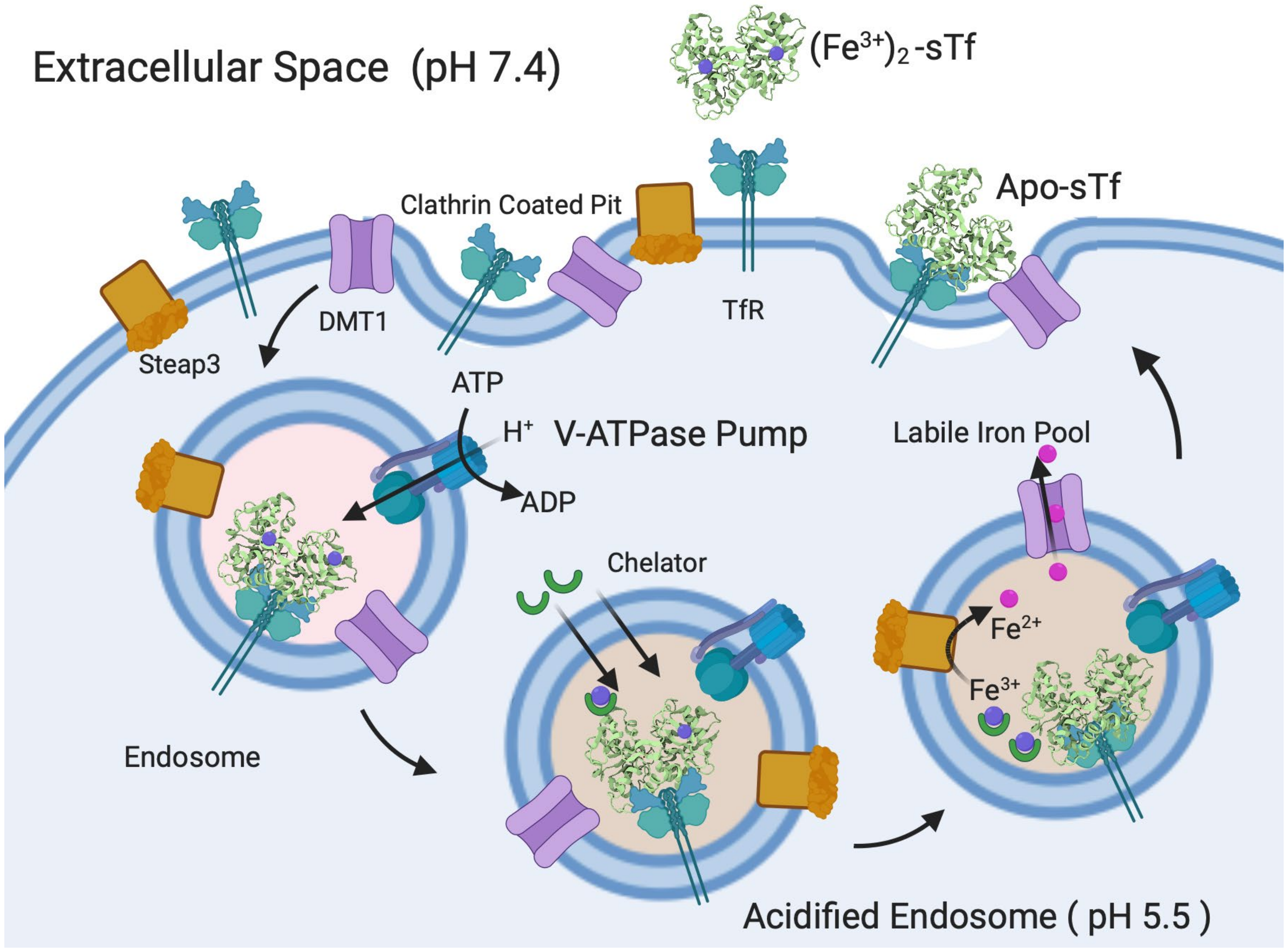

The figure was created with BioRender.com.

4B. Receptor mediated endocytosis coupled with redox and protein transporter assisted release

Steps involved:

1. pH dependent process that modulates apoTf (metal free) and holoTf (metal bound) affinity for TfR

2. Endosome formation aided by clathrin-coating to protect against lysosomal degradation and to allow recycling of Tf and TfR

3. ATP-dependent pump that lowers the endosome pH from 7.4 to 5.5

4. Iron release from sTf

5. Iron escape from the endosome as Fe^{2+}

Transferrin Receptor (TfR) transports Fe_2sTf into Cells

1. pH decrease from 7.4 to 5.5 does not trigger Fe^{3+} release from transferrin even with the higher affinity of apoTf for the TfR

2. **Fe^{3+}** chelation (possibly by ATP or citrate) coupled with metal binding site residue protonation results in **Fe^{3+}** dissociation

3. Dissociated **Fe^{3+}** is reduced to **Fe^{2+}** by Steap3

4. **Fe^{2+}** is transported to the cytosol via DMT1

Laboratory Experience Overview

Objectives

In this laboratory experiment, you will perform metal binding experiments with Fe(III) and the sTf protein, and determine how the metal binding to the protein affects the chemical properties of both species. Additionally, you will acquire skills on how to use the PyMOL application to generate a crystallized protein image.

Day 1: Prepare sTf samples containing different mole equivalents of Fe(III).

Objectives

Day 2:

A. Construct a stoichiometric curve to determine the stoichiometry of Fe(III) ions bound to sTf and the relative extinction coefficient of the metal-protein complex.

B. Run a UREA Gel to measure sTf stability after binding with the metal.

C. Familiarize yourself with the PyMOL application and learn the different methods and add-ons you can use to generate a crystallized protein image.

Construct a stoichiometric curve to determine the stoichiometry of Fe(III) ions bound to sTf and the relative extinction coefficient of the metal-protein complex.

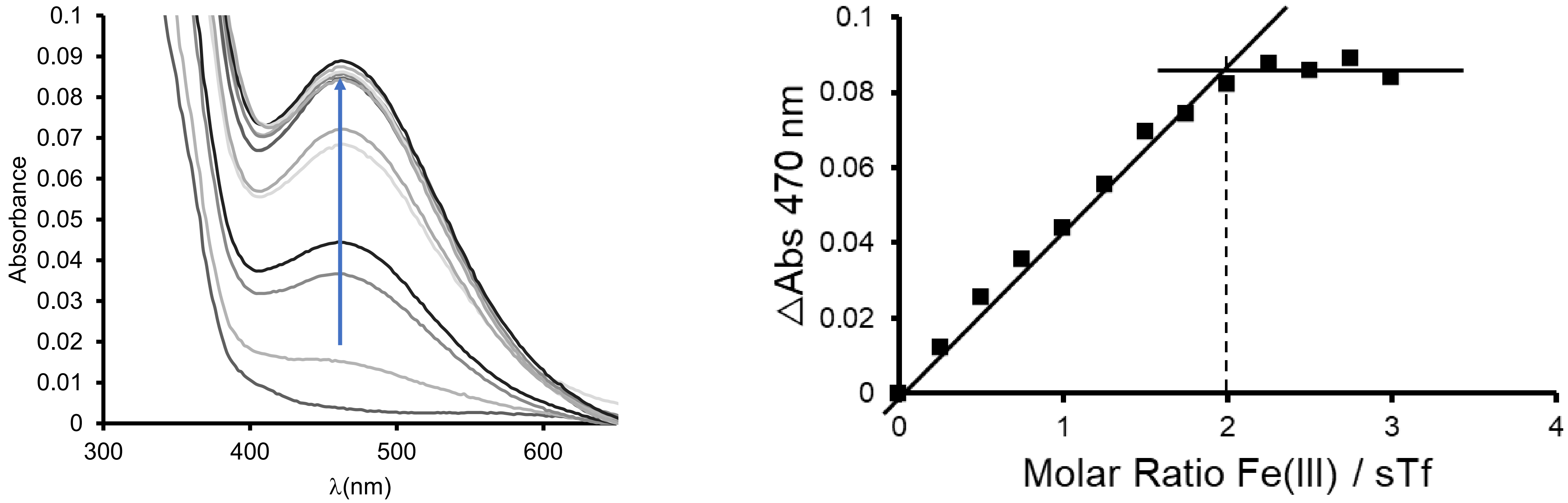

Representative stoichiometric curve for $Fe(III)_2$-sTf binding. Constructed by monitoring the LMCT absorbance growth at 470 nm.

B. Run a UREA Gel to measure sTf stability in the presence and absence of Fe(III).

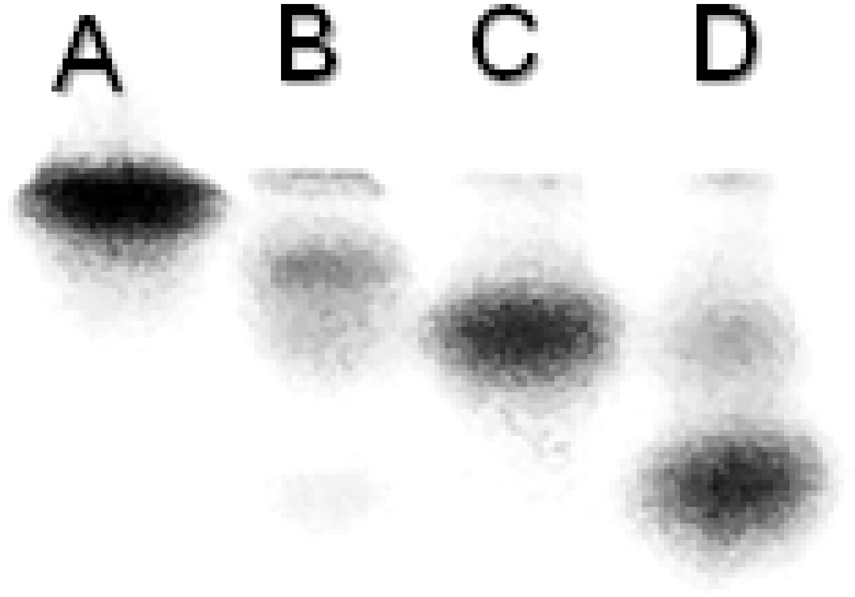

A: apo sTf

B: Fe_C sTf

C: **Fe_N sTf**

D: **Fe_2 sTf**

Representative Urea gel to monitor aposTf and different Fe(III)-bound sTf.

C. Familiarize yourself with the PyMOL application and learn the different methods and add-ons you can use to generate a crystallized protein structure.

- PyMOL is an open source molecular visualization system that produces high-quality 3D images of proteins, nucleic acids, small molecules, electron densities, surfaces, and trajectories.

- You will learn how to create a high quality image of Fe(III)-bound sTf with PyMOL using a published structure. We will download this structure from The Research Collaboratory for Structural Bioinformatics Protein Data Bank (RCSB PDB).

- You will have to download structure 3QYT from the RCSB PDB site, presented in the figure to the right. In this structure, Fe(III) is bound in a closed conformation in the C-lobe binding site and in an open conformation in the N-lobe binding site.

- To complete this part of the experience you must download the PyMOL program and bring your laptop to the laboratory session.

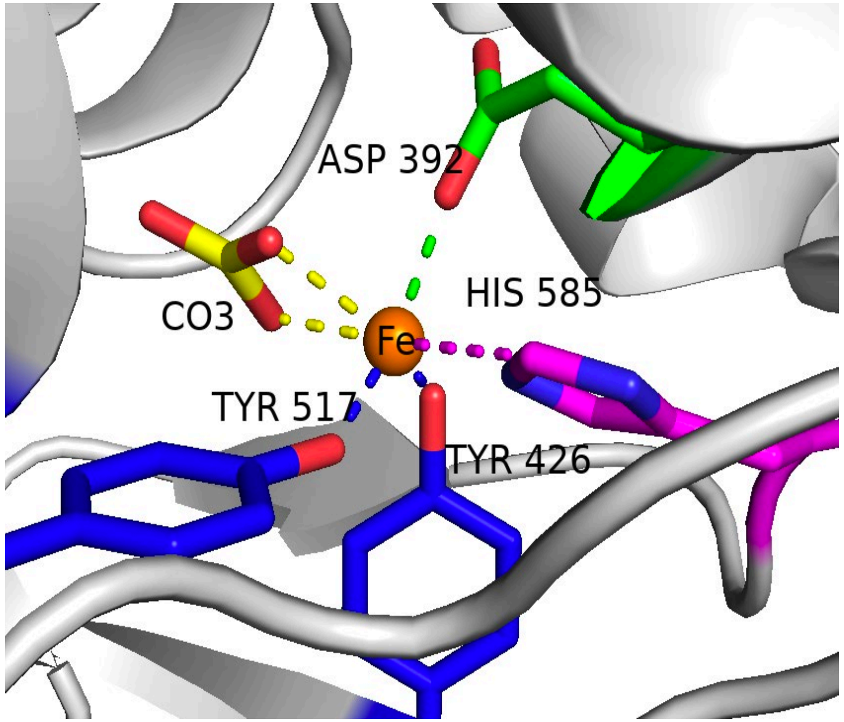

Supplement: Supplementary file 5 — ed3c01016_si_005.pdf [file ed3c01016_si_005.pdf]
