# Supplementary material for: Reinforcing Protein Biochemistry: A Two-Week Experiment Studying Iron(III) Binding by the Transferrin Protein through Stoichiometric Determination, Stability Analysis, and Visualization of the Binding Site
Source: J Chem Educ. 2024 Mar 26;101(4):1656–64. doi: 10.1021/acs.jchemed.3c01016 (PMC11033862; doi:10.1021/acs.jchemed.3c01016)
Supplement: Supplementary file 6 — ed3c01016_si_006.pdf [file ed3c01016_si_006.pdf]

Supporting Information

Reinforcing Protein Biochemistry: A Two-Week Experiment Studying Iron(III) Binding by the Transferrin Protein through Stoichiometric Determination, Stability Analysis, and Visualization of the Binding Site

Josué A. Benjamín-Rivera[1,ǂ] Mariela Pérez Otero[2,ǂ] Arthur D. Tinoco[1*]

[1]Department of Chemistry, University of Puerto Rico, Río Piedras Campus, Río Piedras, Puerto Rico 00931, United States.

[2]Department of Biology, University of Puerto Rico, Río Piedras Campus, Río Piedras, Puerto Rico 00931, United States.

[ǂ] Equal contribution

* Email: atinoco9278@gmail.com

Supporting Information F

PyMOL Activity Slides

Table of Content

	Page
Downloading PyMOL	S4-S8
Objective of the PyMOL activity	S9
Instructions for the PyMOL activity	S10-S41

Using Pymol to explore Fe(III) Binding by the blood transporter transferrin

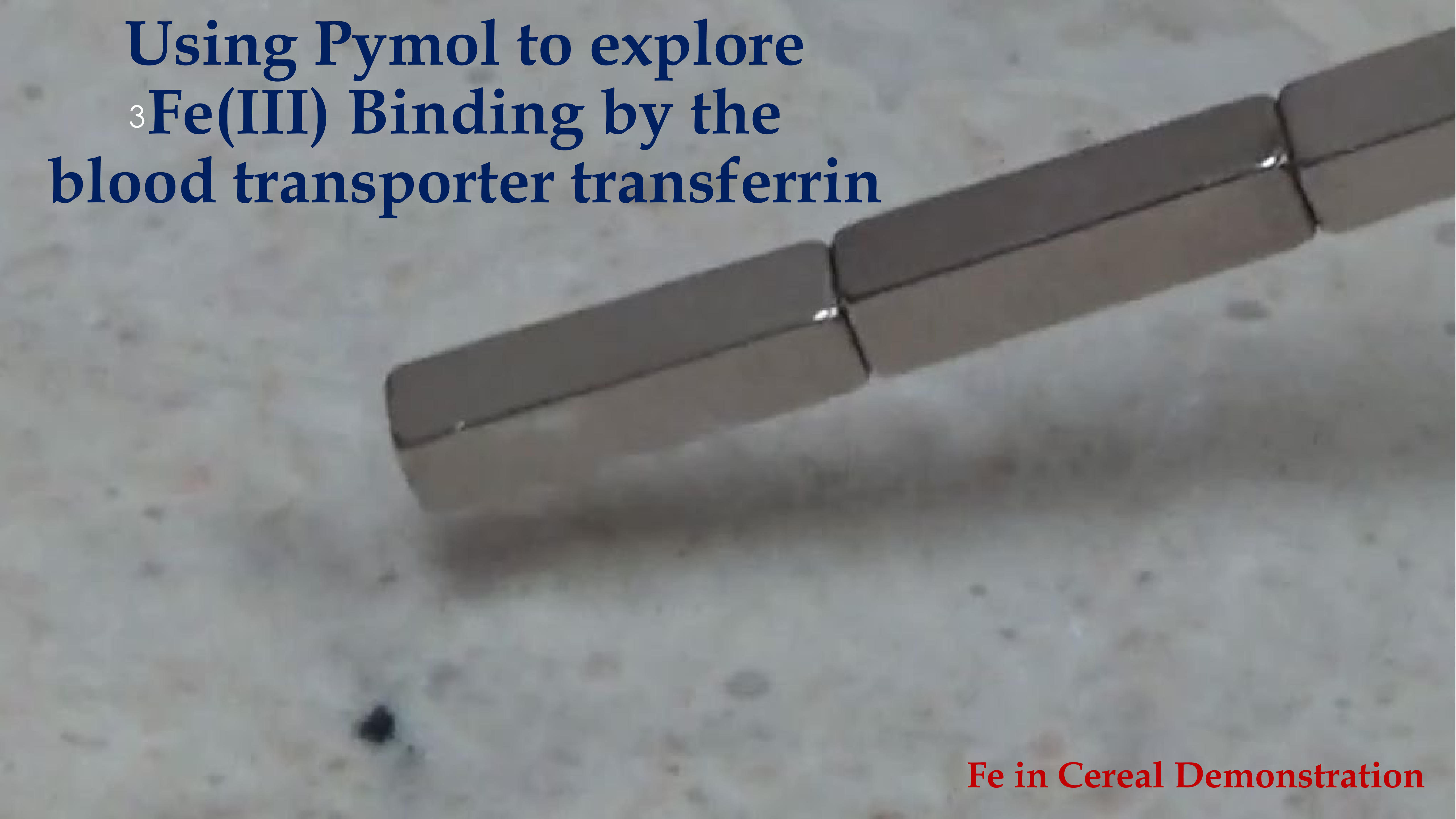

Fe in Cereal Demonstration

Request the education license for pyMOL

- https://pymol.org/edu/
- Fill the form to get a Username and password
- You will receive a email with the next step.

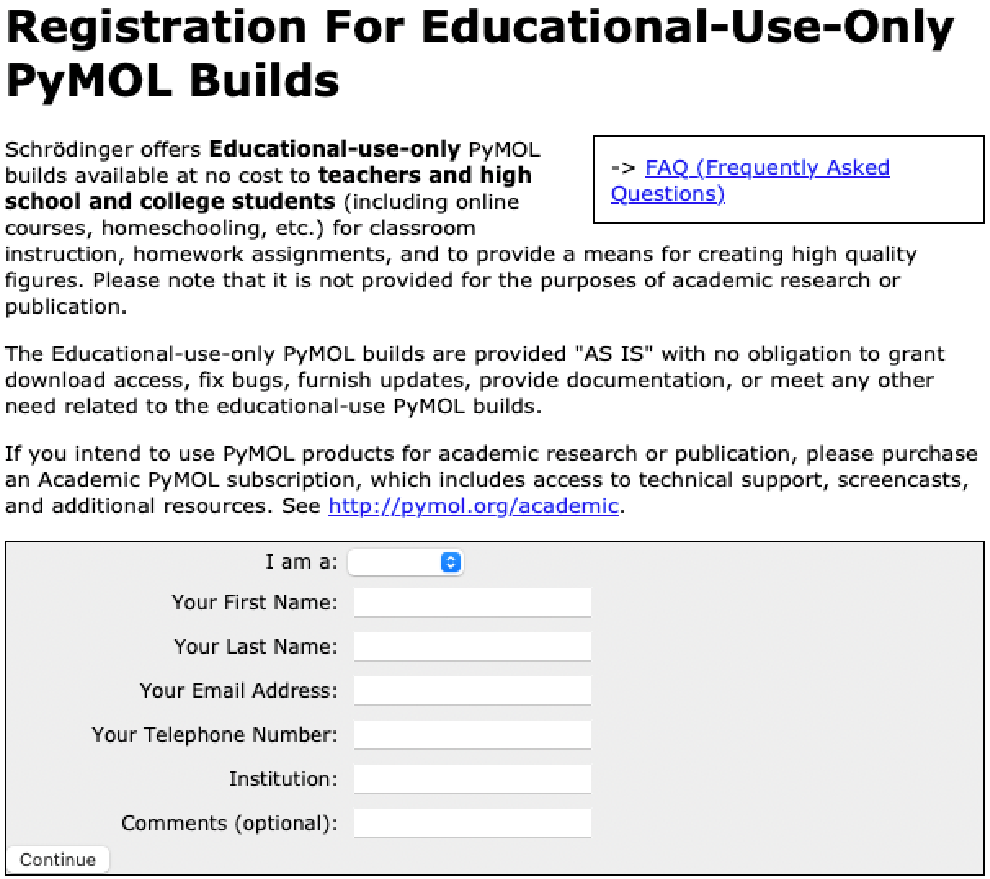

Registration For Educational-Use-Only PyMOL Builds

Schrödinger offers **Educational-use-only** PyMOL builds available at no cost to **teachers and high school and college students** (including online courses, homeschooling, etc.) for classroom instruction, homework assignments, and to provide a means for creating high quality figures. Please note that it is not provided for the purposes of academic research or publication.

-> FAQ (Frequently Asked Questions)

The Educational-use-only PyMOL builds are provided "AS IS" with no obligation to grant download access, fix bugs, furnish updates, provide documentation, or meet any other need related to the educational-use PyMOL builds.

If you intend to use PyMOL products for academic research or publication, please purchase an Academic PyMOL subscription, which includes access to technical support, screencasts, and additional resources. See http://pymol.org/academic.

I am a:
Your First Name:
Your Last Name:
Your Email Address:
Your Telephone Number:
Institution:
Comments (optional):
Continue

To download PyMOL

- DOWNLOAD URL: https://pymol.org/ep

Download Educational-Use-Only PyMOL

DO NOT SHARE THESE FILES OUTSIDE OF EDUCATIONAL ENVIRONMENTS -- they are for students and teachers only.

To the extent that you redistribute these files or the download credentials internally, please be sure that access is appropriately limited. Although primarily intended for classroom use, students, and teachers may download and use these builds on personal computers for educational tasks such as homework assignments.

PyMOL Executable Builds for Educational Use Only

The Educational-use-only PyMOL builds are provided "AS IS" with no obligation to grant download access, fix bugs, furnish updates, provide documentation, or meet any other need related to the educational-use PyMOL builds. Purchased PyMOL Academic Subscriptions with up to three years of maintenance are available to meet your longer-term educational use needs.

PyMOL 2.0 (September 2017)

License File: pymol-edu-license.lic

Installers: PyMOL Download Page

- After you enter the login in the link :
- You need to download the license File
- Go to download page to download the version compatible
  - The link bellow the license file.

PyMOL download page

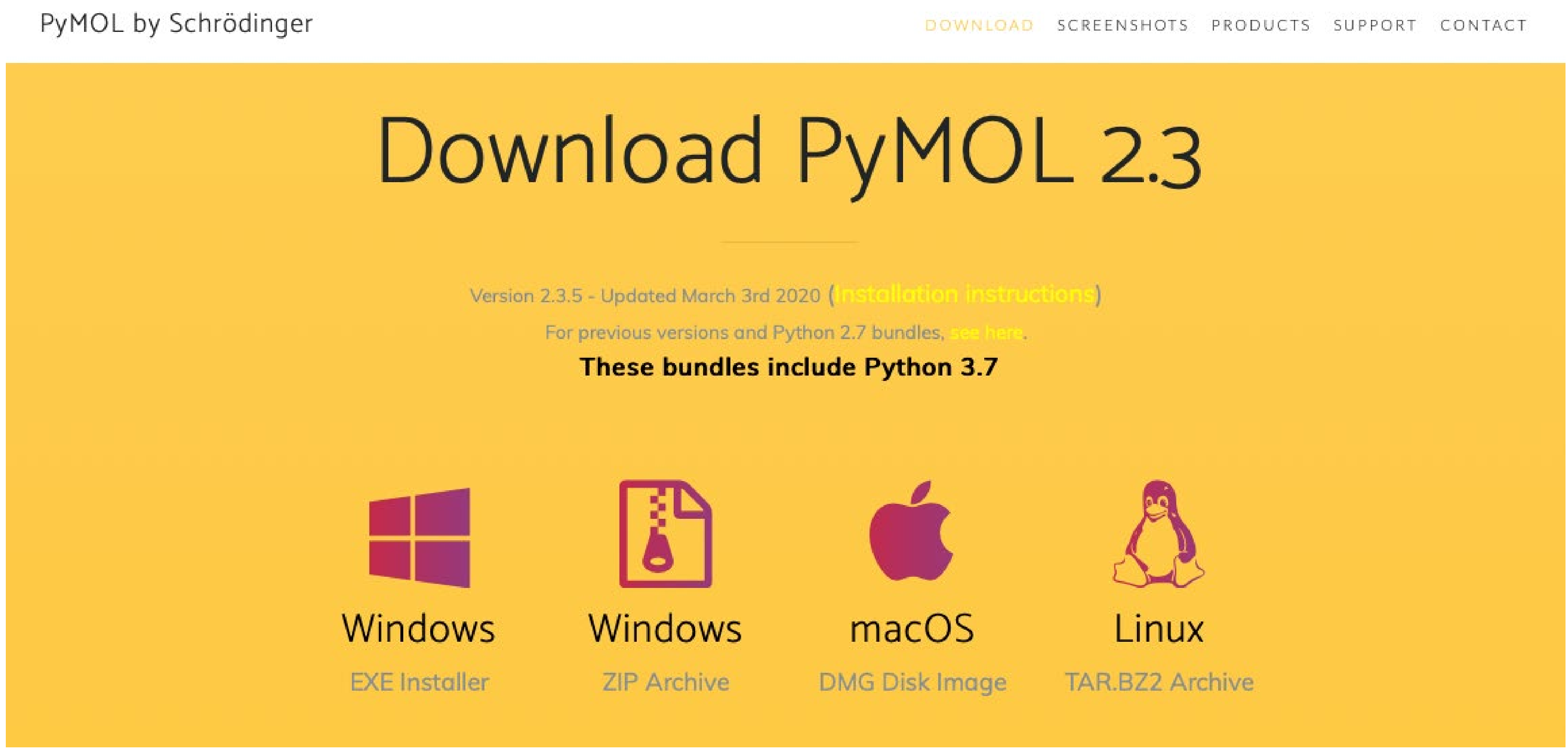

- Select the file compatible with your computer and install.
- After installation is complete, you need to activate the program using the license obtained from your previous download.

Note:

- The version of PyMOL used in the workshop is 2.3. If you download another version, some commands will be different.

What is PyMOL?

- PyMOL is an open source molecular visualization system created by Warren Lyford DeLano. It was commercialized initially by DeLano Scientific LLC, which was a private software company dedicated to creating useful tools that become universally accessible to scientific and educational communities. It is currently commercialized by Schrödinger, Inc.

- PyMOL can produce high-quality 3D images of proteins, nucleic acids, small molecules, electron densities, surfaces, and trajectories. It can also be used to make short videos to demonstrate molecular dynamics.

- According to the original author, by 2009, almost 25% of all published images of 3D protein structures in the scientific literature were made using PyMOL.

-Objective-

To identify the amino acids and synergistic anion that bind Fe(III) at the C-terminal lobe (C-lobe) of sTf

- Website: https://www.rcsb.org/

- The Research Collaboratory for Structural Bioinformatics Protein Data Bank (RCSB PDB) is the U.S. data center for the global PDB archive of 3D structural data for large biomolecules (proteins, DNA, and RNA).

- Access and download the structure **3QYT** from the RCSB PDB

PyMOL

Upper control panel

Command line

Display area

Command line

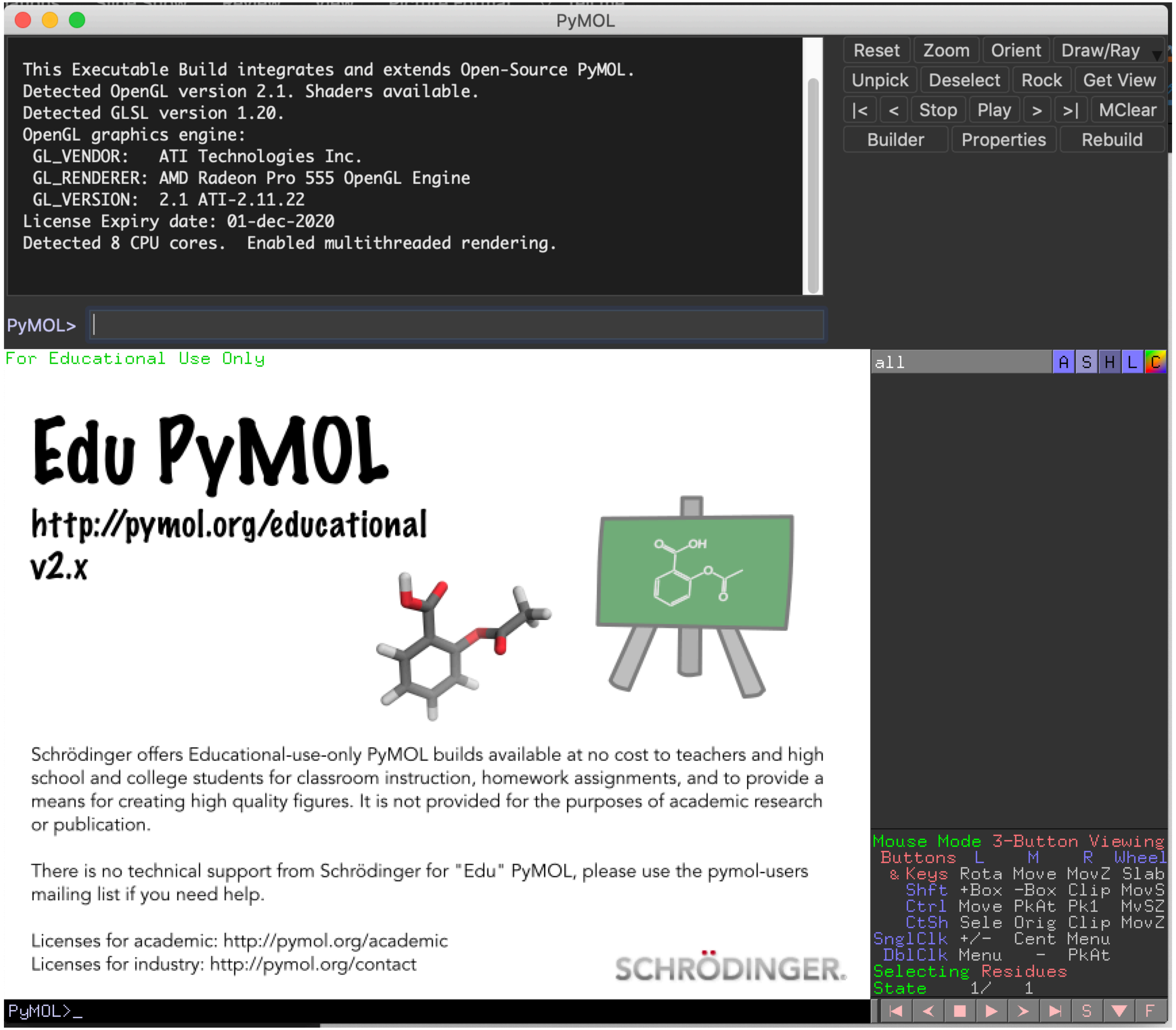

Object menu panel

Mouse controller

Movie controller

How to open a PDB file

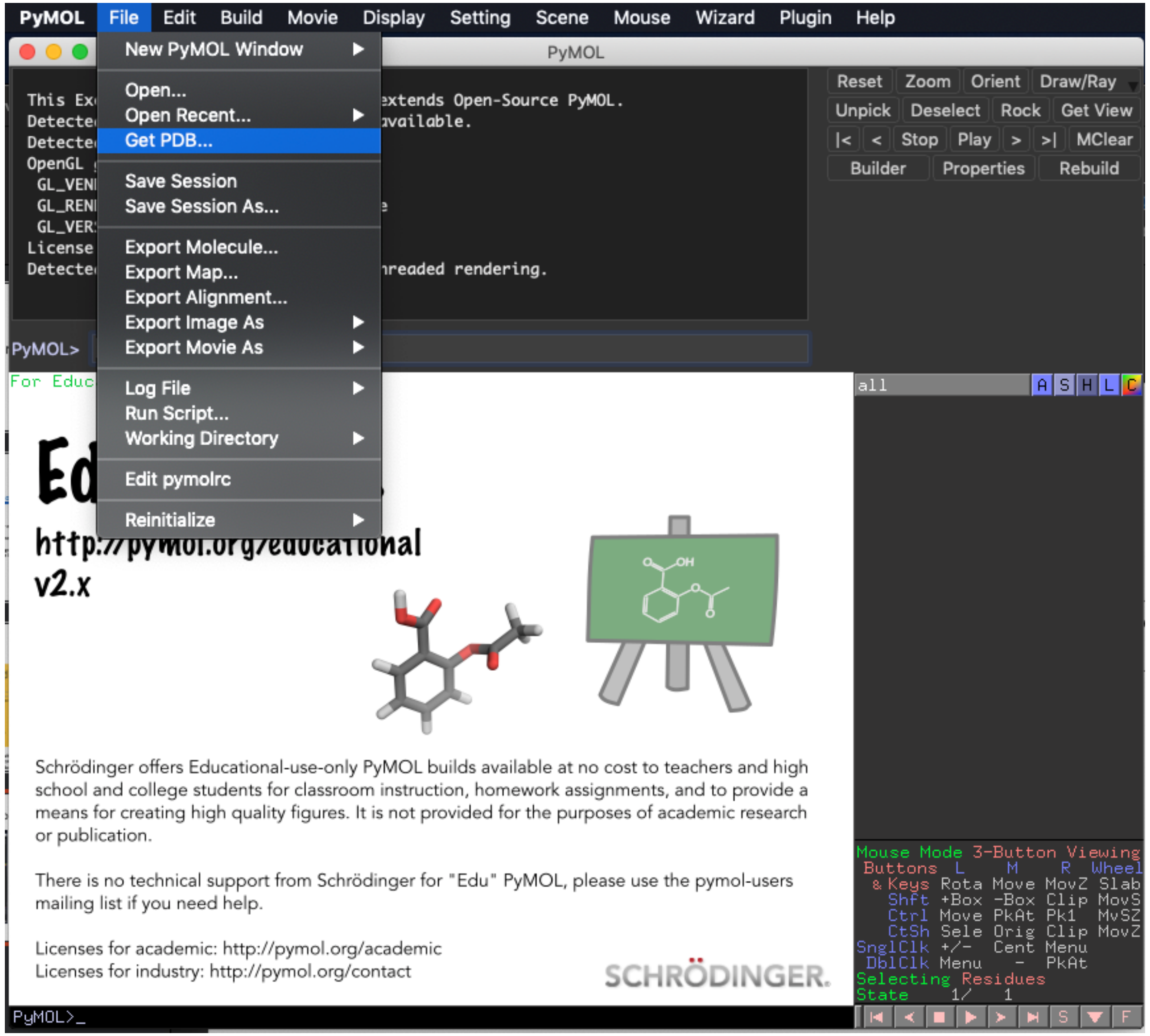

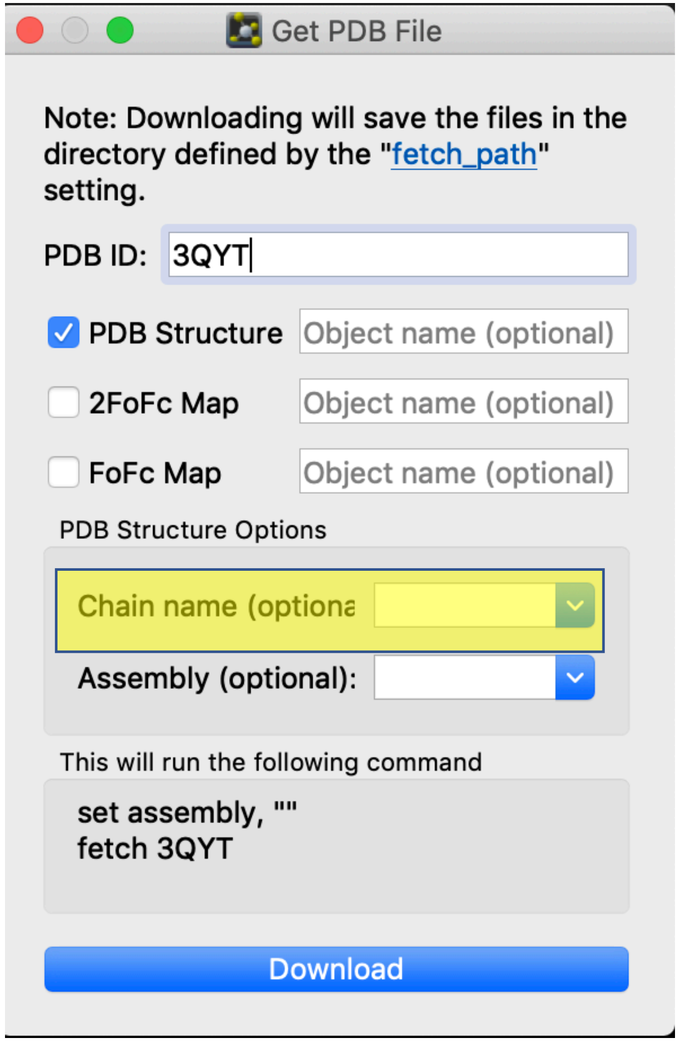

Chain name is to select only one chain if the PDB has multiple chains

After downloading, you get the image below.

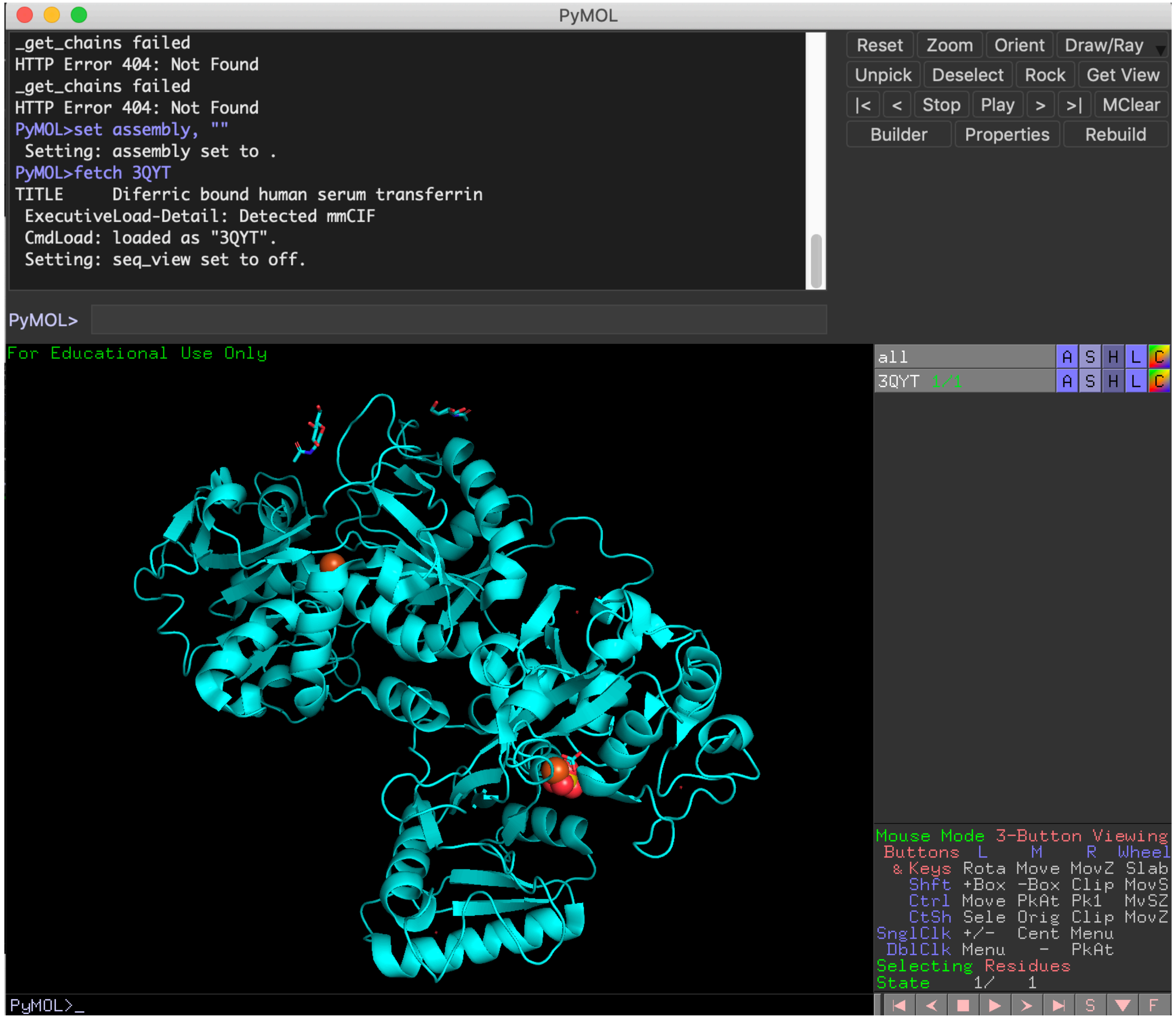

To see all of the amino acids

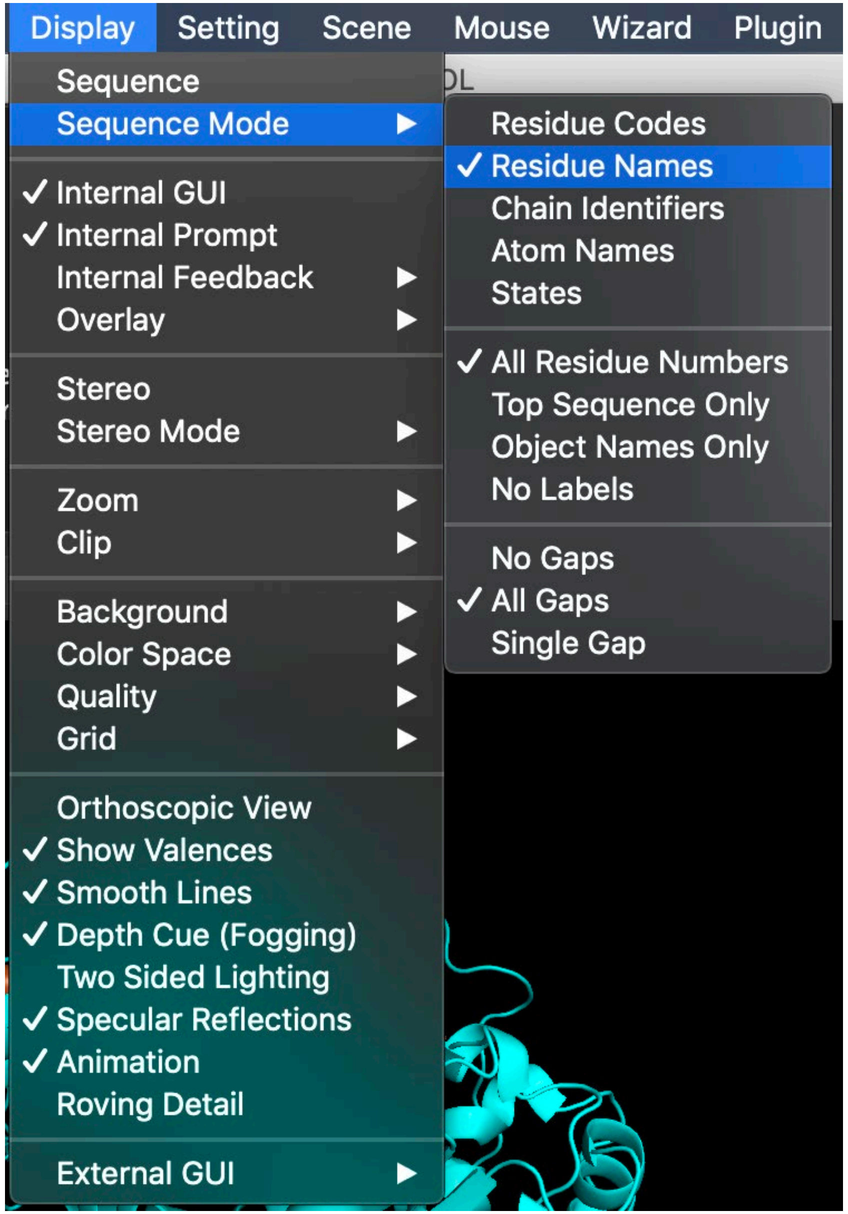

First step : Display : Sequence Mode : Residue Names

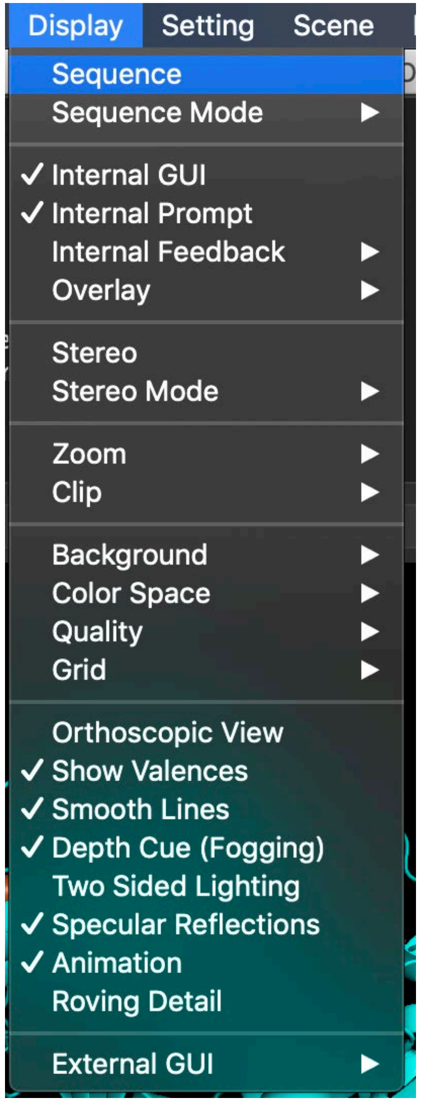

Second step : Display : Sequence

Sequence line

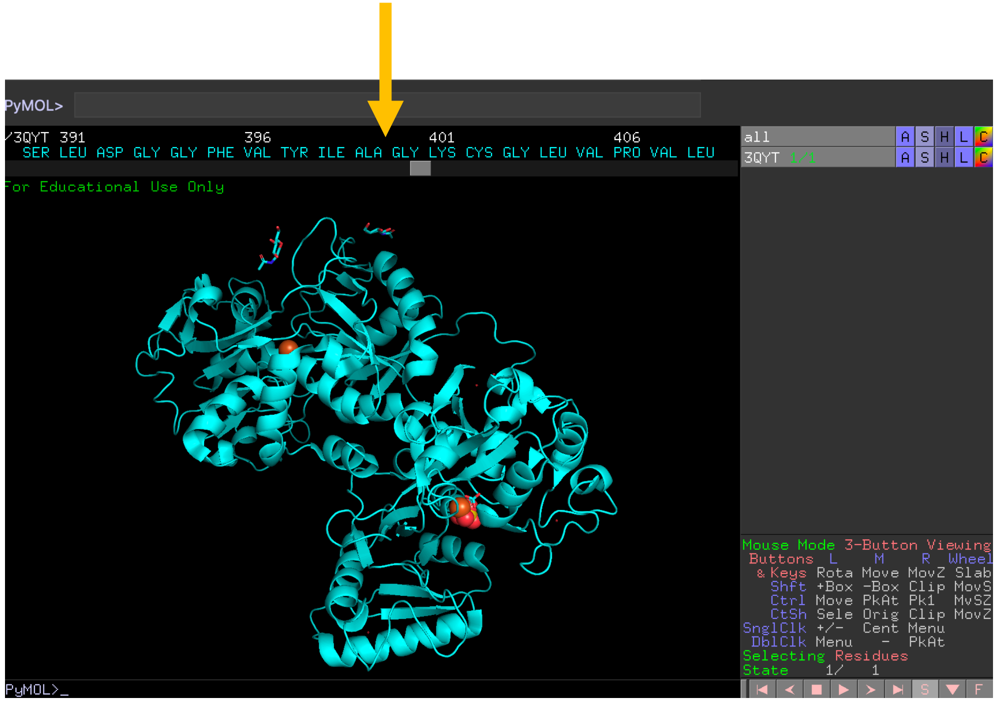

Note: The sequence line will help to identify any component in the PDB, like amino acids, small molecules, and metal ions.

The protein amino acid positions in terms of the two lobes

N-lobe:

N1 Domain: 1-92 and 247-330

N2 Domain: 93-246

C-lobe:

C1 Domain: 340-425 and 573-679

C2 Domain: 426-572

To illustrate the amino acids and synergistic anion bound to Fe(III) within the C-lobe of STf

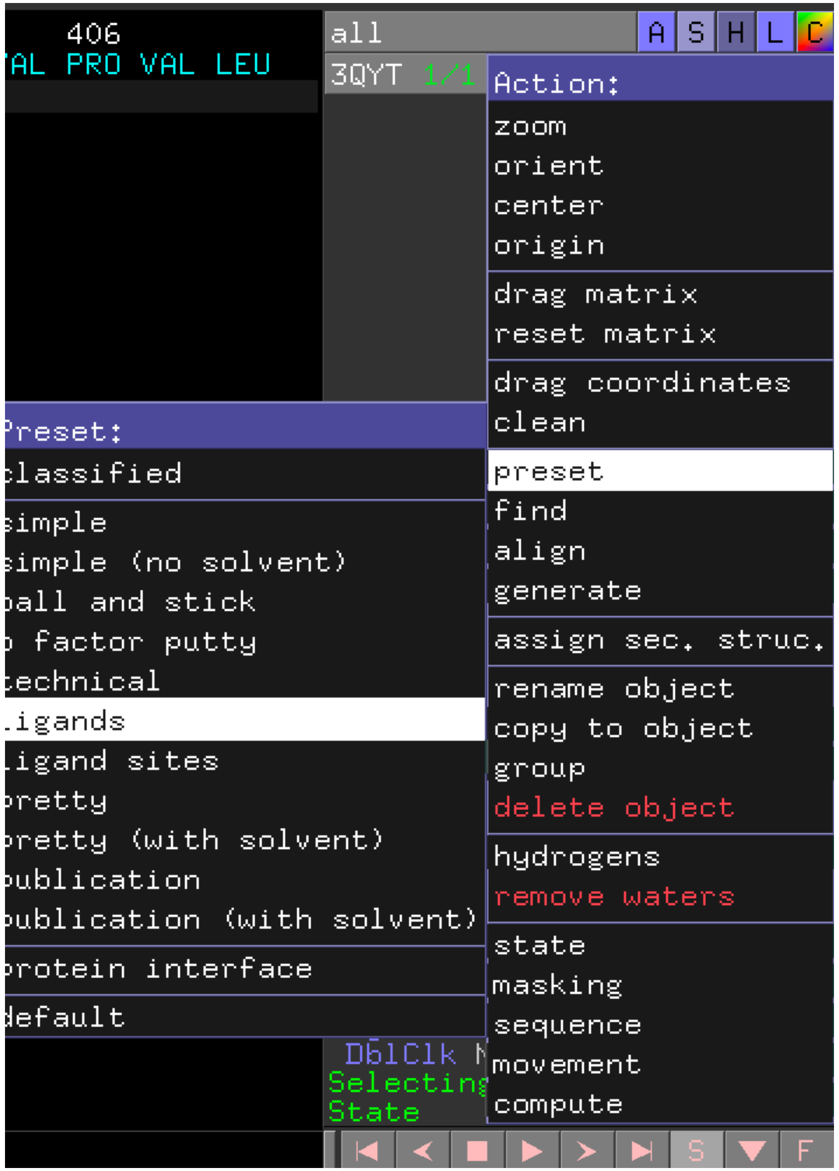

In the **object menu panel**
Click the **A** (Action command)

Click **Preset: Ligands**

This action reveals the two metal binding sites and the interactions with the metal ion. The rest of the protein will appear as a ribbon.

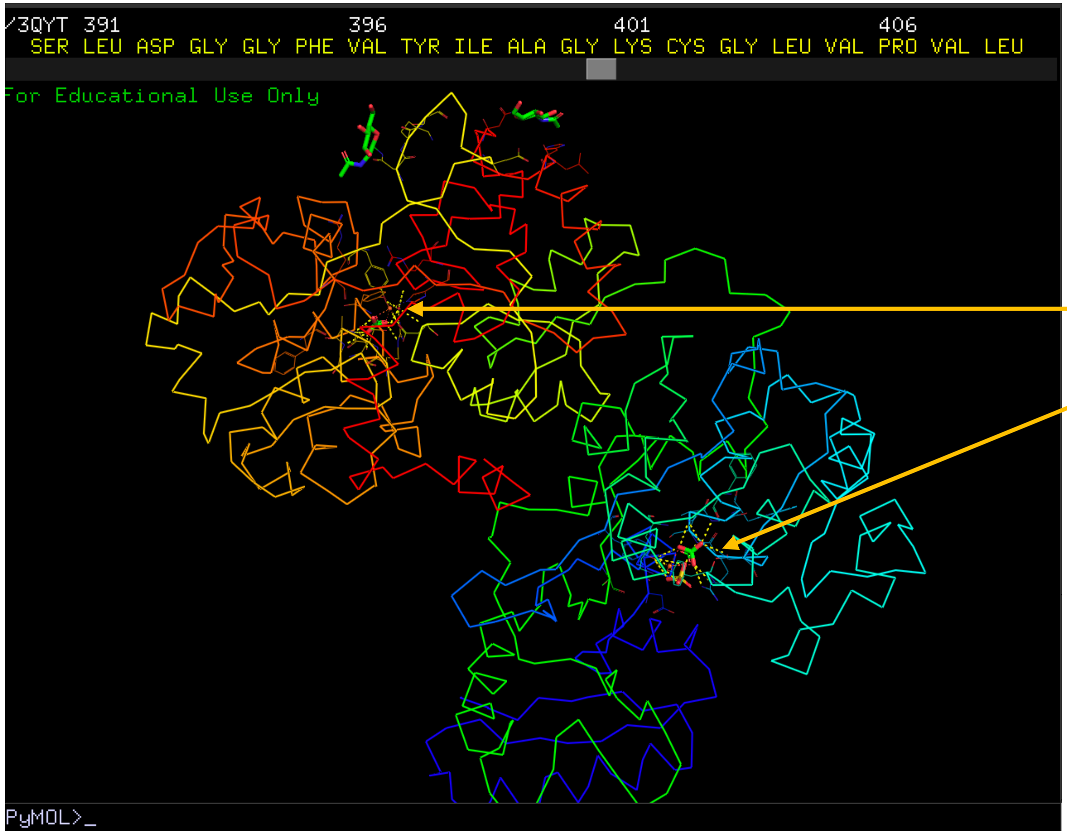

The two binding sites

To display only the interactions within the metal binding site.

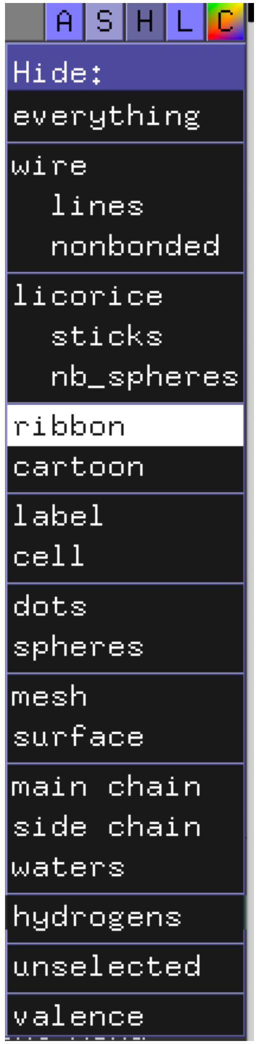

In the **object menu panel**
Click the **H** (Hide command)

Click **ribbon**

Only the binding sites will remain.

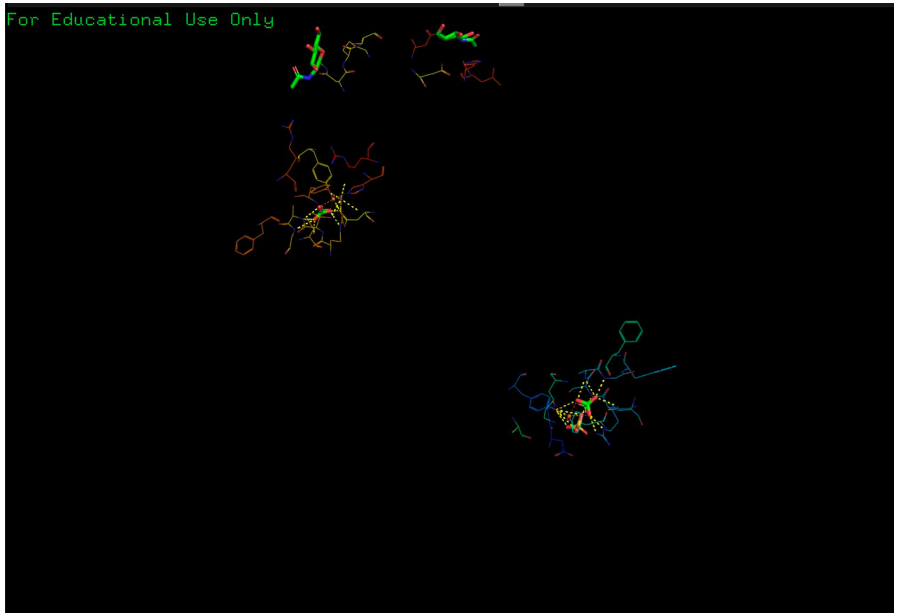

Zoom into one of the binding sites.

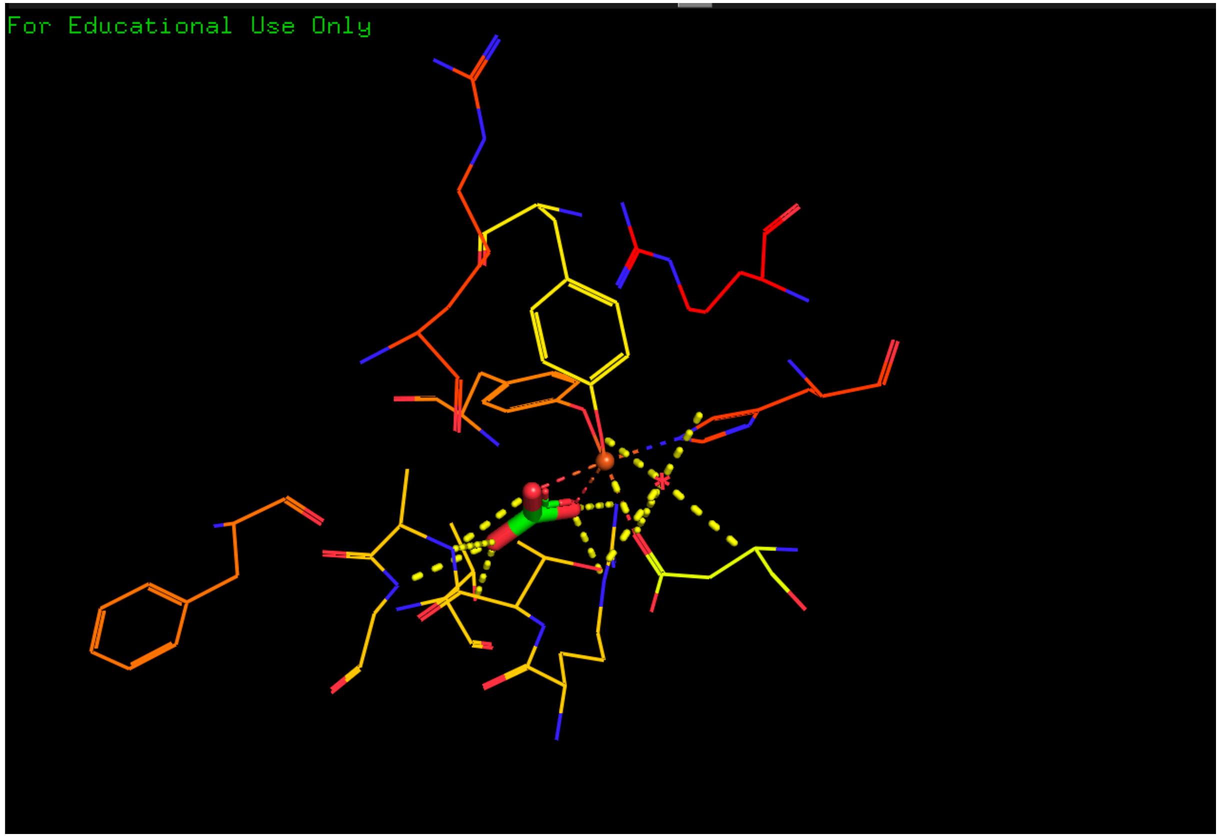

To reveal the coordination details of Fe(III)

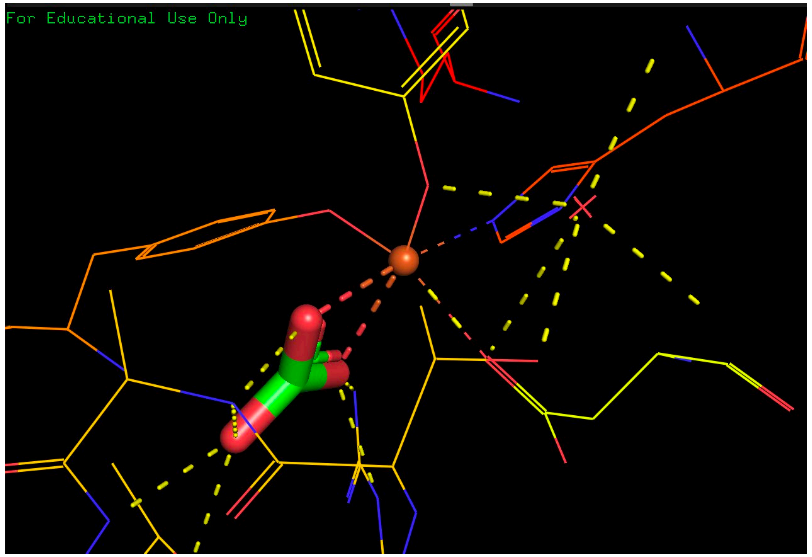

The orange sphere is the Fe(III). The lines that touch the sphere represent the metal bonds to the amino acids and the synergist anion carbonate.

Note: The coordination number of Fe(III) is 6

- Bound to four amino acids
- Bound to carbonate in a bidentate mode

To identify the amino acids bound to Fe(III)

To identify the amino acids, click each amino acid that directly interacts with Fe(III)

Example: The molecule that is clicked is the one in orange highlight.

To see the identification:

A. Note the text that is displayed in the upper control panel

B. Look at the amino acid marked in the sequence line.

What is the identification of the amino acid?

TYR 517

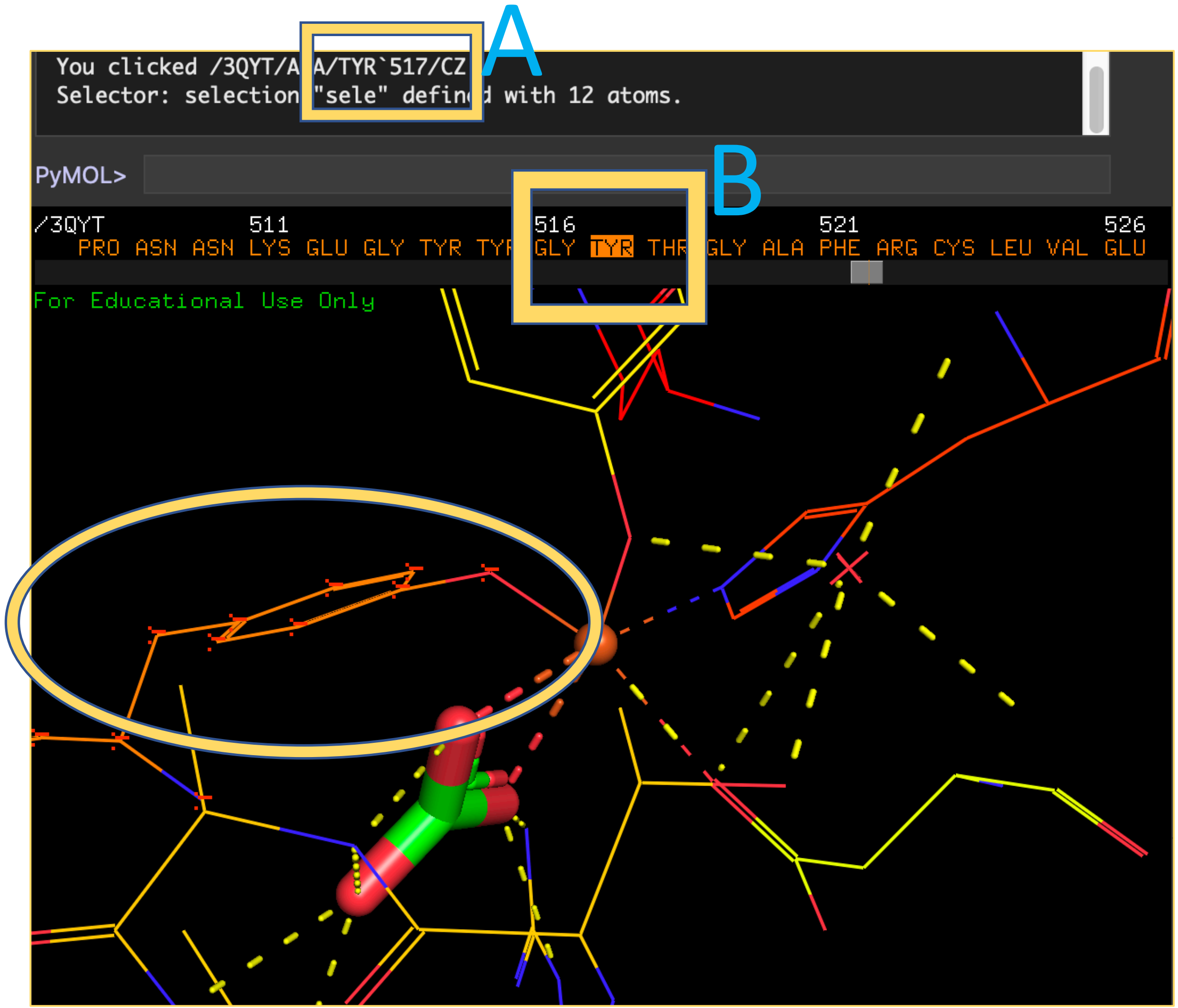

Prepare a table of the two metal binding sites identifying the bound amino acids and synergistic anions

C-lobe Site		N-lobe Site	
AA or Anion	Metal bond	AA or Anion	Metal bond
HIS 585	1	TYR 95	1
TYR 517	1	TYR 188	1
TYR 426	1	CO_3^{2-}	2
ASP 392	1	SO_4^{2-}	2
CO_3^{2-}	2		

-Objective-

Preparing a high-resolution image of the Fe(III) C-lobe binding site of STf

Protein database (PDB) visualization vs PyMOL

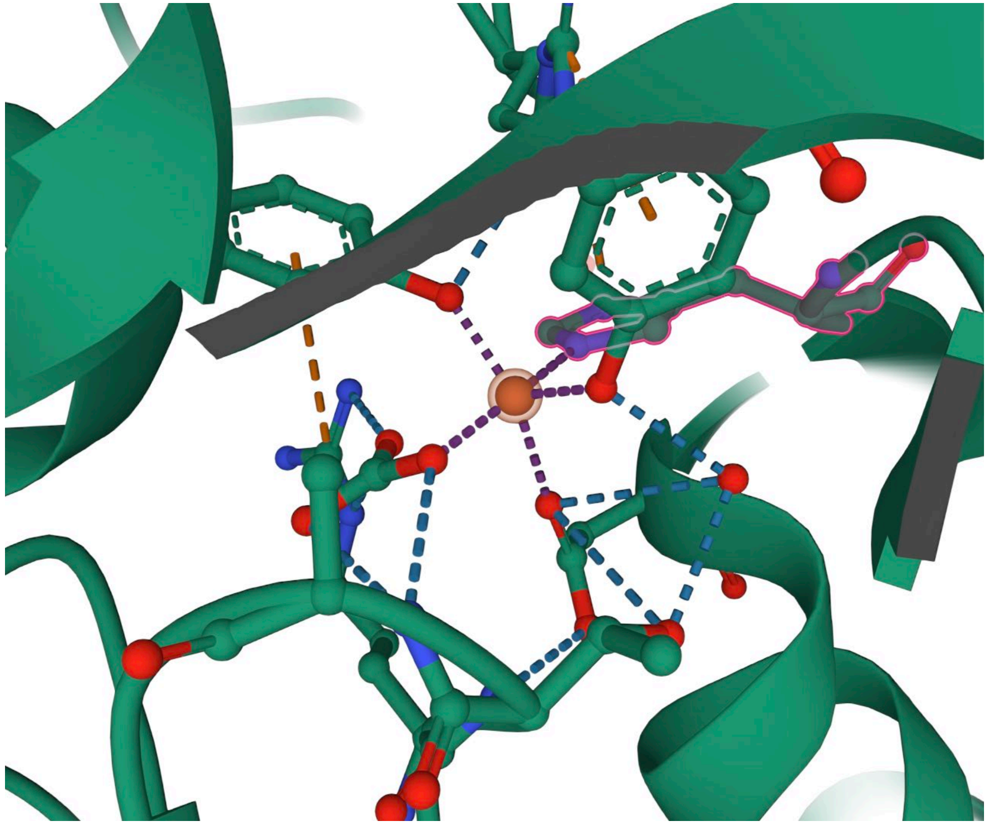

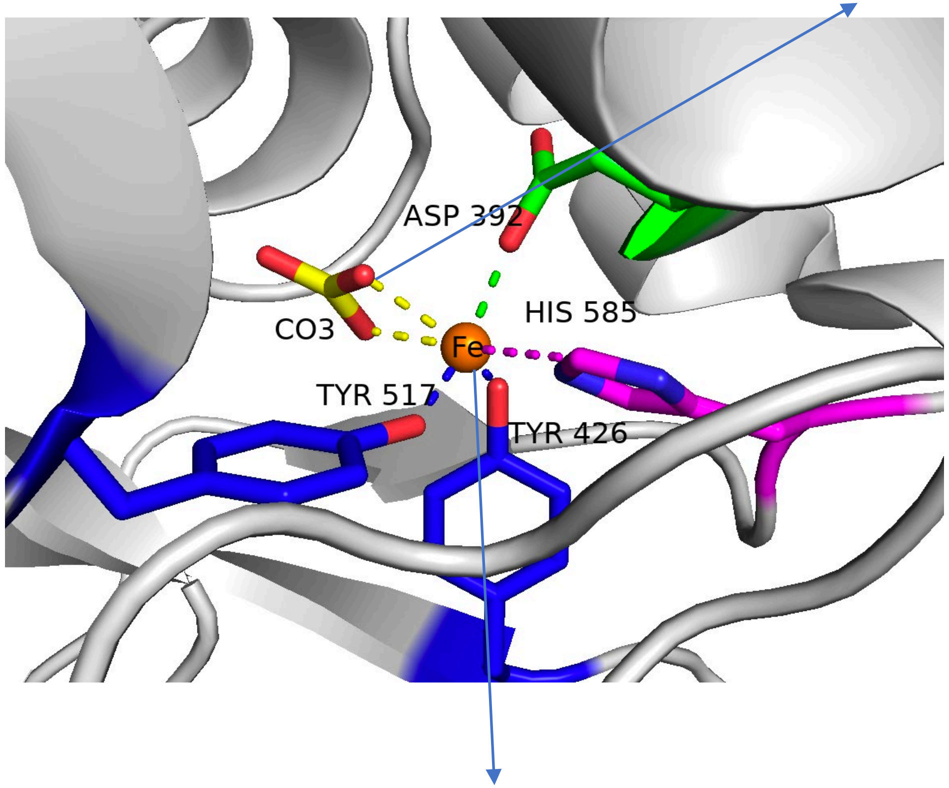

PDB 3QYT	C-site
Synergistic anions/molecules	Bidentate CO_3^{2-}
Coordination Number	6
Protein Residues	Tyr 426<br>Tyr 517<br>His 585<br>Asp 392

To begin:

- Close and reopen the program
- Download the protein as previously shown

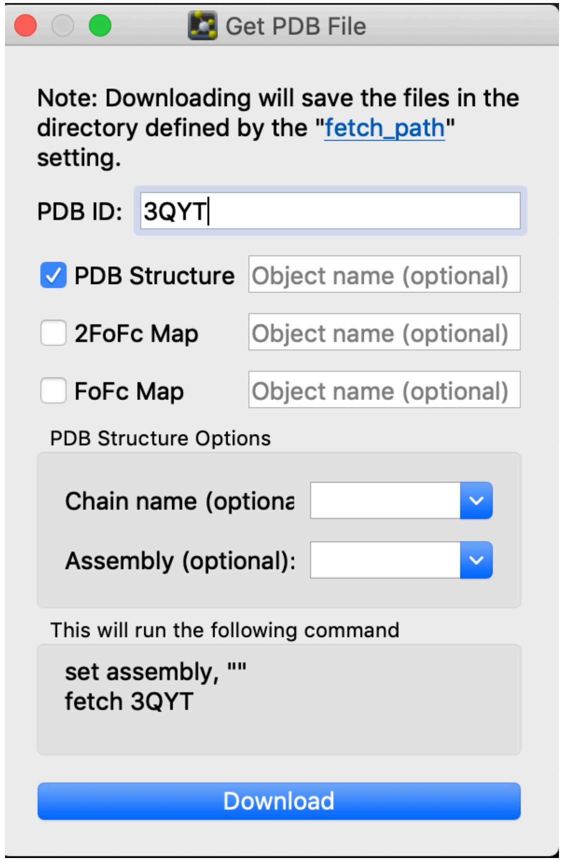

Begin by selecting the amino acids

Select **His585** from the sequence line

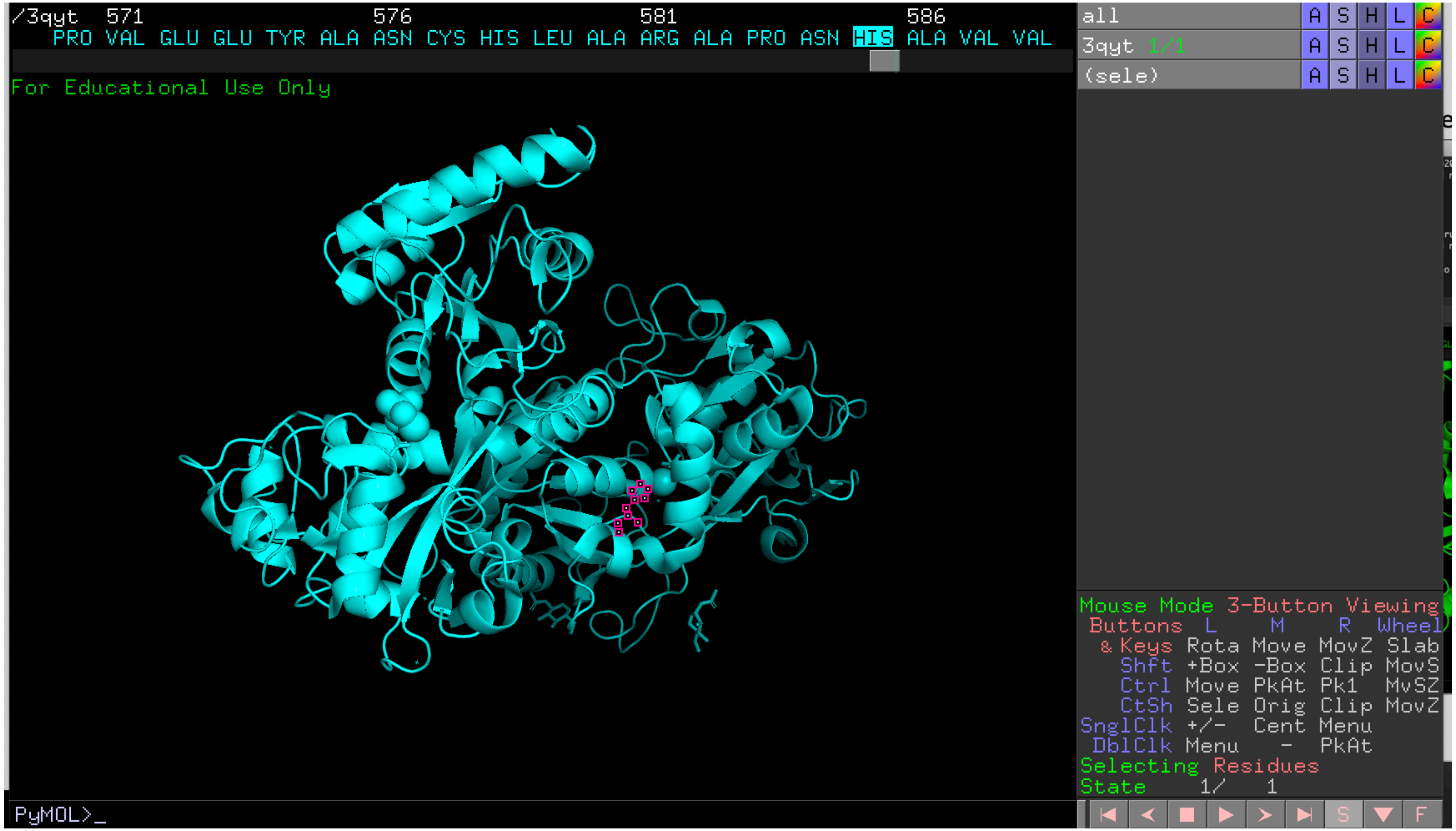

Change the name (sele) in the object menu panel

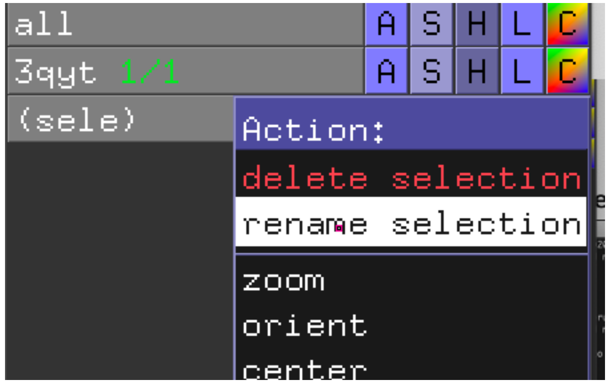

Name using the 3-letter code and protein position of His585

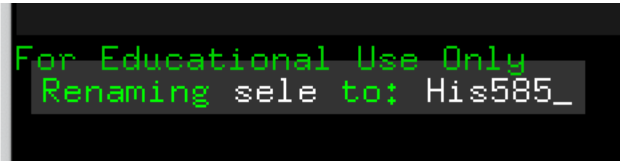

Continue selecting the remainder of the metal binding site

1. Unselect the previously selected His585

2. Follow the same protocol to select and name the rest of the metal binding site including the carbonate and Fe

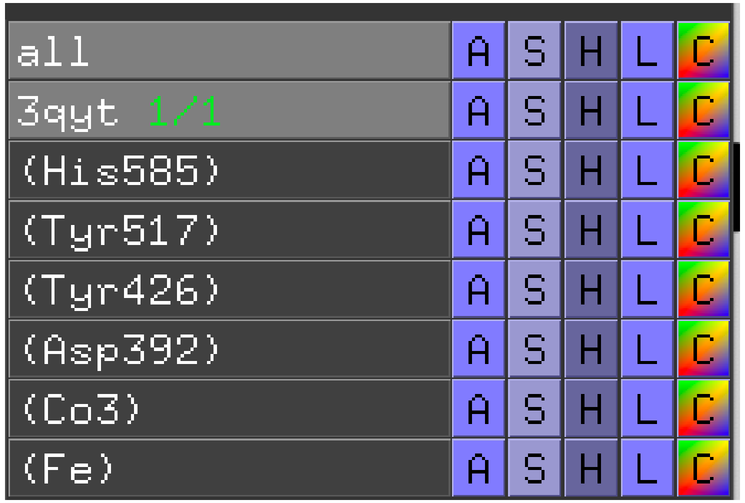

To improve the visualization

Type in the command line:

1. set cartoon_side_chain_helper, 1

   This eliminates the backbone of the amino acids

2. remove hydro

   This removes the hydrogens

3. set sphere_scale, 0.25, (all)

   This reduces the size of the sphere

To improve the visualization

In Display

1. Change the background by

   - Clicking Background
   - Clicking White

2. Remove the valences by

   - Unchecking the Show Valences

Background White

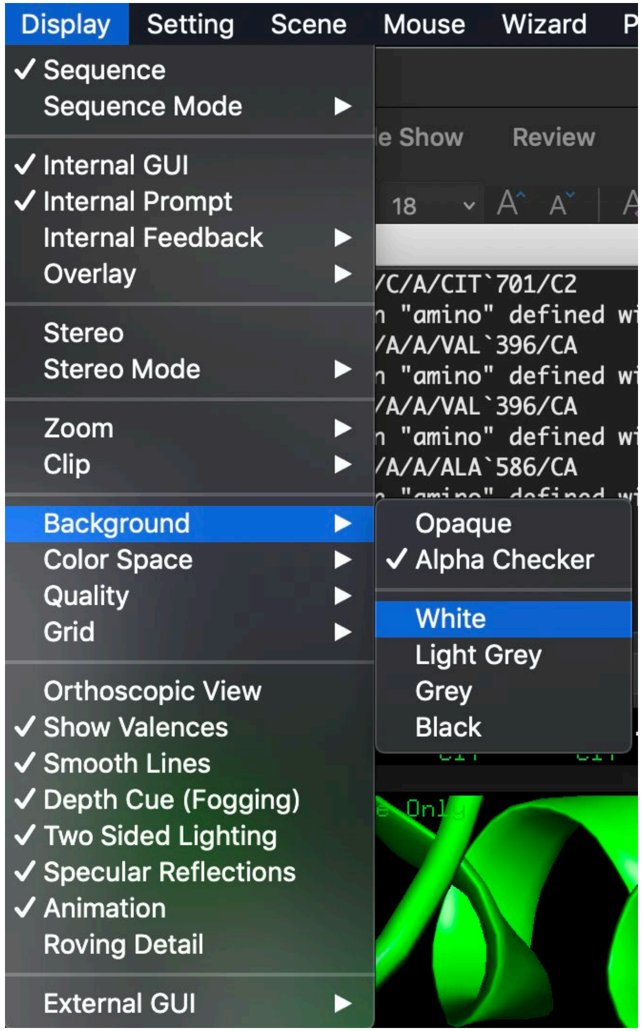

Remove valence

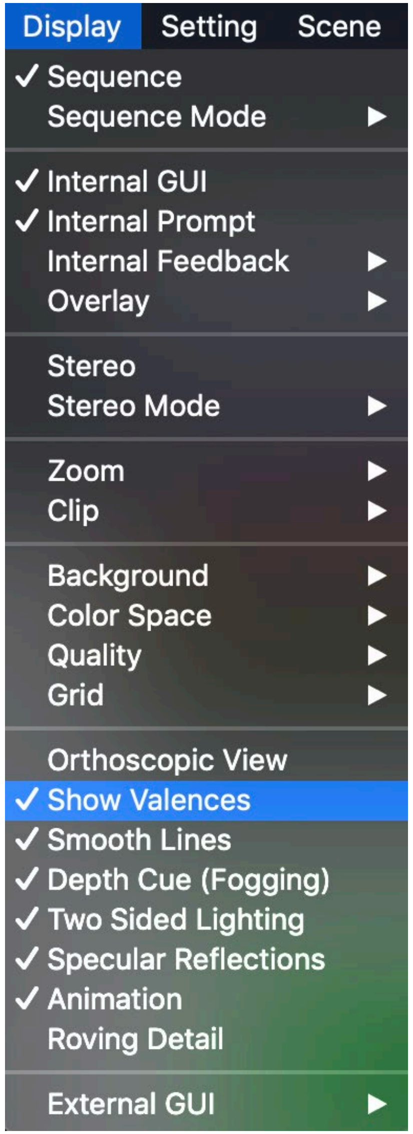

Make the protein transparent

Protein color gray 90

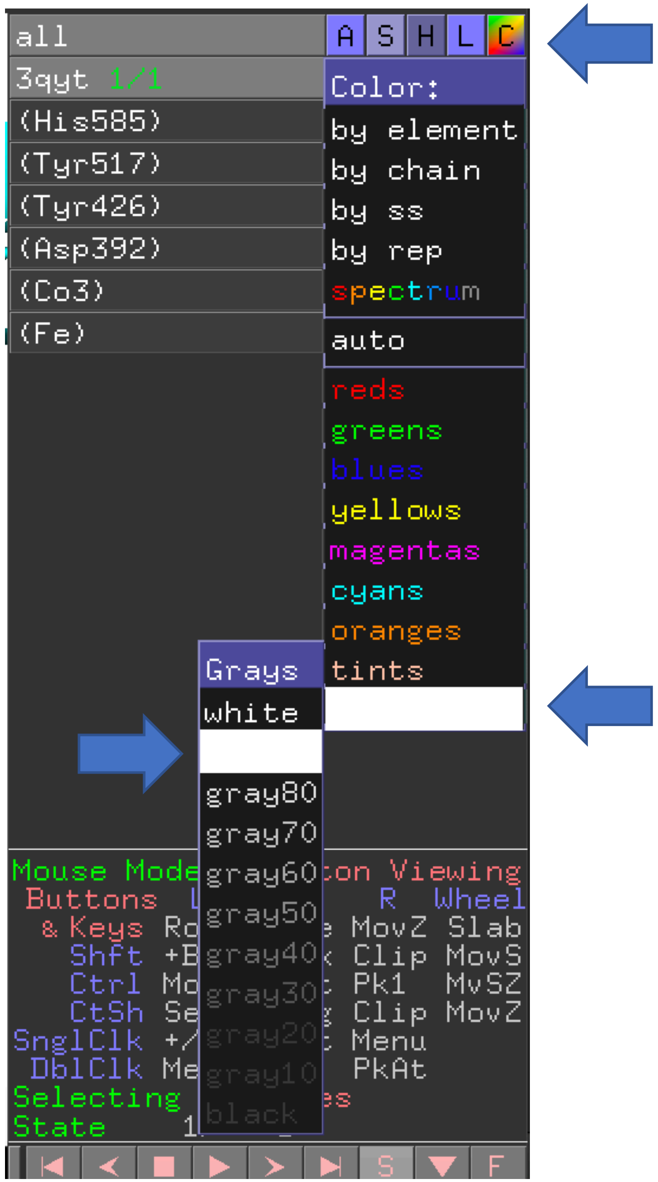

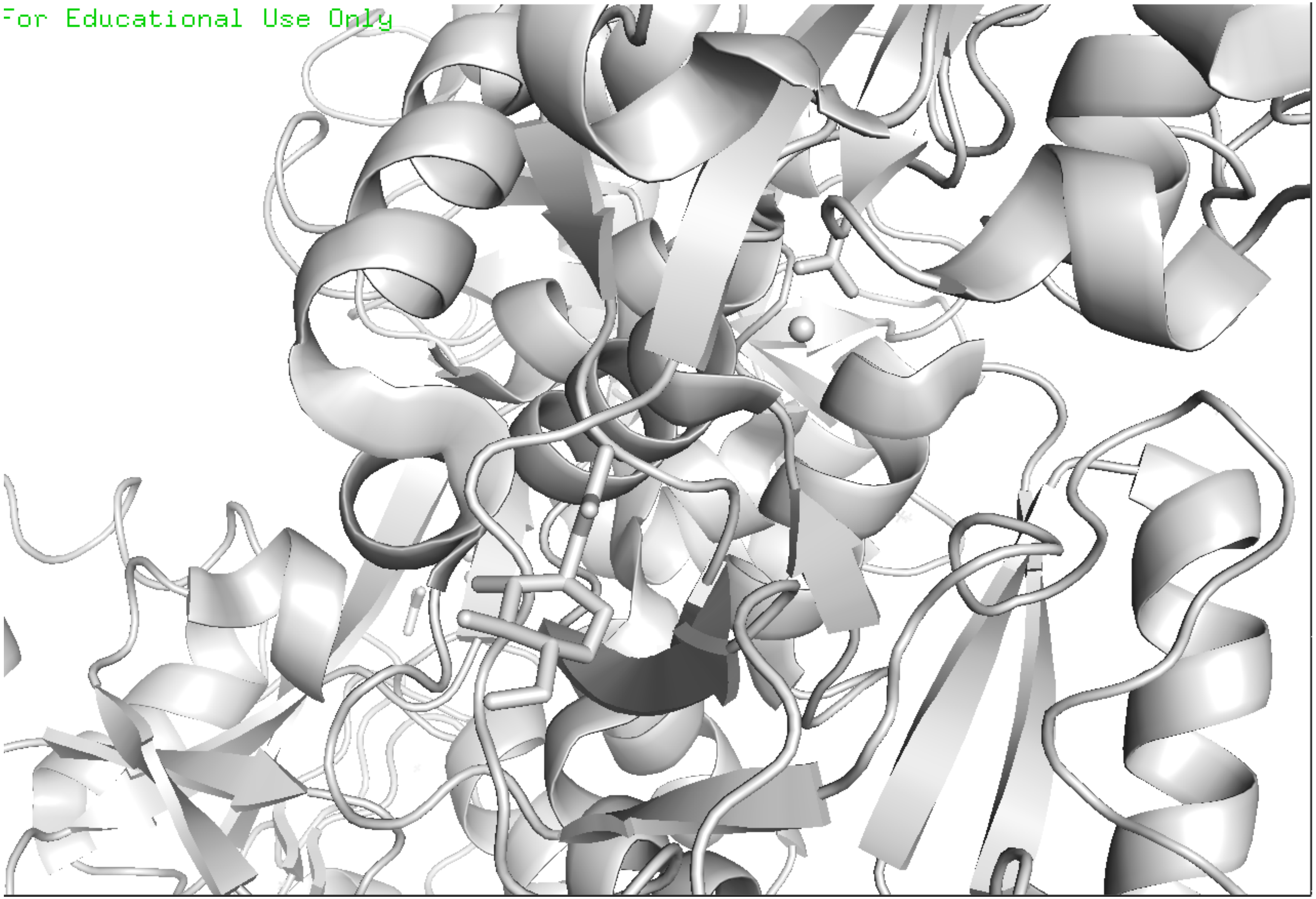

Modify the shape and color of the amino acids

Select **His585:**

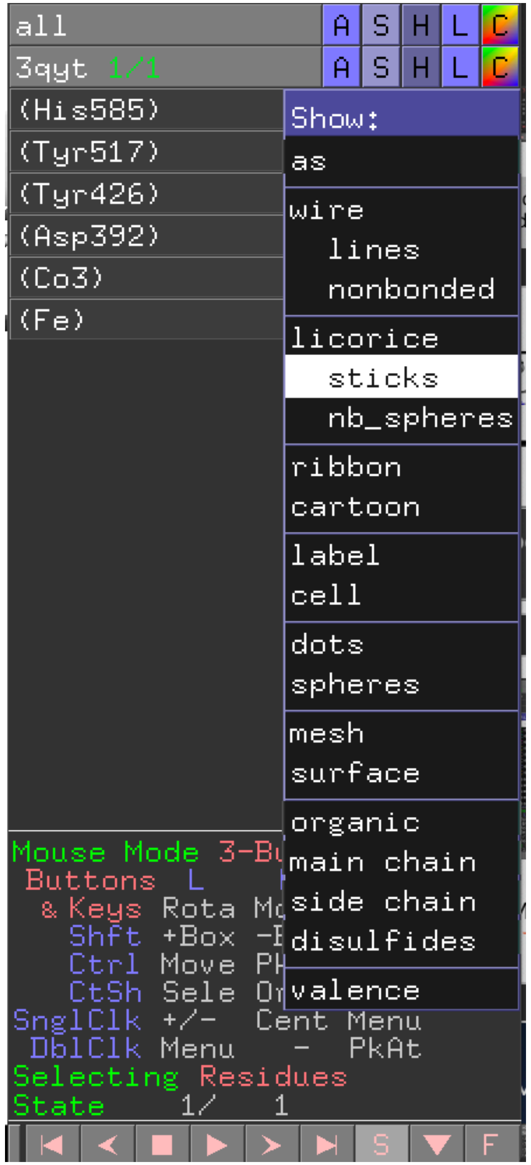

Modify the shape and color of the amino acids

Select **His585:**

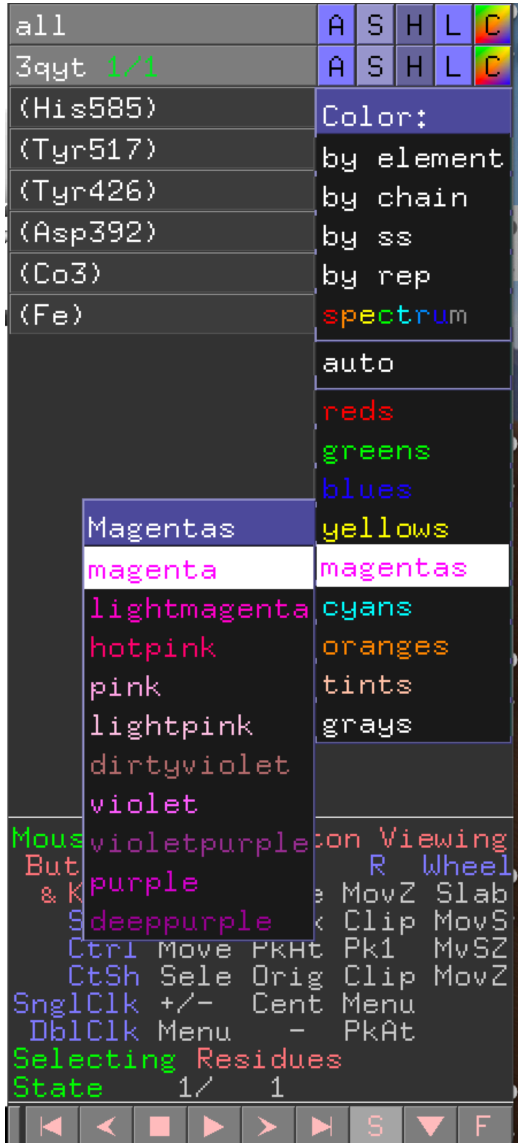

Modify the shape and color of the amino acids

Select **His585:**

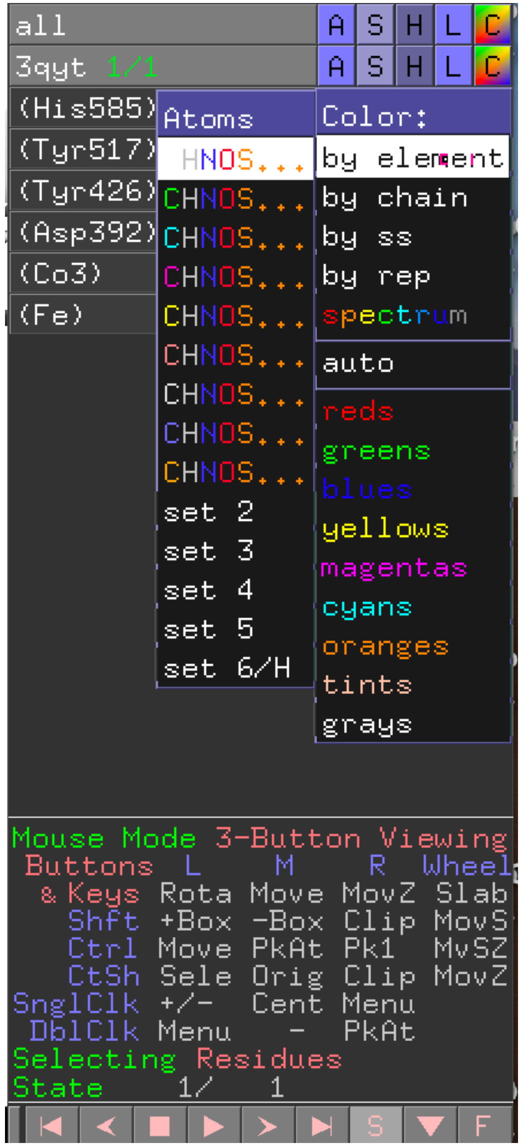

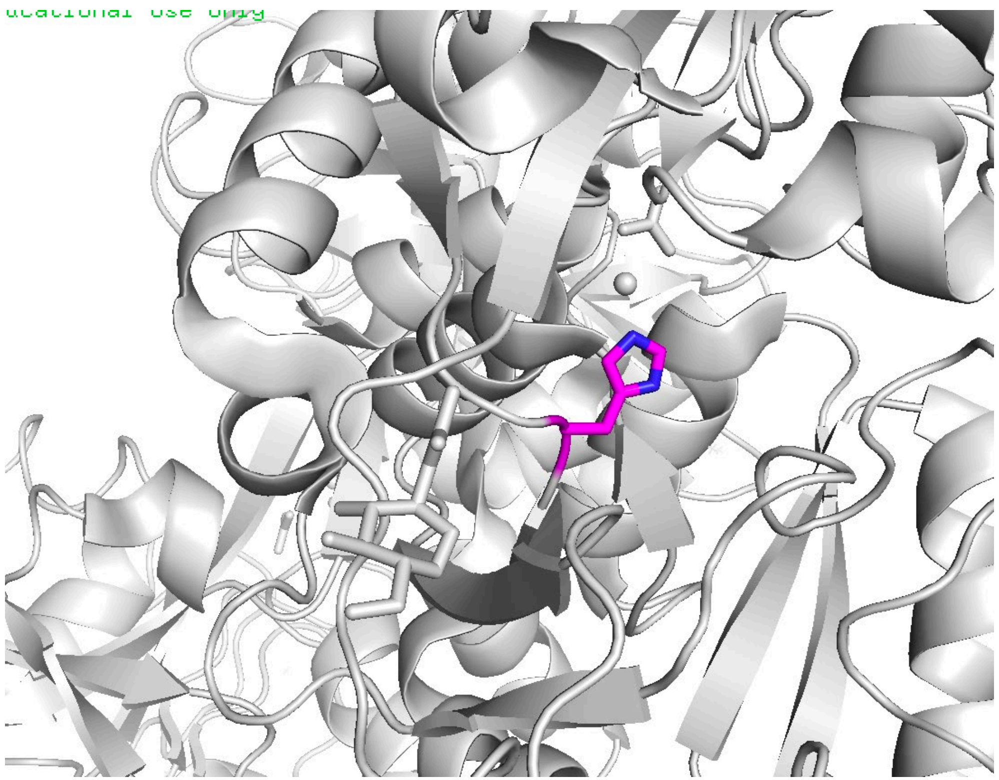

Repeat the same steps with the other parts of the metal binding site

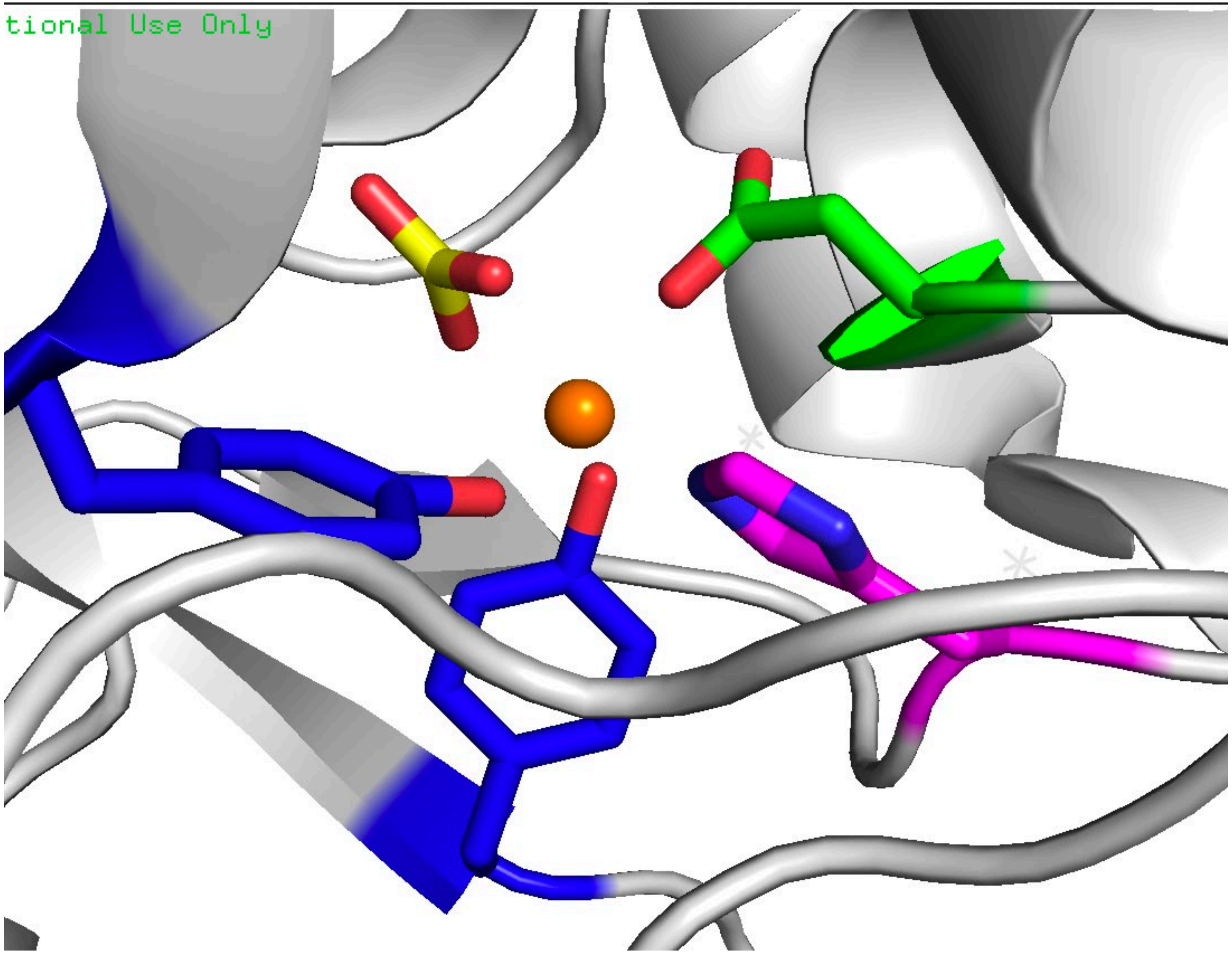

Change to 3-button editing

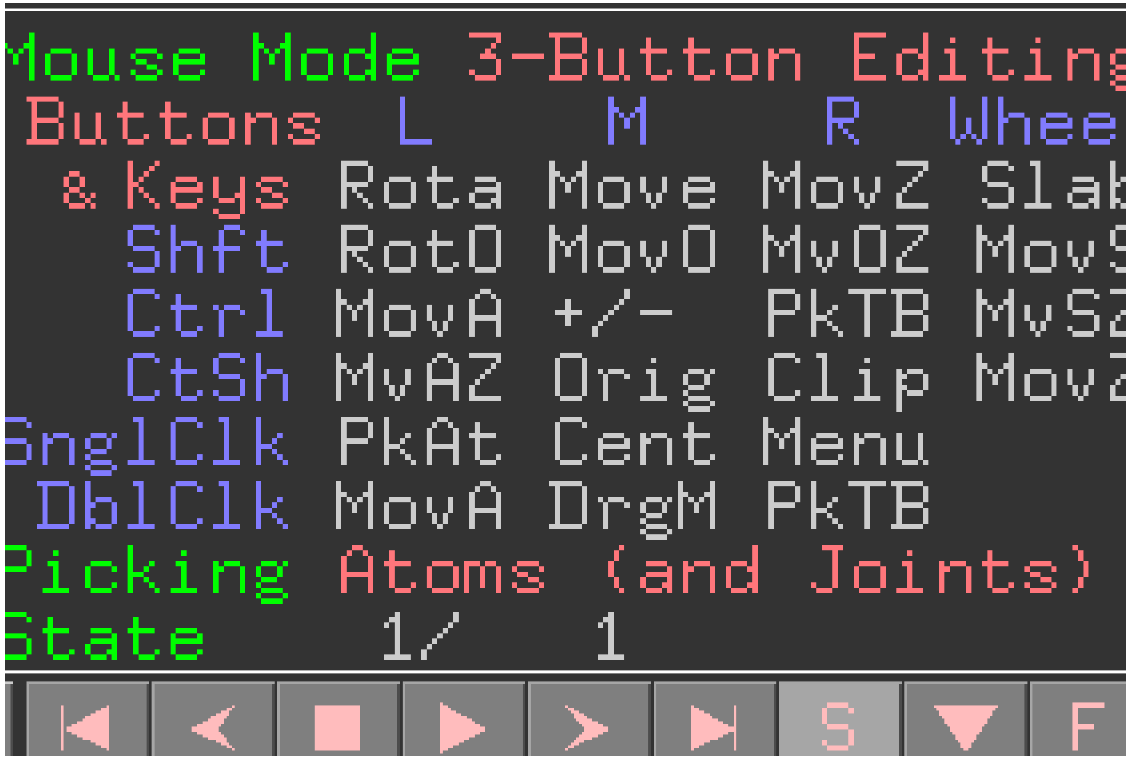

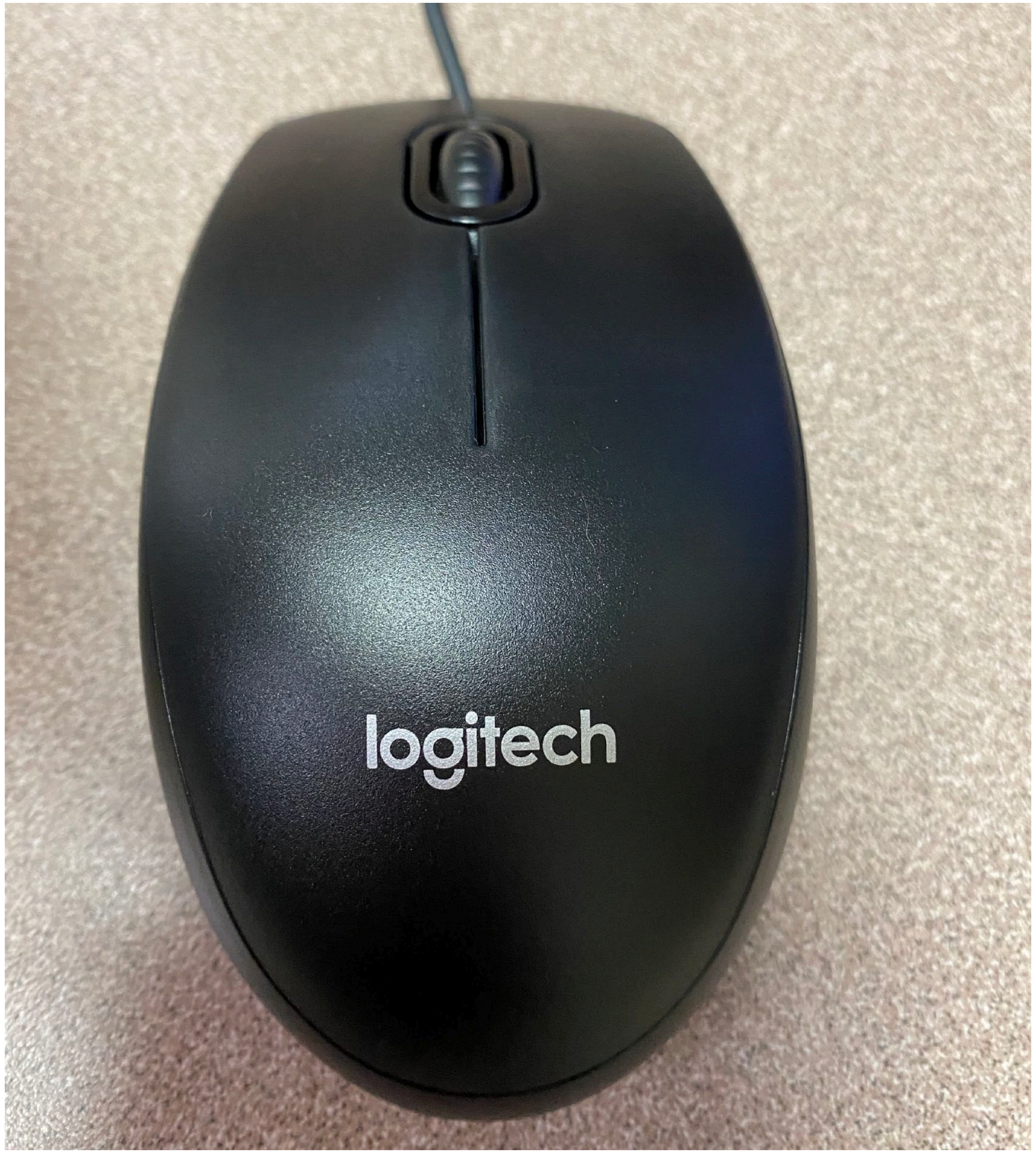

Label the metal binding site

Start by clicking on the Fe atom and labeling it. Repeat the same process for the rest of the binding site.

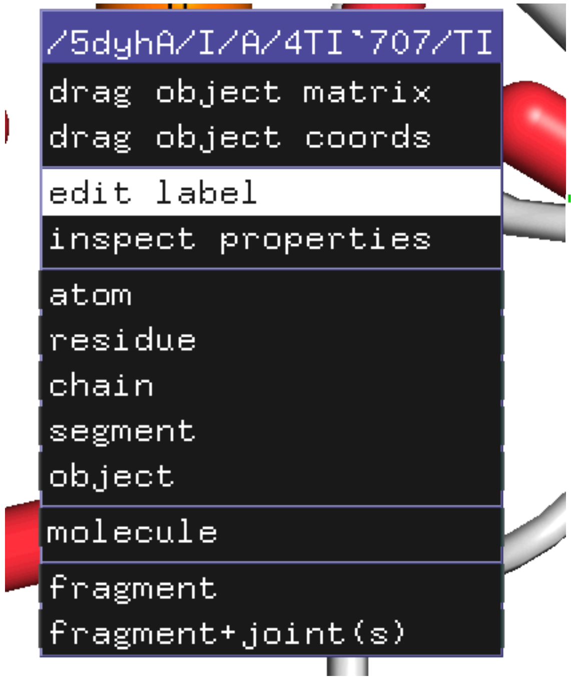

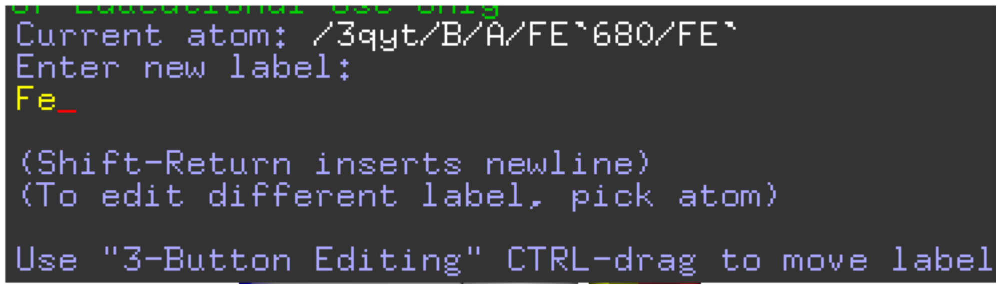

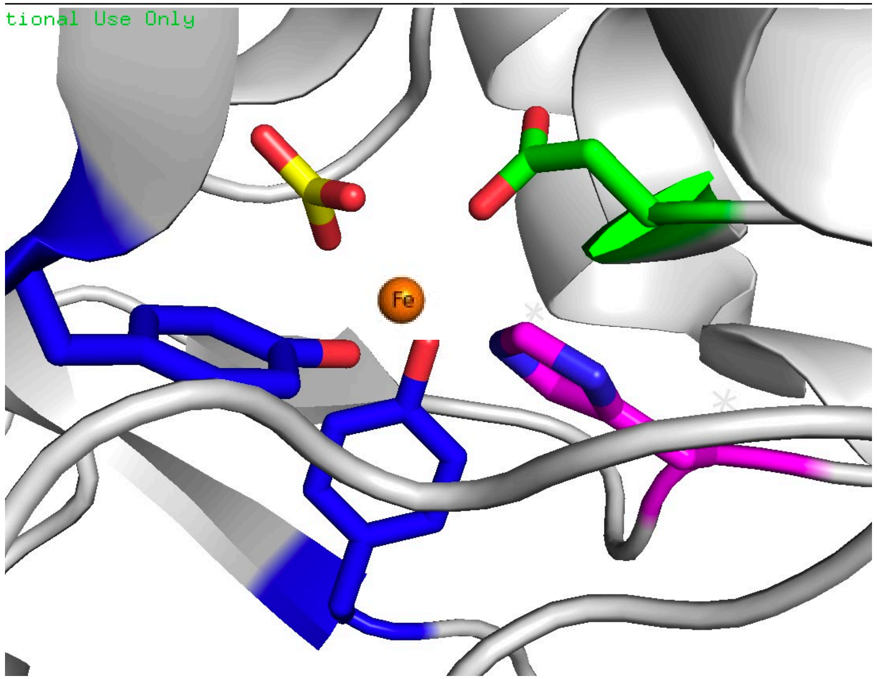

Label the metal binding site

Repeat the same process for the rest of the binding site.

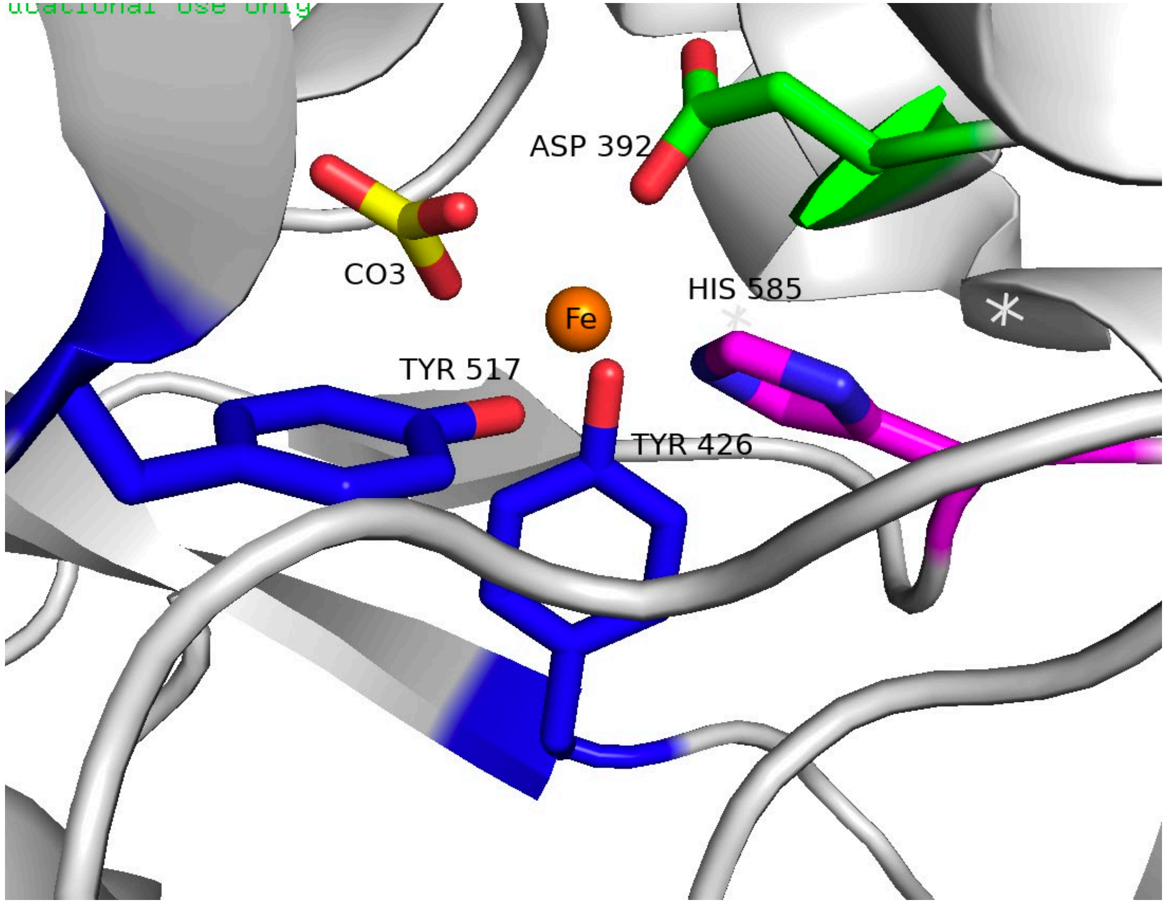

Label the bonds to Fe

1

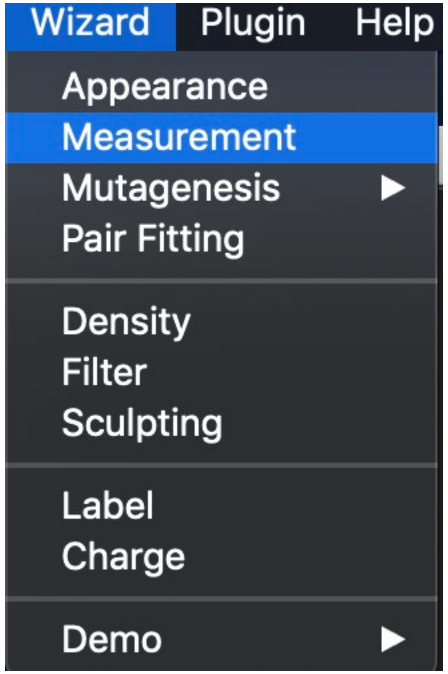

2

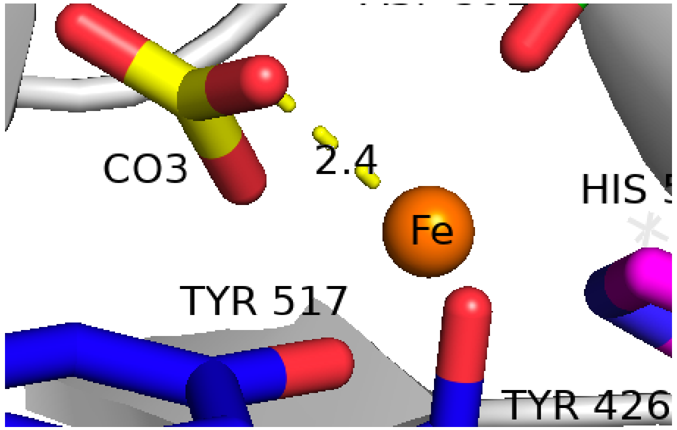

3

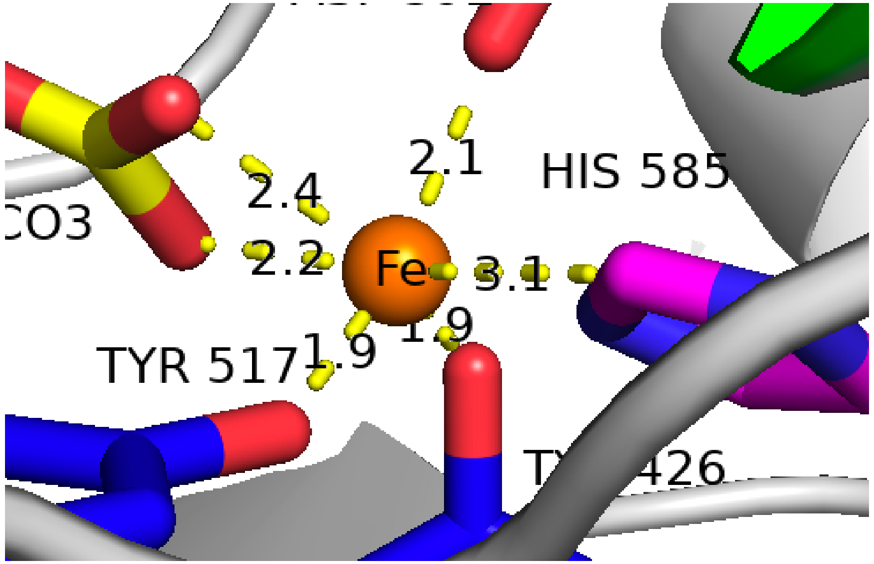

4

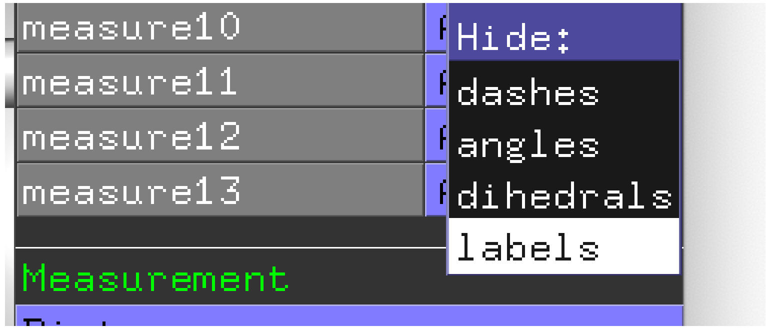

5

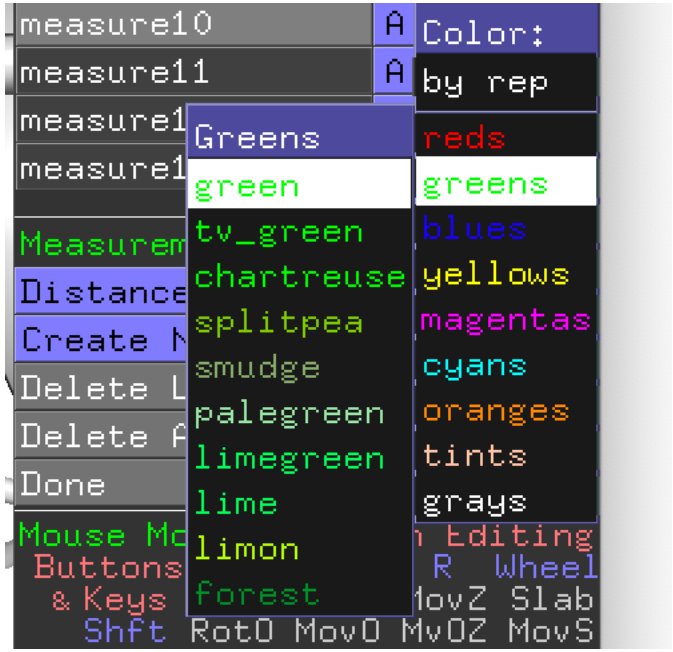

6

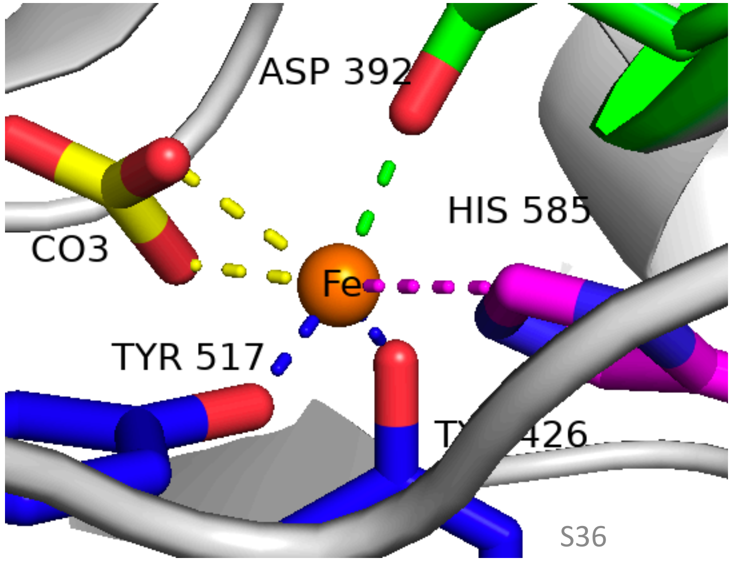

Optimize the figure

Dash_radius
0.08

Stick_radius
0.2

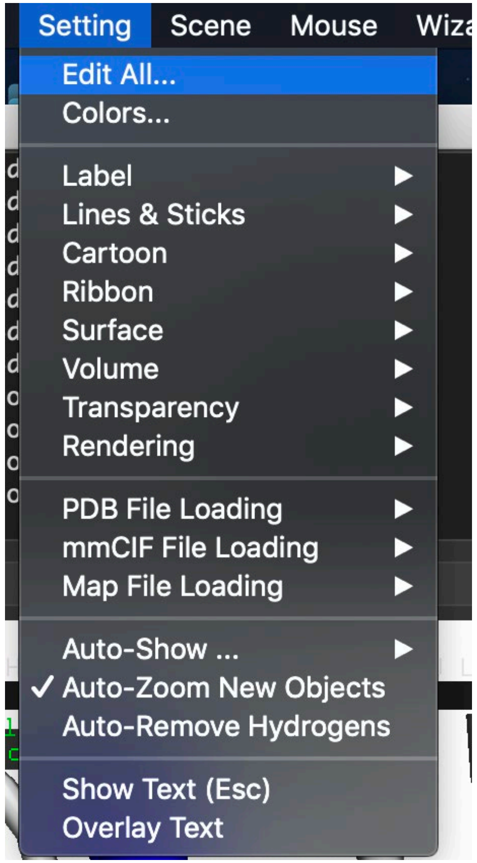

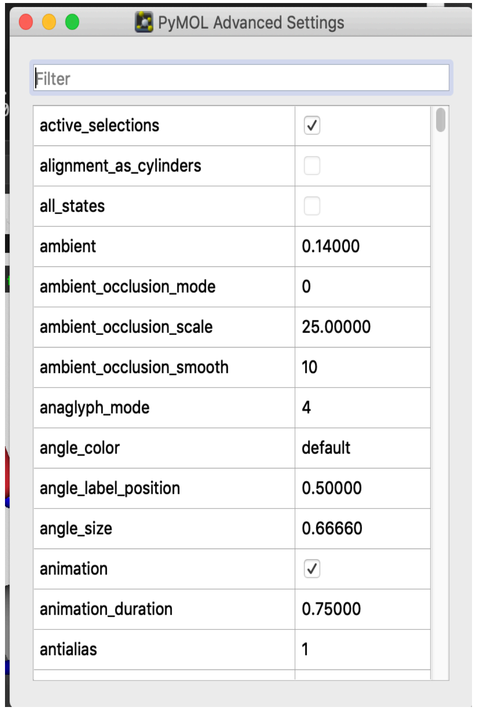

Optimize the figure

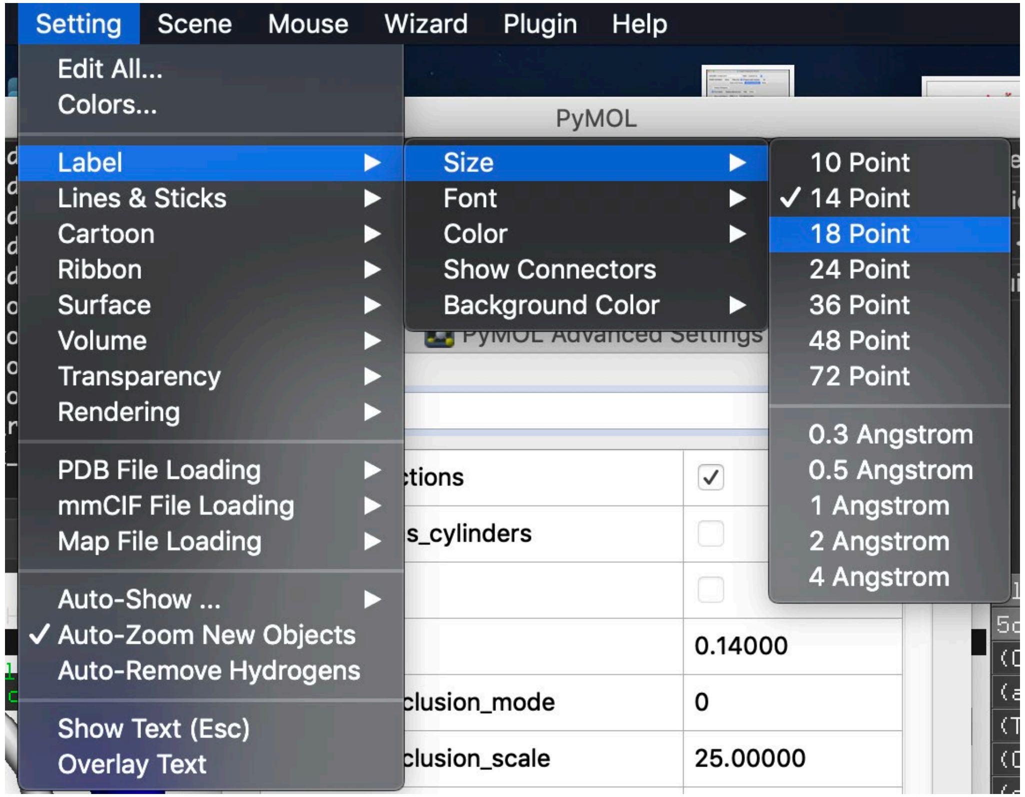

Font Size label 18

Final product

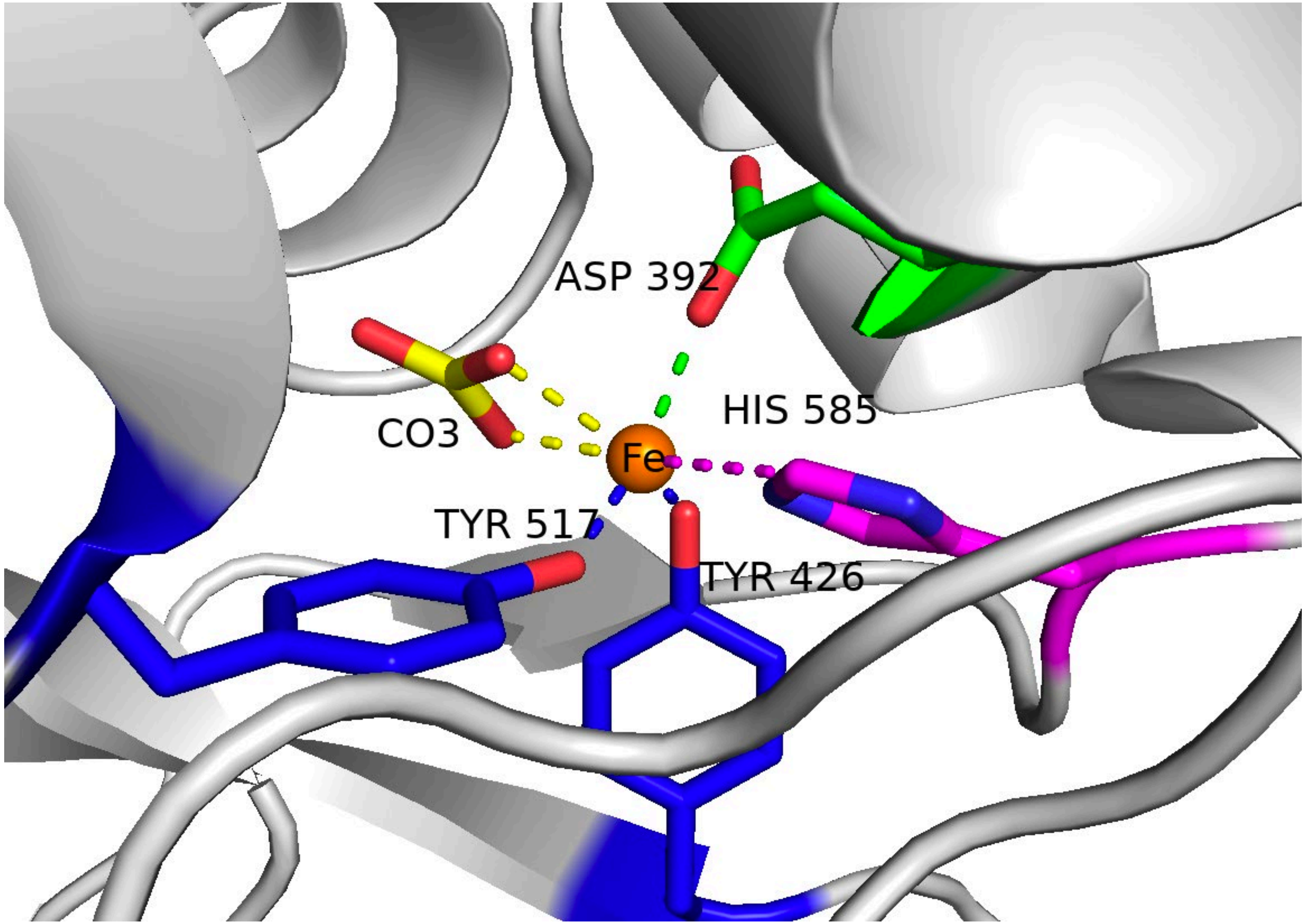

How to export the image

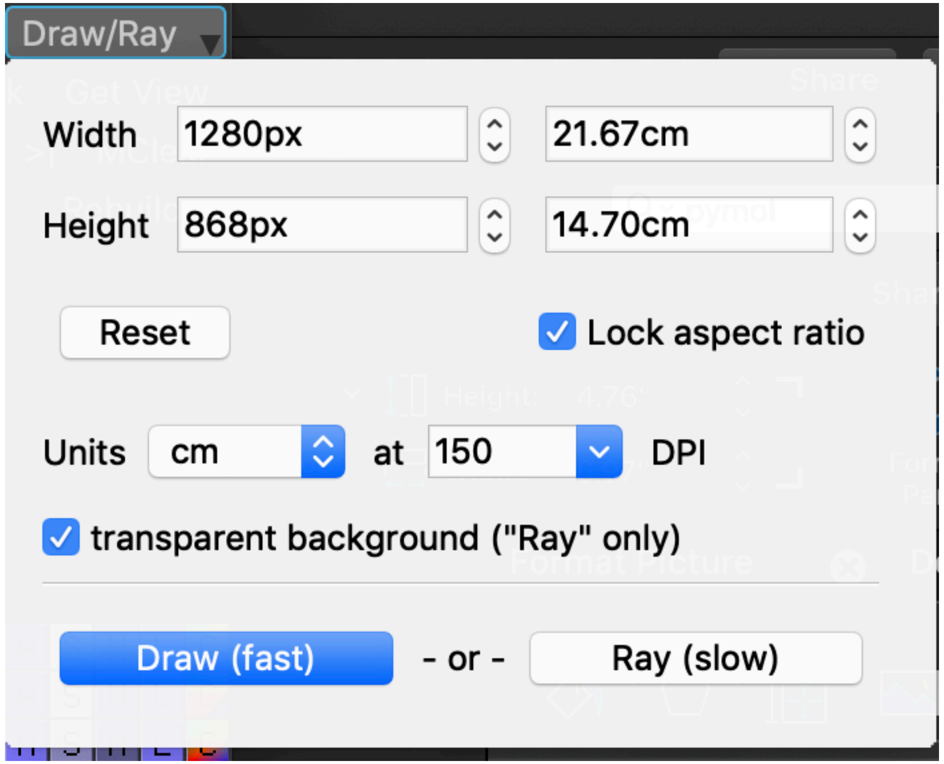

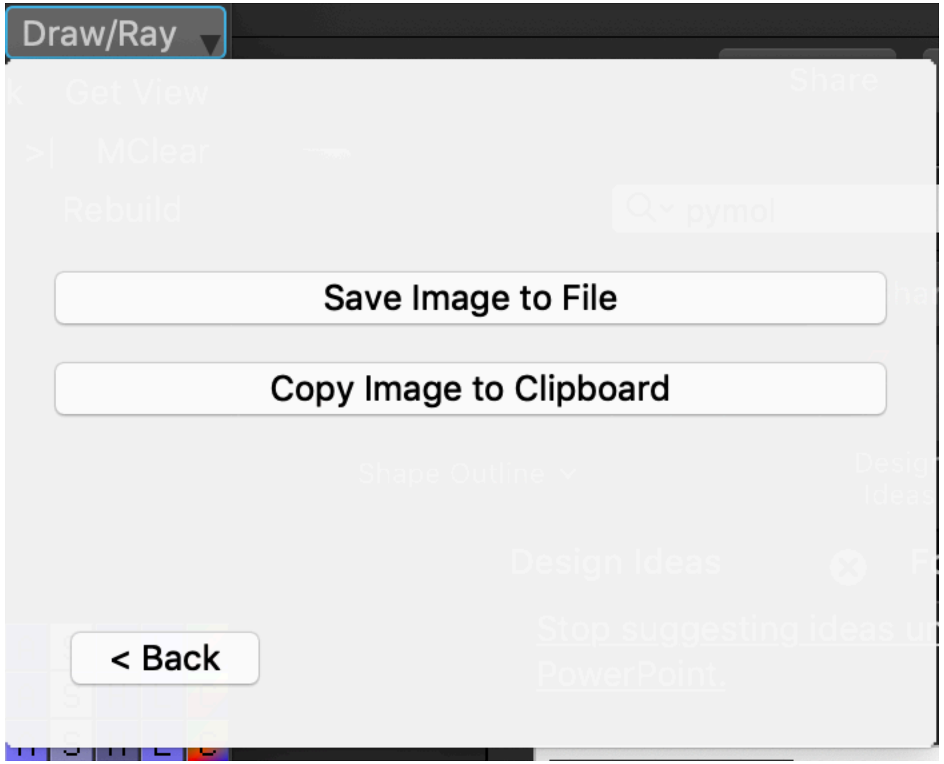

To make additional modifications to the visualization of the protein you can refer to this video.

- https://www.youtube.com/watch?v=wiKyOF-pGw4
