# Supplementary material for: Reinforcing Protein Biochemistry: A Two-Week Experiment Studying Iron(III) Binding by the Transferrin Protein through Stoichiometric Determination, Stability Analysis, and Visualization of the Binding Site
Source: J Chem Educ. 2024 Mar 26;101(4):1656–64. doi: 10.1021/acs.jchemed.3c01016 (PMC11033862; doi:10.1021/acs.jchemed.3c01016)
Supplement: Supplementary file 7 — ed3c01016_si_007.pdf [file ed3c01016_si_007.pdf]

Supporting Information

Reinforcing Protein Biochemistry: A Two-Week Experiment Studying Iron(III) Binding by the Transferrin Protein through Stoichiometric Determination, Stability Analysis, and Visualization of the Binding Site

Josué A. Benjamín-Rivera[1,ǂ] Mariela Pérez Otero[2,ǂ] Arthur D. Tinoco[1]*

[1]Department of Chemistry, University of Puerto Rico, Río Piedras Campus, Río Piedras, Puerto Rico 00931, United States.

[2]Department of Biology, University of Puerto Rico, Río Piedras Campus, Río Piedras, Puerto Rico 00931, United States.

[ǂ] Equal contribution

* Email: atinoco9278@gmail.com

Supporting Information G

Student Learning Assessment

Table of Content

	Page
I. Prelab Quiz	S2
II. Postlab Report	S3-S4

Pre-Lab Quiz

1. Serum transferrin is a transport protein that uses an endocytosis route to transport Fe(III) into cells. Briefly define endocytosis.

2. What are the four amino acids of sTf that coordinate Fe(III)?

3. Why is carbonate called the synergistic anion of sTf?

4. STf has a high affinity for Fe(III). This affinity is driven by the Lewis acidity of the metal ion. Explain.

5. How does Fe(III) binding stabilize the protein?

Post-Lab Report

Grading Scale: Total of 100 points

I. Data Analysis (30 points): 15 points for the processed data and 15 points for data analysis and interpretation.

A. Provide the image of your stained Urea gel. Properly label each lane and briefly explain what each lane shows in terms of protein stability.

B. Provide the stoichiometric curve by plotting the absorbance at 470 nm vs the Fe/Tf mole ratio. Calculate the relative extinction coefficient and explain why it deviates from the literature value.

C. Provide the PyMOL image of the Fe_C-sTf structure and briefly describe it.

II. Critical Analysis (70 points): 6 points for responding to each question correctly and 10 points for compositional format and analysis.

Prepare a short report in composition format in which you address the following questions. For some of these questions, we suggest you review the introductory lecture and the Benjamin et al. reference (2020).

1. Serum transferrin (STf) plays several important functions with respect to Fe(III) biodistribution throughout the body. What are these important functions?

2. STf has two Fe(III) binding sites; one in each lobe. Why is it that Fe(III) binding changes the conformation of each lobe?

3. Is the presence of carbonate a necessary component for sTf completing its coordination of Fe? Why?

4. What is the coordination geometry of Fe(III) located in the C-lobe of the crystal structure 3QYT? You may need to review coordination number and geometry introduced in General Chemistry.

5. What was the purpose of using membranes in the preparation of samples in part 1 of the experiment?

6. According to the stoichiometric curve that you have created, what is the stoichiometry of Fe(III) required to saturate sTf?

7. If the percentage of urea in the gel used to determine different species is increased from 7% to 10%, will the same separation of species still occur?

8. How does the closed conformation of the Fe(III)-bound sTf help the protein be recognized by transferrin receptor 1 (TfR1)?

9. During endocytosis, what is the function of clathrin?

10. In lab, you used PyMOL to visualize the Fe(III) coordination in the C-lobe of the protein. Now construct the Fe(III) coordination in the N-lobe of the protein using coordination details from the lecture presentation. Why is this coordination referred to as non-canonical binding? How does this image serve as a model of a chelator removal of Fe(III) from the protein during endocytosis?

Reference

Benjamín-Rivera, J. A.; Cardona-Rivera, A. E.; Vázquez-Maldonado, Á. L.; Dones-Lassalle, C. Y.; Pabón-Colon, H. L.; Rodríguez-Rivera, H. M.; Rodríguez, I.; González-Espiet, J. C.; Pazol, J.; Pérez-Ríos, J. D.; et al. Exploring serum transferrin regulation of nonferric metal therapeutic function and toxicity. *Inorganics* **2020**, *8* (9), 48.
