# Supplementary material for: Reinforcing Protein Biochemistry: A Two-Week Experiment Studying Iron(III) Binding by the Transferrin Protein through Stoichiometric Determination, Stability Analysis, and Visualization of the Binding Site
Source: J Chem Educ. 2024 Mar 26;101(4):1656–64. doi: 10.1021/acs.jchemed.3c01016 (PMC11033862; doi:10.1021/acs.jchemed.3c01016)
Supplement: Supplementary file 8 — ed3c01016_si_008.pdf [file ed3c01016_si_008.pdf]

Supporting Information

Reinforcing Protein Biochemistry: A Two-Week Experiment Studying Iron(III) Binding by the Transferrin Protein through Stoichiometric Determination, Stability Analysis, and Visualization of the Binding Site

Josué A. Benjamín-Rivera[1,ǂ] Mariela Pérez Otero[2,ǂ] Arthur D. Tinoco[1]*

[1]Department of Chemistry, University of Puerto Rico, Río Piedras Campus, Río Piedras, Puerto Rico 00931, United States.

[2]Department of Biology, University of Puerto Rico, Río Piedras Campus, Río Piedras, Puerto Rico 00931, United States.

ǂ Equal contribution

* Email: atinoco9278@gmail.com

Supporting Information H

Student Activity Assessment

Table of Content

	Page
I. Student Evaluation Survey	S2-S3
II. Focal Group Questions	S4

INSTRUCTIONS

Please circle your response to the items. Rate aspects of the activity on a 1 to 5 scale:

1 = “Strongly disagree,” or the lowest, most negative impression
2 = “Disagree,” or a low, negative impression
3 = “Neither agree nor disagree,” or an adequate impression
4 = “Agree,” or a high, positive impression
5 = “Strongly agree,” or the highest, most positive impression

Choose N/A if the item is not appropriate or does not apply to this activity.
Your feedback is sincerely appreciated. Thank you.

ACTIVITY CONTENT (Circle your response to each item)

1. This experiment has helped me to develop my data interpretation skills.
   < 5 4 3 2 1 N/A >
2. This experiment has helped me to develop my laboratory skills.
   < 5 4 3 2 1 N/A >
3. I understand the importance of this experiment to my chemistry studies.
   < 5 4 3 2 1 N/A >
4. Completing this experiment has increased my knowledge of chemistry.
   < 5 4 3 2 1 N/A >

ACTIVITY DESIGN (Circle your response to each item)

5. It was clear to me how this laboratory exercise would be assessed.
   < 5 4 3 2 1 N/A >
6. Sufficient background information was provided in the introduction.
   < 5 4 3 2 1 N/A >
7. The experimental procedure was clearly explained in the manual.
   < 5 4 3 2 1 N/A >

ACTIVITY INSTRUCTORS (Circle your response to each item)

8. The instructors offered effective support and guidance.
   < 5 4 3 2 1 N/A >
9. The instructors were well prepared.
   < 5 4 3 2 1 N/A >

OVERALL ACTIVITY (Circle your response to each item)

10. The time available to complete this experiment was
    < 5 4 3 2 1 N/A >
11. Overall, as a learning experience, I will rate this experiment as
    < 5 4 3 2 1 N/A >
12. I accomplished the objectives of this activity
    < 5 4 3 2 1 N/A >
13. How would you improve this activity? (Check all that apply)

____ Provide better information before the activity.
____ Clarify the activity objectives.
____ Reduce the content covered in the activity.
____ Increase the content covered in the activity.
____ Improve the instructional methods.
____ Improve the activity organization.
____ Make the activity less difficult.
____ Make the activity more difficult.
____ Slow down the pace of the activity.
____ Speed up the pace of the activity.
____ Increase time for the activity.
____ Shorten the time for the activity.
____ Additional suggestions

____
____
____
____
____
____

ADDITIONAL QUESTIONS

14. Did you enjoy the experiment? Why or why not?

____
____
____

15. What was the main idea of this experiment?

____
____
____

16. What aspects of the experiments do you find the most enjoyable and interesting?

____
____
____

17. Any additional comment on that experience.

____
____
____

Focal Group Questions

QUESTIONS

1. How does this activity contribute to your understanding of the concept of a protein metal binding site?
2. How would you improve this activity?
3. Considering your experience, how likely would you recommend this activity to become part of a biochemistry lab course?
