# Supplementary material for: Reinforcing Protein Biochemistry: A Two-Week Experiment Studying Iron(III) Binding by the Transferrin Protein through Stoichiometric Determination, Stability Analysis, and Visualization of the Binding Site
Source: J Chem Educ. 2024 Mar 26;101(4):1656–64. doi: 10.1021/acs.jchemed.3c01016 (PMC11033862; doi:10.1021/acs.jchemed.3c01016)
Supplement: Supplementary file 9 — ed3c01016_si_009.pdf [file ed3c01016_si_009.pdf]

Supporting Information

Reinforcing Protein Biochemistry: A Two-Week Experiment Studying Iron(III) Binding by the Transferrin Protein through Stoichiometric Determination, Stability Analysis, and Visualization of the Binding Site

Josué A. Benjamín-Rivera[1,ǂ] Mariela Pérez Otero[2,ǂ] Arthur D. Tinoco[1]*

[1]Department of Chemistry, University of Puerto Rico, Río Piedras Campus, Río Piedras, Puerto Rico 00931, United States.

[2]Department of Biology, University of Puerto Rico, Río Piedras Campus, Río Piedras, Puerto Rico 00931, United States.

[ǂ] Equal contribution

* Email: atinoco9278@gmail.com

Supporting Information I

Visualization of Raw Data and Survey Response Distribution

Table of Content

	Page
Tables	S2-S3

Note: To identify the specific survey questions associated with the presented data, please refer to Supporting Document H for a comprehensive view of the survey questionnaire.

Table S H1. Response to specific Likert-scale questions of the 12 volunteers during the focal group sessions that are included in **Figure 7**.

	Strongly agree	Agree	Neither agree nor disagree	Disagree	Strongly disagree
Question 5	8	4	0	0	0
Question 6	12	0	0	0	0
Question 7	0	8	4	0	0
Question 10	6	6	0	0	0

Table S H2. Response to Likert-scale questions of the 16 laboratory course students during the focal group sessions that are included in **Figures 8-10**.

	Strongly agree	Agree	Neither agree nor disagree	Disagree	Strongly disagree
Question 1	11	5	0	0	0
Question 2	16	0	0	0	0
Question 3	16	0	0	0	0
Question 4	14	2	0	0	0
Question 8	14	2	0	0	0
Question 9	16	0	0	0	0
Question 11	16	0	0	0	0
Question 12	11	5	0	0	0
